# Tanjungides A and B: New Antitumoral Bromoindole Derived Compounds from *Diazona* cf *formosa*. Isolation and Total Synthesis

**DOI:** 10.3390/md12021116

**Published:** 2014-02-21

**Authors:** Carmen Murcia, Laura Coello, Rogelio Fernández, María Jesús Martín, Fernando Reyes, Andrés Francesch, Simon Munt, Carmen Cuevas

**Affiliations:** 1 Medicinal Chemistry Department, PharmaMar S.A., Avda. de los Reyes 1, Pol. In. La Mina, Colmenar Viejo, Madrid 28770, Spain; E-Mails: cmurcia@pharmamar.com (C.M.); lcoello@pharmamar.com (L.C.); rfernandez@pharmamar.com (R.F.); mjmartin@pharmamar.com (M.J.M.); afrancesch@pharmamar.com (A.F.); smunt@pharmamar.com (S.M.); 2 Fundación MEDINA, Avda. del Conocimiento 3, Parque Tecnológico de Ciencias de la Salud, Granada 18016, Spain; E-Mail: fernando.reyes@medinaandalucia.es

**Keywords:** bromoindole, tunicate, *Diazona* cf *formosa*, cytotoxicity, isolation, structure elucidation, total synthesis

## Abstract

Tanjungides A (**1**) (*Z* isomer) and B (**2**) (*E* isomer), two novel dibrominated indole enamides, have been isolated from the tunicate *Diazona* cf *formosa*. Their structures were determined by spectroscopic methods including HRMS, and extensive 1D and 2D NMR. The stereochemistry of the cyclised cystine present in both compounds was determined by Marfey’s analysis after chemical degradation and hydrolysis. We also report the first total synthesis of these compounds using methyl 1*H*-indole-3-carboxylate as starting material and a linear sequence of 11 chemical steps. Tanjungides A and B exhibit significant cytotoxicity against human tumor cell lines.

## 1. Introduction

Ascidians [1] are a rich source of bromoindole derived metabolites such as eudistomin [2], didemnimide [3], meridianin [4], coscinamide [5], rhopaladin [6], kottamide [7,8] and aplicyanin [9]. Most of these compounds exhibit antiviral, antibacterial and anti-inflammatory activity as well as cytotoxicity against tumor cell lines. The diazonamides isolated from *Diazona angulata* (originally misidentified as *Diazona chinensis*) [10] and *Diazona* sp. [11] provide a further example of secondary metabolites from ascidians. Strong cytotoxic activity has been reported for these compounds with IC_50_ values in the nanomolar range. As part of work to study marine organisms from Indonesia, we have examined the constituents of the tunicate *Diazona* cf *formosa* collected off the coast of Tanjung Liarua and Toro Doro (Timor Island). In this paper we report the isolation, structure elucidation and synthesis of two new indole alkaloids Tanjungides A and B (**1** and **2**). Tanjungides are novel alkaloids containing a dibromoindole joined to a disulfide dipeptide by an enamide bond.

## 2. Results and Discussion

### 2.1. Isolation and Structure Elucidation

Cytotoxicity bioassay-guided fractionation of an organic extract of the organism, including VLC RP-18 chromatography followed by reverse-phase preparative HPLC of selected active fractions, led to the isolation of Tanjungides A and B.

**Figure 1:**
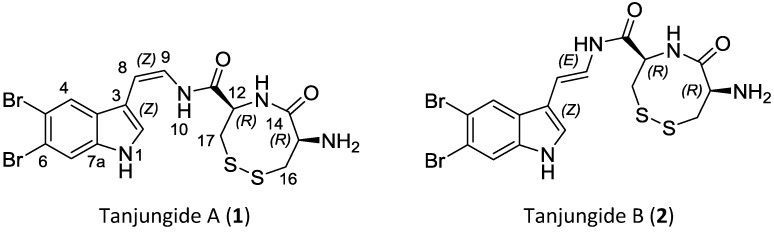
Chemical Structures of Tanjungides.

**Figure 2:**
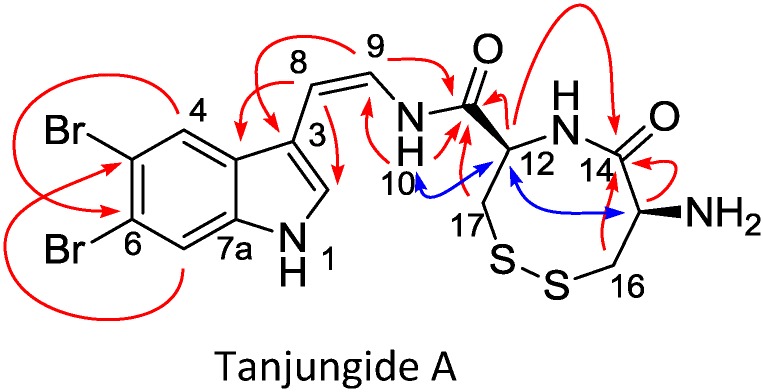
Selected HMBC (H → C, red) and ROESY (blue) correlations for Tanjungide A.

Compound **1** was isolated as an optically active pale yellow amorphous solid with a pseudomolecular ion in the (+)-HRESIMS at *m/z* 518.9142 and an isotopic cluster consistent with the presence of two bromine atoms. The presence of 16 signals in the ^13^C NMR spectrum (Table 1) was also in agreement with the molecular formula C_16_H_16_^79^Br_2_N_4_O_2_S_2_ (*m/z* 518.9142 [M + H]^+^, calcd. for C_16_H_17_^79^Br_2_N_4_O_2_S_2_, 518.9154). The presence of a 3,5,6-trisubstituted indole in 1 (Figure 1) was inferred by the existence of four characteristic signals in the low field region of the ^1^H NMR spectrum in DMSO-*d*_6_, two doublets at δ_H_ 7.78 (d, H-2, *J* = 2.4 Hz) and 11.78 (d, NH-1, *J* = 2.6 Hz) and two singlets at δ_H_ 7.81 (s, H-7) and δ_H_ 8.01 (s, H-4). In addition, the two bromine atoms contained in the molecular formula were located at C-5 and C-6 based on their ^13^C chemical shifts. The intense 3-bond long range couplings between H-4 and C-6 at δ_C_ 115.7 ppm and between H-7 and C-5 at δ_C_ 113.5 ppm observed in the HMBC spectrum further confirmed the chemical shifts of these two quaternary carbons. The nature of the substituent at C-3 was deduced from analysis of additional signals in the low field region of the ^1^H NMR spectrum and correlations observed in the COSY, HSQC and HMBC spectra. A spin system comprising two olefinic signals at δ_H_ 6.06 ppm (H-8) and 6.68 ppm (H-9), and an interchangeable proton at δ_H_ 9.60 ppm (NH-10) established the presence of an enamide. A coupling constant of 9.4 Hz between H-8 and H-9 confirmed a *Z* geometry for this double bond. Finally, HMBC correlations from H-9 to C-3 (δ_C_ 109.2 ppm) and from H-8 to C-2 (δ_C_ 126.8 ppm), and C-3a (δ_C_ 127.6 ppm) indicated that the indole moiety was substituted at C-3 with a *Z* geometry enamide fragment. The remaining atoms, C_6_H_9_N_2_O_2_S_2_, comprised two carbonyl (δ_C_ 169.9 and 167.1 ppm), two methine, (δ_C_ 52.5/δ_H_ 5.02 ppm and δ_C_ 51.2/δ_H_ 4.65 ppm) and two methylene groups (δ_C_ 41.6/δ_H_ 3.40 and 2.86 ppm and δ_C_ 39.7/δ_H_ 3.17 and 2.94 ppm) with three degrees of unsaturation being required for this molecular formula, including the two carbonyls mentioned previously. Analysis of the bidimensional spectra revealed the presence of a two spin system corresponding to two consecutive cysteine residues. Cross-peaks observed in the HMBC experiment between H-12 and H-16 and carbon C-14 at δ_C_ 169.9 ppm (Figure 2) confirmed this structural proposal. Furthermore, correlations observed in the HMBC experiment between H-9, NH-10, H-12 and H-17 to C-11, and a ROESY correlation between NH-10 and H-12, connected these cysteines residues to the enamide group through C-11. Finally, linkage of the two cysteine amino acids by a S–S bond to form a cyclic cystine explained the remaining unsaturation present and established the complete structure of Tanjungide A.

**Table 1:** ^1^H and ^13^C NMR (500 and 125 MHz) assignments for Tanjungide A (**1**) (DMSO-*d*_6_) and Tanjungide B (**2**) (CD_3_OD).

Position	Tanjungide A (1)		Tanjungide B (2)	
	δ_H_ (m, *J* in Hz)	δ_C_, mult.	δ_H_ (m, *J* in Hz)	δ_C_, mult.
**1**	11.78 (d, 2.6)	-	-	-
**2**	7.78 (d, 2.4)	126.8, d	7.35 (s)	126.7, d
**3**	-	109.2, s	-	112.9, s
**3a**	-	127.6, s	-	127.4, s
**4**	8.01 (s)	123.0, d	8.04 (s)	124.6, d
**5**	-	113.5, s	-	117.5, s
**6**	-	115.7, s	-	115.5, s
**7**	7.81 (s)	116.3, d	7.72 (s)	117.4, d
**7a**	-	135.4, s	-	138.3, s
**8**	6.06 (d, 9.4)	103.8, d	6.50 (d, 14.8)	109.2, d
**9**	6.68 (dd, 9.4, 9.8)	118.9, d	7.34 (d, 14.8)	120.7, d
**10**	9.60 (d, 9.8)	-	-	-
**11**	-	167.1, s	-	167.8, s
**12**	5.02 (ddd, 11.8, 11.5, 3.6)	52.5, d	4.93 (m)	54.2, d
**13**	8.44 (br s)	-	-	-
**14**	-	169.9, s	-	169.0, s
**15**	4.65 (m)	51.2, d	4.60 (m)	53.7, d
**16**	2.94 (dd, 14.2, 11.5) 3.17 (m)	39.7, t	3.11 (m)	41.8, t
**17**	2.86 (dd, 13.3, 11.6) 3.40 (m)	41.6, t	2.90 (dd, 12.4, 12.4) 3.47 (m)	43.2, t

The absolute configuration of **1** was determined by converting the cyclized cystine into two alanines by Raney^®^-Nickel desulfurization [12]. The absolute configuration of the resulting Ala amino acids was determined to be *R* by comparing the hydrolysis products of **1** with appropriate amino acid standards using HPLC-MS chromatography and after derivatization with Marfey’s reagent l-FDAA (*N*α-(2,4-dinitro-5-fluorophenyl)-l-alaninamide) [13].

Compound **2** (Figure 1) was isolated as an optically active pale yellow amorphous solid with the same molecular formula as **1** [(+)-HRESIMS *m/z* 518.9142 [M + H]^+^ (Calcd. for C_16_H_17_^79^Br_2_N_4_O_2_S_2_, 518.9154)]. After examination of the 1D and 2D NMR spectra we concluded that Tanjungide B (Table 1) was very similar to Tanjungide A, and the major difference found in the ^1^H NMR was the value of the coupling constant of the ∆^8^ olefin signals. Thus, the coupling constant *J*_H8-H9_ had a value of 14.6 Hz corresponding to a *E* geometry for the double bond. The absolute configuration of the Cys residues was not determined due to the small amount of compound isolated and was assumed to be the same as in Tanjungide A (**1**). The validity of this assumption was later confirmed by total synthesis of the molecule.

### 2.2. Biological Activities of Tanjungides A and B

The cytotoxic activity of the new compounds (Table 2) was tested against three human tumour cell lines, lung (A549), colon (HT29), and breast (MDA-MB-231), following a published procedure [14]. Tanjungide A (**1**) exhibited strong activity with GI_50_ values in the range 0.19 to 0.33 μM, whereas Tanjungide B (**2**) displayed only mild cytotoxicity, with GI_50_ values ranging from 1.00 to 2.50 μM.

**Table 2:** Cytotoxic Activity Data (μM) of Compounds **1** and **2**.

Compound	Lung-NSCLC	Colon	Breast
	A549	HT29	MDA-MB-231
	GI_50_	GI_50_	GI_50_
Natural Tanjungide A	0.33	0.19	0.23
Synthetic Tanjungide A	0.33	0.25	0.19
Natural Tanjungide B	2.50	2.31	1.63
Synthetic Tanjungide B	1.00	1.15	1.11

### 2.3. Total Synthesis of Tanjungides A and B

**Figure 3:**
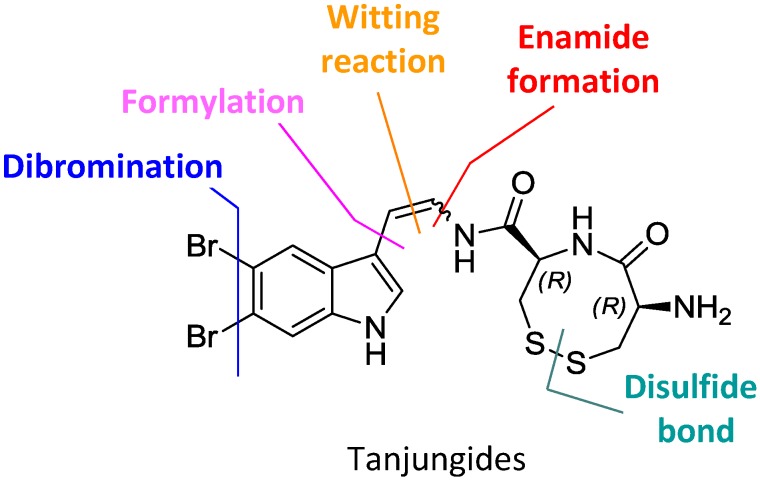
Retrosynthetic analysis of Tanjungides A and B.

In order to solve the supply problem for these two new marine chemical entities and progress pharmaceutical development and *in vivo* preclinical studies, we have completed the first total synthesis of Tanjungides A and B. This synthesis uses methyl 1*H*-indole-3-carboxylate as starting material and involves a linear sequence of 11 chemical steps. Key elements of our approach include selective dibromination of the indole, formylation by Vilsmeier reaction, Wittig olefination, stereoselective enamide formation and oxidation to create the disulfide bond (Figure 3). The strategy uses vinyl iodide indole **8** as a common precursor to give both Tanjungides.

**Scheme 1:**
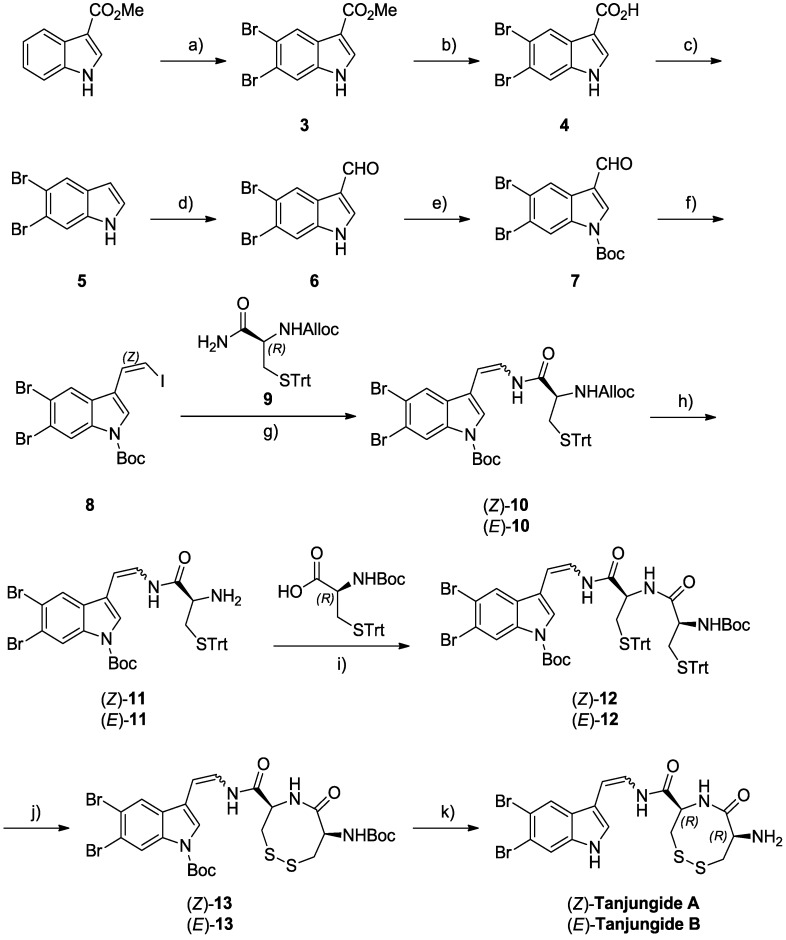
Total synthesis of Tanjungides A (**1**) and B (**2**).

The synthesis started, as outlined in Scheme 1, from the cheap commercially available methyl 1*H*-indole-3-carboxylate as this provided a high yielding route to a 5,6-dibrominated indole possessing an aldehyde moiety at C3, a highly versatile building block for the total synthesis of the two natural products. The slow addition of two equivalents of bromine to methyl 1*H*-indole-3-carboxylate in acetic acid at 23 °C yielded the corresponding 5,6-dibromo intermediate **3** as a single pure product in 66% yield [15]. Disappointingly, attempted methyl ester reduction of **3** to give aldehyde **6** directly was unsuccessful, and an alternative stepwise process to give aldehyde **6** was used involving hydrolysis and decarboxylation to give 5,6-dibromo-1*H*-indole **5** in good yield (90% over two steps) followed by Vilsmeier formylation using dimethylformamide and phosphorus oxychloride. After protection of the indole nitrogen as a *tert*-butyl carbamate, Wittig olefination with (iodomethyl)triphenylphosphonium iodide [16] gave the desired vinyl iodide indole **8** in 83% yield and as a 9:1 ratio of *Z*:*E* isomers [17].

With vinyl iodide **8** in hand, the next step involved coupling of the suitably-protected cysteine amino acids (Scheme 1). As described by Buchwald and co-workers [18], depending on the conditions used for the coupling reaction, vinyl iodide **8** provided access to both stereoisomers of enamide **10** and hence to both Tanjungide A and B. Specifically, copper-catalyzed reaction of **8** with *N*-allyloxycarbonyl-*S*-trityl-l-cysteine-amide **9**, made in one step from commercially available *S*-trityl-l-cysteine-amide, gave enamide **10** in moderate yield (50%−60%) with use of Cs_2_CO_3_ as base affording mainly enamide (*Z*)-**10**, which could be readily separated from the corresponding (*E*)-isomer by column chromatography, and K_2_CO_3_ providing predominantly enamide (*E*)-**10**. Next, removal of the Alloc group of (*Z*)-**10** or (*E*)-**10** under neutral conditions using Pd(PPh_3_)_4_ and PhSiH_3_ and coupling of the resulting primary amine with (*N*-(*tert*-butoxycarbonyl)-*S*-trityl-l-cysteine) by treatment with HATU and HOBt yielded the corresponding amide (*Z*)-**12** or (*E*)-**12**. After substantial experimentation, the trityl group proved to be the best thiol protecting group for each of the cysteine amino acid building blocks. To complete the synthesis, the key cyclization of **12** to form the disulfide bond was accomplished using I_2_ in CH_2_Cl_2_:CH_3_OH at high dilution to avoid undesired side-products [19,20] and subsequent simultaneously cleavage of both Boc protecting groups of **13** with TFA gave Tanjungides A (**1**) and B (**2**). All the spectroscopic data (^1^H and ^13^C NMR, optical rotation, IR, *etc*.), HPLC retention times and biological activities of the synthetic samples exactly matched those of the isolated natural products. The Supplementary Information provides more details.

Regents and conditions: (a) Br_2_, AcOH, 23 °C, 2 h, 66%; (b) aq NaOH 2 M, CH_3_OH, reflux, 2.5 h, 95%; (c) Pyridine, reflux, 12 h, 95%; (d) *i* POCl_3_, DMF, 35 °C, 1 h then 65 °C, 1 h, *ii* aq NaOH 2 M, 110 °C, 5 min, 92%; (e) (Boc)_2_O, DMAP, 1,4-Dioxane, 23 °C, 2 h, 86%; (f) Iodomethyltriphpenylphosphonium iodide, NaHMDS 1.0 M, THF, −78 °C, 2 h, 83%; (g) for (*Z*)-**10**: **9**, Cul, Cs_2_CO_3_, DMEDA, THF, 60 °C, 18 h, 50% + 13% (*E*)-**10**; for (*E*)-**10**: **9**, Cul, K_2_CO_3_, DMEDA, THF, 80 °C, 18 h, 60% + 14% (*Z*)-**10**; (h) Pd(PPh_3_)_4_, PhSiH_3_, CH_2_Cl_2_, 23 °C, 30 min, 85% (*Z*)-**11** and 72% (*E*)-**11**; (i) *N*-Boc-l-(*S*-trityl)-Cys, HATU, HOBt, DIPEA, CH_2_Cl_2_:DMF (4:1), 23 °C, 2 h, 83% (*Z*)-**12** and 62% (*E*)-**12**; (j) I_2_, CH_2_Cl_2_:CH_3_OH (10:1), 23 °C, 40 min, 84% (*Z*)-**13** and 73% (*E*)-**13**; (k) TFA, CH_2_Cl_2_, 0 °C, 3 h, 60% (*Z*)-**Tanjungide A** and 45% (*E*)-**Tanjungide B**.

## 3. Experimental Section

### 3.1. General

Dry solvents were purchased and used without any extra processing. All reagents were used as purchased without further purification unless otherwise stated. All reactions were performed under an atmosphere of nitrogen in flame dried or oven dried glassware. Routine monitoring of reactions was performed using silica gel TLC plates (Merck 60 F254, Merck KGaA, Darmstadt, Germany). Spots were visualized by UV and/or dipping the TLC plate into an ethanolic phosphomolybdic acid solution and heating with a hot plate. Flash chromatography was carried out on silica gel 60 (200–400 mesh). ^1^H and ^13^C NMR were recorded on a Varian Unity 300 or 500 spectrometer at 300 or 500, and 75 or 125 MHz, respectively. Chemical Shifts (δ) are reported in parts per millions (ppm) referenced to CHCl_3_ at 7.26 ppm for ^1^H and CDCl_3_ at 77.0 ppm for ^13^C, to CH_3_OH at 3.30 ppm for ^1^H and CD_3_OD at 49.0 ppm; and to (CH_3_)_2_SO at 2.50 ppm for ^1^H and (CD_3_)_2_SO at 39.5 ppm for ^13^C. Coupling constants are reported in Hertz (Hz), with the following abbreviations used: s = singlet, d = doublet, t = triplet, q = quartet, m = multiplet. When appropriate, the multiplicities are preceded with br, indicating that the signal was broad. Optical rotations were determined using a Jasco P-1020 polarimeter (Jasco Inc., Easton, MD, USA) with a sodium lamp and are reported as follows: [α]^25^_D_ (*c* g/100 mL, solvent). (+)-HRESIMS was performed on an Applied Biosystems QStar pulsar Analyzer spectrometer (Applied Biosystems Inc., Foster City, CA, USA) employing 0.1% of formic acid in methanol as an ionic mobile phase. (+)-ESIMS were recorded using an Agilent 1100 Series LC/MSD spectrometer (Agilent Technologies, Santa Clara, CA, USA). UV spectra were performed using an Agilent 8453 UV-VIS spectrometer (Agilent Technologies). IR spectra were obtained with a Perkin Elmer Spectrum 100 FT-IR spectrometer (PerkinElmer Inc., Waltham, MA, USA) with ATR sampling.

### 3.2. Animal Material

The tunicate *Diazona* cf *formosa* (Order Phlebobranchia, Family Diazonidae, Genus Diazona) was collected by hand using a rebreather diving system in East Timor (08°25.637′*S*/126°22.849′*E*) at depths ranging between 6 and 80 m in June 2009. A sample of the specimen was deposited in the Center for the Advanced Studies of Blanes in Girona, Spain, with the reference code TISM-763.

### 3.3. Extraction and Isolation

A specimen of *Diazona* cf *formosa* (128 g) was triturated and exhaustively extracted with CH_3_OH:CH_2_Cl_2_ (50:50, 3 × 200 mL). The combined extracts were concentrated to yield a crude mass of 5.05 g that was subjected to VLC on Lichroprep RP-18 (Merck KGaA) with a stepped gradient from H_2_O to CH_3_OH and then CH_3_OH:CH_2_Cl_2_ (50:50). Fraction eluted with CH_3_OH:H_2_O (75:25, 63.7 mg) were subjected to preparative HPLC (Symmetry C18, 7 μm, 19 × 150 mm, gradient H_2_O + 0.1% TFA:CH_3_CN + 0.1% TFA from 32% to 49% CH_3_CN + 0.1% TFA in 14 min and then from 49% to 100% in 1 min, flow: 13.6 mL/min, UV detection) to yield Tanjungide A (31.8 mg, retention time: 10.1 min) and Tanjungide B (1.6 mg, retention time: 9.2 min). Fraction eluted with CH_3_OH (100.7 mg), was also subjected to the same conditions of preparative HPLC to yield additional amounts of Tanjungides A and B.

Tanjungide A (**1**): Pale yellow amorphous solid. [α]^25^_D_ +114.9° (*c* 0.1, CH_3_OH); UV (CH_3_OH) λ_max_ 201, 236, 288 nm; IR (KBr) ν_max_ 3324, 1674, 1534, 1494, 1450, 1203, 1144, 803, 723 cm^−1^; (+)-HRESIMS *m/z* 518.9142 [M + H]^+^ (Calcd. for C_16_H_17_^79^Br_2_N_4_O_2_S_2_, 518.9154). ^1^H NMR (500 MHz) and ^13^C NMR (125 MHz) in DMSO-*d*_6_, see Table 1.

Tanjungide B (**2**): Pale yellow amorphous solid. [α]^25^_D_ +46.2° (*c* 0.1, CH_3_OH); UV (CH_3_OH) λ_max_ 201, 236, 291 nm; IR (KBr) ν_max_ 3296, 1676, 1571, 1447, 1203, 1141, 803, 726 cm^−1^; (+)-HRESIMS *m/z* 518.9142 [M + H]^+^ (Calcd. for C_16_H_17_^79^Br_2_N_4_O_2_S_2_, 518.9154). ^1^H NMR (500 MHz) and ^13^C NMR (125 MHz) in CD_3_OD, see Table 1.

### 3.4. Absolute Configuration of Cysteine Residues

Approximately 100 μL of Raney^®^-Nickel (50% slurry in H_2_O, excess) was added to Tanjungide A (1.02 mg) in methanol (1.2 mL), N_2_ was bubbled through the solution to remove O_2_. The resulting suspension was heated at 65 °C for 4 h under nitrogen and then the reaction mixture was left overnight at 23 °C. The disappearance of starting material was monitored by HPLC. The resulting solution was purified on a C18 SPE cartridge using methanol as eluent, yielding desthiotanjungide A. Desthiotanjungide A (200 μg) was dissolved in 6 N HCl (500 μL) and heated in a sealed glass vial at 110 °C overnight. The solvent was removed in a stream of dry N_2_. To the acid hydrolysate of desthiotanjungide A, a solution of l-FDAA (*N*α-(2,4-dinitro-5-fluorophenyl)-l-alaninamide, 700 μg) in acetone (160 μL), H_2_O (100 μL) and NaHCO_3_ 1 N (50 μL) were added. The vials were heated at 40 °C for 1 h, and the contents neutralized with 2N HCl (20 μL) after cooling to 23 °C. H_2_O (800 μL) was added to each reaction and the resulting mixture filtered and analyzed by RP18-HPLC-MS (Symmetry C18, 5 μm, 4.6 × 150 mm; linear gradient from 20% to 50% CH_3_CN (0.04% TFA) in H_2_O (0.04% TFA) over 20 min, flow rate: 0.8 mL/min). The amino acid standards (*R*)- and (*S*)-Ala (200 μg) were derivatized in a similar manner, and the retention times were compared with those of the alanines of desthiotanjungide A hydrolysate. The retention times of the authentic FDAA-Ala used as standards were as follows: (*S*)-Ala (13.9 min) and (*R*)-Ala (16.3 min). The hydrolysate of Tanjungide A contained: (*S*)-Ala (13.7 min).

### 3.5. Total Synthesis of Tanjungides A (**1**) and B (**2**)

#### 3.5.1. 5,6-Dibromo-1*H*-indole-3-carboxylic Acid (**4**)

To a stirred solution of methyl 5,6-dibromo-1*H*-indole-3-carboxylate (**3**) (12.5 g, 37.8 mmol) in CH_3_OH (124 mL) was added an aqueous solution of NaOH (188 mL, 2 M, 376 mmol). The suspension was refluxed for 2.5 h. After this time, the brown solution was cooled to 23 °C and the volatiles were evaporated. The aqueous phase was acidified with a 1 M solution of HCl until reached pH 2 and extracted with EtOAc. The combined organic layers were washed with brine, dried over Na_2_SO_4_, filtered and concentrated in vacuo to afford crude **4** (11.4 g, 95% yield) as a brown solid which was used in the next step without further purification. ^1^H NMR (300 MHz, CD_3_OD) δ_H_ ppm: 8.36 (s, 1H), 7.97 (s, 1H), 7.79 (s, 1H). ^13^C NMR (75 MHz, CD_3_OD) δ_C_ ppm: 166.9, 136.6, 134.0, 127.2, 125.2, 117.2, 116.7, 116.5, 107.5.

#### 3.5.2. 5,6-Dibromo-1*H*-indole (**5**)

5,6-Dibromo-1*H*-indole-3-carboxylic acid (**4**) (11.7 g, 36.7 mmol) was dissolved in pyridine (20.5 mL) and refluxed overnight. The solvent was concentrated in vacuo, the crude obtained was dissolved in CH_2_Cl_2_, precipitated with hexane and left at 5 °C overnight. The solid was filtered to yield crude **5** (9.6 g, 95% yield) which was used in the next step without further purification. ^1^H NMR (300 MHz, CDCl_3_) δ_H_ ppm: 7.90 (d, *J* = 1.1 Hz, 1H), 7.70 (t, *J* = 1.1 Hz, 1H), 7.21 (ddd, *J* = 3.6, 2.5, 1.2 Hz, 1H), 6.48 (ddt, *J* = 3.3, 2.2, 1.1 Hz, 1H). ^13^C NMR (75 MHz, CDCl_3_) δ_C_ ppm: 135.7, 129.0, 126.3, 125.1, 117.3, 115.9, 115.3, 102.6.

#### 3.5.3. 5,6-Dibromo-1*H*-indole-3-carbaldehyde (**6**)

To a stirred solution of DMF (46.8 mL) at 0 °C was dropwise added POCl_3_ (12.0 mL, 131.3 mmol). The mixture was further stirred for 5 min at 0 °C and a solution of 5,6-dibromo-1*H*-indole (**5**) (7.22 g, 26.3 mmol) in DMF (70 mL) was slowly added. The reaction mixture was stirred 1 h at 35 °C, 1 h at 65 °C, and was left to reach 23 °C. An aqueous solution of NaOH (72.3 mL, 2 N) was added at 0 °C and the reaction mixture was stirred 5 min at 110 °C, left to reach 23 °C, and then added over an ice-water bath in order to precipitate **6**. The reaction mixture was left overnight at 5 °C and filtered to obtain crude **6** (7.32 g, 92% yield) which was used in the next step without further purification. ^1^H NMR (300 MHz, DMSO-*d*_6_) δ_H_ ppm: 9.91 (d, *J* = 1.3 Hz, 1H), 8.51–8.20 (m, 2H), 7.91 (d, *J* = 1.3 Hz, 1H). ^13^C NMR (75 MHz, DMSO-*d*_6_) δ_C_ ppm: 185.9, 140.7, 137.4, 125.7, 125.4, 118.2, 118.1, 117.8, 117.4.

#### 3.5.4. *tert*-Butyl 5,6-dibromo-3-formyl-1*H*-indole-1-carboxylate (**7**)

To a stirred solution of 5,6-dibromo-1*H*-indole-3-carbaldehyde (**6**) (9.2 g, 30.4 mmol) in 1,4-dioxane (152 mL) was added successively di-*tert*-butyldicarbonate (7.9 g, 36.4 mmol) and DMAP (370 mg, 3.0 mmol). After stirring for 2 h at 23 °C, the mixture was quenched with H_2_O and extracted with EtOAc. The combined organic phases were washed thoroughly with H_2_O, dried over Na_2_SO_4_, filtered and concentrated in vacuo to afford **7** (10.5 g, 86% yield) as a slightly brown solid that was used in the next steps without further purification. ^1^H NMR (300 MHz, CDCl_3_) δ_H_ ppm: 10.03 (s, 1H), 8.54 (s, 1H), 8.47 (s, 1H), 8.18 (s, 1H), 1.71 (s, 9H). ^13^C NMR (75 MHz, CDCl_3_) δ_C_ ppm: 185.2, 148.3, 137.2, 135.5, 126.7, 126.6, 122.2, 120.9, 120.6, 120.4, 86.9, 28.2.

#### 3.5.5. (*Z*)-*tert*-Butyl 5,6-dibromo-3-(2-iodovinyl)-1*H*-indole-1-carboxylate (**8**)

To a suspension of iodomethyltriphpenylphosphonium iodide (14.6 g, 27.5 mmol) in anhydrous THF (157 mL) was added a solution of sodium bis(trimethylsilyl)amide (NaHMDS) (27.5 mL, 1.0 M in THF, 27.5 mmol) dropwise at 23 °C. After stirring for 2 min, the yellow mixture was cooled to −78 °C and a solution of **7** (7.9 g, 19.6 mmol) in anhydrous THF (98 mL) was then added. The reaction mixture was stirred at −78 °C for 2 h, at 23 °C for 5 min, diluted with hexane, and filtered through a plug of Celite^®^. The plug was rinsed with hexane, the combined filtrates were evaporated under reduced pressure affording **8** (8.62 g, 83% yield) as a brown solid that was used in the next steps without further purification. ^1^H NMR (300 MHz, CDCl_3_) δ_H_ ppm: 8.56 (s, 1H), 8.48 (s, 1H), 7.80 (s, 1H), 7.43 (d, 1H, *J* = 8.7 Hz), 6.67 (d, 1H, *J* = 8.7 Hz), 1.69 (s, 9H). ^13^C NMR (75 MHz, CDCl_3_) δ_C_ ppm: 149.1, 135.5, 130.6, 128.5, 125.0, 124.0, 123.1, 120.5, 118.8, 116.4, 85.4, 81.0, 28.3.

#### 3.5.6. (*R*)-Allyl (1-amino-1-oxo-3-(tritylthio)propan-2-yl)carbamate (**9**)

To a stirred solution of *S*-trityl-l-cysteine amide (500 mg, 1.38 mmol) in a mixture THF:H_2_O (2.5 mL:1.25 mL) at 0 °C was added solid NaHCO_3_ (232 mg, 2.76 mmol) followed by allyloxycarbonyl chloride (0.14 mL, 1.65 mmol). After stirring for 2 h at 0 °C, the mixture was quenched by slow addition of a 2 M solution of HCl until reached pH 2 and extracted with CH_2_Cl_2_. The combined organic layers were dried over Na_2_SO_4_, filtered and the solvent was removed under reduced pressure to afford **9** as a white solid (616 mg, 100% yield) that was used without further purification. ^1^H NMR (300 MHz, CDCl_3_) δ_H_ ppm: 7.43 (m, 5H), 7.33–7.19 (m, 10H), 5.88 (m, 1H), 5.81 (br s, 1H), 5.33 (br s, 1H), 5.29 (d, 1H, 16.0 Hz), 5.22 (d, 1H, *J* = 10.5 Hz), 5.06 (d, 1H, *J* = 7.2 Hz), 4.52 (dd, 2H, 5.7, *J* = 1.2 Hz), 3.87 (m, 1H), 2.76 (dd, 1H, *J* = 13.2, 7.2 Hz), 2.57 (dd, 1H, *J* = 13.2, 5.1 Hz). ^13^C NMR (75 MHz, CDCl_3_) δ_C_ ppm: 172.4, 156.0, 144.5, 132.5, 129.8, 128.3, 127.2, 118.2, 67.6, 66.3, 53.7, 33.9.

#### 3.5.7. (*R*,*Z*)-*tert*-Butyl 3-(2-(2-(((allyloxy)carbonyl)amino)-3-(tritylthio)propanamido)vinyl)-5,6-dibromo-1*H*-indole-1-carboxylate (*Z*-**10**)

A Schlenk tube was charged with copper(I) iodide (39 mg, 0.20 mmol), cesium carbonate (667 mg, 2.05 mmol) and *N*-alloc-*S*-trityl-l-cysteine-amide (**9**) (455 mg, 1.02 mmol), evacuated and filled with N_2_. *N*,*N*′-dimethylethylenediamine (44 μL, 0.41 mmol), vinyl iodide **8** (360 mg, 0.68 mmol) and dry THF (4 mL) were added. The Schlenk tube was sealed, heated at 60 °C for 18 h and cooled to 23 °C. The resultant mixture was diluted with EtOAc and quenched with H_2_O. The organic layer was washed with H_2_O and dried over Na_2_SO_4_. The solvent was removed under reduced pressure and the residue was purified by flash chromatography on silica gel (hexane:EtOAc, 4:1) to yield successively pure (*Z*)-**10** (285 mg, 50% yield) and (*E*)-**10** (77 mg, 13% yield) as brown solids. ^1^H NMR (300 MHz, CDCl_3_) δ_H_ ppm: 8.46 (s, 1H), 8.21 (d, 1H, *J* = 8.1 Hz), 7.73 (s, 1H), 7.61 (s, 1H), 7.34 (m, 5H), 7.22 (m, 10H), 6.96 (dd, 1H, *J* = 11.1, 9.3 Hz), 5.78 (m, 1H), 5.68 (d, 1H, *J* = 9.6 Hz), 5.21 (d, 1H, *J* = 17.1 Hz), 5.14 (d, 1H, *J* = 10.5 Hz), 4.90 (d, 1H, *J* = 7.2 Hz), 4.40 (m, 2H), 3.83 (m, 1H), 2.83 (dd, 1H, *J* = 13.2, 6.9 Hz), 2.58 (dd, 1H, *J* = 13.2, 5.7 Hz), 1.68 (s, 9H). ^13^C NMR (75 MHz, CDCl_3_) δ_C_ ppm: 168.1, 156.4, 149.0, 144.4, 134.7, 132.4, 130.4, 129.7, 128.3, 127.1, 124.7, 123.8, 123.3, 120.8, 120.5, 118.8, 118.2, 114.3, 100.2, 85.2, 67.7, 66.5, 54.1, 32.9, 28.3.

#### 3.5.8. (*R*,*E*)-*tert*-Butyl 3-(2-(2-(((allyloxy)carbonyl)amino)-3-(tritylthio)propanamido)vinyl)-5,6-dibromo-1*H*-indole-1-carboxylate (*E*-**10**)

A Schlenk tube was charged with copper(I) iodide (11 mg, 0.06 mmol), potassium carbonate (78 mg, 0.57 mmol) and *N*-alloc-*S*-trityl-l-cysteine-amide (**9**) (127 mg, 0.284 mmol), evacuated and filled with N_2_. *N*,*N*′-dimethylethylenediamine (12 μL, 0.11 mmol), vinyl iodide **8** (100 mg, 0.19 mmol) and dry THF (5 mL) were added. The Schlenk tube was sealed, heated at 80 °C for 18 h and cooled to 23 °C. The resultant mixture was diluted with EtOAc and quenched with H_2_O. The organic layer was washed with H_2_O and dried over Na_2_SO_4_. The solvent was removed under reduced pressure and the residue was purified by flash chromatography on silica gel (hexane:EtOAc, 4:1) to yield pure (*E*)-**10** (96 mg, 60% yield) and (*Z*)-**10** (22 mg, 14% yield) as brown solids. ^1^H NMR (300 MHz, CDCl_3_) δ_H_ ppm: 8.50 (s, 1H), 7.87 (s, 1H), 7.83 (d, 1H, *J* = 8.1 Hz), 7.50 (s, 1H), 7.45 (m, 5H), 7.35–7.21 (m, 11H), 6.10 (d, 1H, *J* = 15.3 Hz), 5.90 (m, 1H), 5.32 (d, 1H, *J* = 17.1 Hz), 5.25 (d, 1H, *J* = 10.2 Hz), 4.99 (d, 1H, *J* = 6.9 Hz), 4.56 (dd, 2H, *J* = 5.7, 2.7 Hz), 3.87 (q, 1H, *J* = 6.6 Hz), 2.85 (dd, 1H, *J* = 13.2, 6.9 Hz), 2.62 (dd, 1H, *J* = 13.2, 5.7 Hz), 1.66 (s, 9H). ^13^C NMR (75 MHz, CDCl_3_) δ_C_ ppm: 167.7, 156.2, 149.0, 144.5, 135.4, 132.4, 129.8, 129.4, 128.4, 127.2, 124.0, 123.5, 123.0, 120.6, 120.5, 118.8, 118.5, 116.2, 104.3, 85.0, 67.8, 66.6, 54.3, 33.6, 28.3.

#### 3.5.9. (*R*,*Z*)-*tert*-Butyl 3-(2-(2-amino-3-(tritylthio)propanamido)vinyl)-5,6-dibromo-1*H*-indole-1-carboxylate (*Z*-**11**)

To a stirred solution of enamide (*Z*)-**10** (1.53 g, 1.81 mmol) in CH_2_Cl_2_ (46 mL) was added successively PhSiH_3_ (4.46 mL, 36.2 mmol) and Pd(PPh_3_)_4_ (313 mg, 0.27 mmol). After stirring for 30 min at 23 °C all volatiles were evaporated and the crude mixture was purified by flash chromatography on silica gel (hexane:EtOAc, 2:1) to afford pure (*Z*)-**11** (1.17 g, 85% yield) as a brown solid. ^1^H NMR (300 MHz, CDCl_3_) δ_H_ ppm: 9.57 (d, 1H, *J* = 12.0 Hz), 8.48 (s, 1H), 7.77 (s, 1H), 7.59 (s, 1H), 7.43 (m, 5H), 7.25 (m, 10H), 6.92 (dd, 1H, *J* = 11.0, 8.7 Hz), 5.69 (d, 1H, *J* = 9.3 Hz), 3.12 (m, 1H), 2.77 (dd, 1H, *J* = 9.0, 4.5 Hz), 2.66 (dd, 1H, *J* = 12.9, 8.4 Hz), 1.66 (s, 9H). ^13^C NMR (75 MHz, CDCl_3_) δ_C_ ppm: 170.5, 148.8, 144.4, 129.5, 128.0, 127.9, 126.9, 123.9, 123.8, 122.6, 122.5, 120.6, 120.2, 118.5, 114.8, 99.2, 84.7, 67.1, 53.7, 36.8, 28.1.

#### 3.5.10. (*R*,*E*)-*tert*-Butyl 3-(2-(2-amino-3-(tritylthio)propanamido)vinyl)-5,6-dibromo-1*H*-indole-1-carboxylate (*E*-**11**)

To a stirred solution of enamide (*E*)-**10** (265 mg, 0.31 mmol) in CH_2_Cl_2_ (8 mL) was added successively PhSiH_3_ (0.77 mL, 6.3 mmol) and Pd(PPh_3_)_4_ (54 mg, 0.05 mmol). After stirring for 25 min at 23 °C all volatiles were evaporated and the crude mixture was purified by flash chromatography on silica gel (hexane:EtOAc, 2:1) to obtain (*E*)-**11** (170 mg, 72% yield) as a brown solid. ^1^H NMR (300 MHz, CDCl_3_) δ_H_ ppm: 9.14 (d, 1H, *J* = 11.4 Hz), 8.48 (s, 1H), 7.86 (s, 1H), 7.45 (m, 6H), 7.37–7.20 (m, 11H), 6.10 (d, 1H, *J* = 15.3 Hz), 3.14 (m, 1H), 2.78 (m, 1H), 2.67 (m, 1H), 1.68 (s, 9H). ^13^C NMR (75 MHz, CDCl_3_) δ_C_ ppm: 170.6, 149.0, 144.7, 135.4, 132.3, 129.8, 129.5, 128.2, 127.1, 124.0, 123.2, 120.5, 120.4, 118.7, 116.5, 103.6, 84.9, 67.4, 53.9, 37.2, 28.3.

#### 3.5.11. *tert*-Butyl 5,6-dibromo-3-((6*R*,9*R*,*Z*)-2,2-dimethyl-4,7,10-trioxo-6,9-bis((tritylthio)methyl)-3-oxa-5,8,11-triazatridec-12-en-13-yl)-1*H*-indole-1-carboxylate (*Z*-**12**)

To a stirred solution of amine (*Z*)-**11** (3.75 g, 4.92 mmol) and *N*-boc-l-(*S*-trityl)-cys (2.73 g, 5.91 mmol) in anhydrous CH_2_Cl_2_:DMF (4:1, 59 mL:15 mL) at 0 °C, were added diisopropylethylamine (DIPEA) (1.28 mL, 7.4 mmol), 1-hydroxybenzotriazole (HOBt) (730 mg, 5.41 mmol) and *N*,*N*,*N*′,*N*′-tetramethyl-*O*-(7-azabenzotriazol-1-yl)uronium hexafluorophosphate (HATU) (2.05 g, 5.41 mmol). After 30 min the cold bath was removed and the reaction mixture was stirred at 23 °C for 2 h, quenched with a saturated aqueous solution of NH_4_Cl, poured into H_2_O and extracted with CH_2_Cl_2_. The combined organic phases were dried over anhydrous Na_2_SO_4_, filtered and concentrated. The residue was purified by flash chromatography on silica gel (hexane:EtOAc, 4:1) to give pure (*Z*)-**12** (4.97 g, 84% yield) as a yellow solid. ^1^H NMR (300 MHz, CDCl_3_) δ_H_ ppm: 8.44 (d, 1H, *J* = 11.3 Hz), 8.41 (s, 1H), 7.72 (s, 1H), 7.38 (m, 10H), 7.30 (s, 1H), 7.25 (m, 20H), 6.89 (dd, 1H, *J* = 11.4, 9.0 Hz), 6.02 (br s, 1H), 5.60 (d, 1H, *J* = 9.6 Hz), 5.09 (br s, 1H), 4.16 (m, 1H), 3.92 (m, 1H), 2.88 (m, 1H), 2.55 (dd, 1H, *J* = 12.9, 4.5 Hz), 2.49 (m, 1H), 2.37 (dd, 1H, *J* = 12.6, 6.3 Hz), 1.69 (s, 9H), 1.27 (s, 9H). ^13^C NMR (75 MHz, CDCl_3_) δ_C_ ppm: 171.3, 167.7, 155.4, 149.3, 144.5, 144.3, 134.6, 131.0, 129.7, 129.6, 128.3, 128.2, 127.2, 127.1, 126.6, 125.2, 123.6, 122.9, 120.4, 118.5, 114.1, 100.3, 85.1, 80.6, 67.6, 67.1, 53.5, 52.4, 34.1, 32.1, 28.4, 28.3.

#### 3.5.12. *tert*-Butyl 5,6-dibromo-3-((6*R*,9*R*,*E*)-2,2-dimethyl-4,7,10-trioxo-6,9-bis((tritylthio)methyl)-3-oxa-5,8,11-triazatridec-12-en-13-yl)-1*H*-indole-1-carboxylate (*E*-**12**)

To a stirred solution of amine (*E*)-**11** (170 mg, 0.22 mmol) and *N*-boc-l-(*S*-trityl)-cys (124 mg, 0.27 mmol) in anhydrous CH_2_Cl_2_:DMF (4:1, 2.6 mL:0.7 mL) at 0 °C, were added DIPEA (58 μL, 0.33 mmol), HOBt (33 mg, 0.24 mmol) and HATU (93 mg, 0.24 mmol). After 20 min the cold bath was removed and the reaction mixture was stirred at 23 °C for 2.4 h, quenched with a saturated aqueous solution of NH_4_Cl, poured into H_2_O and extracted with CH_2_Cl_2_. The combined organic phases were dried over anhydrous Na_2_SO_4_, filtered and concentrated. The residue was purified by flash chromatography on silica gel (hexane:EtOAc, 3:1) to afford (*E*)-**12** (184 mg, 73% yield) as a yellow solid. ^1^H NMR (300 MHz, CDCl_3_) δ_H_ ppm: 8.86 (d, 1H, *J* = 10.5 Hz), 8.49 (s, 1H), 7.81 (s, 1H), 7.50–7.36 (m, 11H), 7.34–7.16 (m, 21H), 6.34 (d, 1H, *J* = 8.1 Hz), 6.25 (d, 1H, *J* = 14.4 Hz), 4.87 (br s, 1H), 4.40 (m, 1H), 3.65 (m,1H), 3.15 (dd, 1H, *J* = 12.3, 5.4 Hz), 2.70 (dd, 1H, *J* = 13.2, 4.5 Hz), 2.56 (m, 1H), 2.38 (dd, 1H, *J* = 12.9, 4.5 Hz), 1.66 (s, 9H), 1.26 (s, 9H).

#### 3.5.13. *tert*-Butyl 5,6-dibromo-3-((*Z*)-2-((4*R*,7*R*)-7-((tert-butoxycarbonyl)amino)-6-oxo-1,2,5-dithiazocane-4-carboxamido)vinyl)-1*H*-indole-1-carboxylate (*Z*-**13**)

Over a solution of I_2_ (897 mg; 3.54 mmol) in CH_2_Cl_2_:CH_3_OH (10:1; 1416 mL) a solution of (*Z*)-**12** (610 mg; 0.50 mmol) in CH_2_Cl_2_ (100 mL) was added at 23 °C. The reaction mixture was stirred over 40 min, and a 5% aqueous solution of Na_2_S_2_O_4_ was added. The aqueous layer was extracted with CH_2_Cl_2_, the combined organic layers were dried over Na_2_SO_4_, filtered, and concentrated under vacuum. The residue obtained was purified by flash chromatography on silica gel (hexane:EtOAc, 3:1) to give pure (*Z*)-**13** (302 mg, 83% yield) as a white solid. ^1^H NMR (300 MHz, CDCl_3_) δ_H_ ppm: (mixture of conformers, signals for major conformer) 9.90 (d, 1H, *J* = 11.1 Hz), 8.45 (s, 1H), 7.78 (s, 1H), 7.62 (s, 1H), 7.08 (t, 1H, *J* = 9.9 Hz), 5.81 (d, 1H, *J* = 9.9 Hz), 5.65 (br s, 1H), 5.05 (br s, 1H), 4.93 (m, 1H), 4.48 (m, 1H), 3.67 (m, 1H), 3.37–2.87 (m, 3H), 1.70 (s, 9H), 1.25 (s, 9H). ^13^C NMR (75 MHz, CDCl_3_) δ_C_ ppm: (signals for major conformer) 172.3, 167.3, 154.8, 149.1, 130.3, 125.4, 125.1, 124.1, 123.7, 122.3, 120.7, 118.9, 114.0, 101.9, 85.2, 80.6, 57.1, 54.5, 43.2, 41.8, 28.6, 28.3.

#### 3.5.14. *tert*-Butyl 5,6-dibromo-3-((*E*)-2-((4*R*,7*R*)-7-((tert-butoxycarbonyl)amino)-6-oxo-1,2,5-dithiazocane-4-carboxamido)vinyl)-1*H*-indole-1-carboxylate (*E*-**13**)

Over a solution of I_2_ (265 mg; 1.04 mmol) in CH_2_Cl_2_:CH_3_OH (10:1; 416 mL) a solution of (*E*)-**12** (180 mg; 0.15 mmol) in CH_2_Cl_2_ (30 mL) was added at 23 °C. The reaction mixture was stirred over 40 min, and a 5% aqueous solution of Na_2_S_2_O_4_ was added. The aqueous layer was extracted with CH_2_Cl_2_, the combined organic layers were dried over Na_2_SO_4_, filtered, and concentrated under vacuum. The residue obtained was purified by flash chromatography on silica gel (hexane:EtOAc, 6:4) to obtain pure (*E*)-**13** (65 mg, 62% yield) as a white solid. ^1^H NMR (300 MHz, CDCl_3_) δ_H_ ppm: (mixture of conformers, signals for major conformer) 9.40 (d, 1H, *J* = 10.5 Hz), 8.32 (s, 1H), 7.70 (s, 1H), 7.44 (s, 1H), 7.34 (m, 1H), 6.92 (d, 1H, *J* = 11.1 Hz), 6.21 (d, 1H, *J* = 15.0 Hz), 5.10 (br s, 1H), 5.01 (m, 1H), 4.80 (m, 1H), 3.78 (m, 1H), 3.45 (m, 1H), 3.05 (m, 1H), 2.87 (m, 1H), 1.63 (s, 9H), 1.42 (s, 9H). ^13^C NMR (75 MHz, CDCl_3_) δ_C_ ppm: (signals for major conformer) 173.3, 166.7, 155.6, 148.9, 135.1, 129.2, 123.7, 122.8, 120.4, 120.2, 118.5, 115.9, 115.7, 105.5, 85.0, 81.1, 53.8, 48.8, 42.8, 36.9, 28.6, 28.3.

#### 3.5.15. Tanjungide A (**1**)

Over a solution of (*Z*)-**13** (310 mg; 0.43 mmol) in CH_2_Cl_2_ (9.3 mL) was dropwise added TFA (2.8 mL) at 0 °C. The reaction mixture was stirred at 0 °C for 8 h and a saturated aqueous solution of NaHCO_3_ was added until pH 8. The organic layer was extracted with EtOAc (×3), the combined organic layers were dried over Na_2_SO_4_, filtered and concentrated under vacuum. The residue obtained was purified by flash chromatography on silica gel (CH_2_Cl_2_:CH_3_OH, 30:1) to yield pure Tanjungide A (**1**) (135 mg, 60% yield) as a pale yellow solid and exhibited physical and spectroscopic characteristics (^1^H, ^13^C NMR and MS) equivalent to those reported in 3.3.

#### 3.5.16. Tanjungide B (**2**)

Over a solution of (*E*)-**13** (52 mg; 0.07 mmol) in CH_2_Cl_2_ (1.6 mL) was dropwise added TFA (0.47 mL) at 0 °C. The reaction mixture was stirred at 0 °C for 3 h and a saturated aqueous solution of NaHCO_3_ was added until pH 8. The organic layer was extracted with EtOAc (×3), the combined organic layers were dried over Na_2_SO_4_, filtered and concentrated under vacuum. The residue obtained was purified by flash chromatography on silica gel (CH_2_Cl_2_:CH_3_OH, 20:1) to give pure Tanjungide B (**2**) (16 mg, 45% yield) as a pale yellow solid and exhibited physical and spectroscopic characteristics (^1^H, ^13^C NMR and MS) equivalent to those reported in section 3.3.

### 3.6. Biological Activity

A549 (ATCC CCL-185), lung carcinoma; HT29 (ATCC HTB-38), colorectal carcinoma and MDA-MB-231 (ATCC HTB-26), breast adenocarcinoma cell lines were obtained from the ATCC. Cell lines were maintained in RPMI medium supplemented with 10% fetal calf serum (FCS), 2 mM l-glutamine and 100 U/mL penicillin and streptomycin, at 37 °C and 5% CO_2_. Triplicate cultures were incubated for 72 h in the presence or absence of test compounds (at ten concentrations ranging from 10 to 0.0026 μg/mL). For quantitative estimation of cytotoxicity, the colorimetric sulforhodamine B (SRB) method was used [14]. Briefly, cells were washed twice with PBS, fixed for 15 min in 1% glutaraldehyde solution, rinsed twice in PBS, and stained in 0.4% SRB solution for 30 min at room temperature. Cells were then rinsed several times with 1% acetic acid solution and air-dried. Sulforhodamine B was then extracted in 10 mM trizma base solution and the absorbance measured at 490 nm. Results are expressed as GI_50_, the concentration that causes 50% inhibition in cell growth after correction for cell count at the start of the experiment (NCI algorithm). Doxorubicin and DMSO (solvent) were used as the positive and negative controls in this assay. Prism 3.03 from GraphPad was used for the statistical analysis of the cell growth inhibition results.

## 4. Conclusions

In summary, we have isolated, determined the structure and completed the first total synthesis of Tanjungides A and B, two new bromoindole enamides with interesting cytotoxic properties from the tunicate *Diazona* cf *formosa*. The total synthesis confirmed the structural assignment and provides rapid access to these new natural products and related analogues for biological evaluation and drug development.

## Supplementary Files

Supplementary File 1 Supplementary Information (PDF, 2377 KB)
