# Supplementary material for: Tanjungides A and B: New Antitumoral Bromoindole Derived Compounds from Diazona cf formosa. Isolation and Total Synthesis
Source: Mar Drugs. 2014 Feb 21;12(2):1116–30. doi: 10.3390/md12021116 (PMC3944533; doi:10.3390/md12021116)
Supplement: Supplementary File 1 — Supplementary Information (PDF, 2377 KB) [file marinedrugs-12-01116-s001.pdf]

Supplementary Information

Figure S1. ^{1}H NMR spectrum of Tanjungide A (500 MHz, DMSO-d_6).
Figure S2. ^{13}C NMR spectrum of Tanjungide A (500 MHz, DMSO-d_6).
Figure S3. *g*-COSY spectrum of Tanjungide A.
Figure S4. *g*-HSQC spectrum of Tanjungide A.
Figure S5. *g*-HMBC spectrum of Tanjungide A.
Figure S6. ROESY spectrum of Tanjungide A.
Figure S7. ^{1}H NMR spectrum of Tanjungide B (500 MHz, CD_3OD).
Figure S8. ^{13}C NMR spectrum of Tanjungide B (125 MHz, CD_3OD).
Figure S9. *g*-COSY spectrum Tanjungide B.
Figure S10. *g*-HSQC spectrum of Tanjungide B.
Figure S11. *g*-HMBC spectrum of Tanjungide B.
Figure S12. ROESY spectrum of Tanjungide B.
Figure S13. ^{1}H NMR spectrum of 5,6-dibromo-1H-indole-3-carboxylic acid (**4**) (300 MHz, CD_3OD).
Figure S14. ^{13}C NMR spectrum of 5,6-dibromo-1H-indole-3-carboxylic acid (**4**) (75 MHz, CD_3OD).
Figure S15. ^{1}H NMR spectrum of 5,6-dibromo-1*H*-indole (**5**) (300 MHz, $CDCl_3$).
Figure S16. ^{13}C NMR spectrum of 5,6-dibromo-1*H*-indole (**5**) (75 MHz, $CDCl_3$).
Figure S17. ^{1}H NMR spectrum of 5,6-dibromo-1*H*-indole-3-carbaldehyde (**6**) (300 MHz, DMSO-d_6).
Figure S18. ^{13}C NMR spectrum of 5,6-dibromo-1*H*-indole-3-carbaldehyde (**6**) (75 MHz, DMSO-d_6).
Figure S19. ^{1}H NMR spectrum of *tert*-butyl 5,6-dibromo-3-formyl-1*H*-indole-1-carboxylate (**7**) (300 MHz, $CDCl_3$).
Figure S20. ^{13}C NMR spectrum of *tert*-butyl 5,6-dibromo-3-formyl-1*H*-indole-1-carboxylate (**7**) (75 MHz, $CDCl_3$).
Figure S21. ^{1}H NMR spectrum of (*Z*)-*tert*-butyl 5,6-dibromo-3-(2-iodovinyl)-1*H*-indole-1-carboxylate (**8**) (300 MHz, $CDCl_3$).
Figure S22. ^{13}C NMR spectrum of (*Z*)-*tert*-butyl 5,6-dibromo-3-(2-iodovinyl)-1*H*-indole-1-carboxylate (**8**) (75 MHz, $CDCl_3$).
Figure S23. ^{1}H NMR spectrum of (*R*,*Z*)-*tert*-butyl 3-(2-(2-(((allyloxy)carbonyl)amino)-3-(tritylthio)propanamido)vinyl)-5,6-dibromo-1*H*-indole-1-carboxylate (*Z*-**10**) (300 MHz, $CDCl_3$).
Figure S24. ^{13}C NMR spectrum of (*R*,*Z*)-*tert*-butyl 3-(2-(2-(((allyloxy)carbonyl)amino)-3-(tritylthio)propanamido)vinyl)-5,6-dibromo-1*H*-indole-1-carboxylate (*Z*-**10**) (75 MHz, $CDCl_3$).

Figure S25. ^{1}H NMR spectrum of (*R*,*E*)-*tert*-butyl 3-(2-(2-(((allyloxy)carbonyl)amino)-3-(tritylthio)propanamido)vinyl)-5,6-dibromo-1*H*-indole-1-carboxylate (*E*-**10**) (300 MHz, $CDCl_3$).

Figure S26. ^{13}C NMR spectrum of (*R*,*E*)-*tert*-butyl 3-(2-(2-(((allyloxy)carbonyl)amino)-3-(tritylthio)propanamido)vinyl)-5,6-dibromo-1*H*-indole-1-carboxylate (*E*-**10**) (75 MHz, $CDCl_3$).

Figure S27. ^{1}H NMR spectrum of (*R*,*Z*)-*tert*-butyl 3-(2-(2-amino-3-(tritylthio)propanamido)vinyl)-5,6-dibromo-1*H*-indole-1-carboxylate (*Z*-**11**) (300 MHz, $CDCl_3$).

Figure S28. ^{13}C NMR spectrum of (*R*,*Z*)-*tert*-butyl 3-(2-(2-amino-3-(tritylthio)propanamido)vinyl)-5,6-dibromo-1*H*-indole-1-carboxylate (*Z*-**11**) (75 MHz, $CDCl_3$).

Figure S29. ^{1}H NMR spectrum of (*R*,*E*)-*tert*-butyl 3-(2-(2-amino-3-(tritylthio)propanamido)vinyl)-5,6-dibromo-1*H*-indole-1-carboxylate (*E*-**11**) (300 MHz, $CDCl_3$).

Figure S30. ^{13}C NMR spectrum of (*R*,*E*)-*tert*-butyl 3-(2-(2-amino-3-(tritylthio)propanamido)vinyl)-5,6-dibromo-1*H*-indole-1-carboxylate (*E*-**11**) (75 MHz, $CDCl_3$).

Figure S31. ^{1}H NMR spectrum of *tert*-butyl 5,6-dibromo-3-((6*R*,9*R*,*Z*)-2,2-dimethyl-4,7,10-trioxo-6,9-bis((tritylthio)methyl)-3-oxa-5,8,11-triazatridec-12-en-13-yl)-1*H*-indole-1-carboxylate (*Z*-**12**) (300 MHz, $CDCl_3$).

Figure S32. ^{13}C NMR spectrum of *tert*-butyl 5,6-dibromo-3-((6*R*,9*R*,*Z*)-2,2-dimethyl-4,7,10-trioxo-6,9-bis((tritylthio)methyl)-3-oxa-5,8,11-triazatridec-12-en-13-yl)-1*H*-indole-1-carboxylate (*Z*-**12**) (75 MHz, $CDCl_3$).

Figure S33. ^{1}H NMR spectrum of *tert*-butyl 5,6-dibromo-3-((6*R*,9*R*,*E*)-2,2-dimethyl-4,7,10-trioxo-6,9-bis((tritylthio)methyl)-3-oxa-5,8,11-triazatridec-12-en-13-yl)-1*H*-indole-1-carboxylate (*E*-**12**) (300 MHz, $CDCl_3$).

Figure S34. ^{1}H NMR spectrum of *tert*-butyl 5,6-dibromo-3-((*Z*)-2-((4*R*,7*R*)-7-((tert-butoxycarbonyl)amino)-6-oxo-1,2,5-dithiazocane-4-carboxamido)vinyl)-1*H*-indole-1-carboxylate (*Z*-**13**) (300 MHz, $CDCl_3$).

Figure S35. ^{13}C NMR spectrum of *tert*-butyl 5,6-dibromo-3-((*Z*)-2-((4*R*,7*R*)-7-((tert-butoxycarbonyl)amino)-6-oxo-1,2,5-dithiazocane-4-carboxamido)vinyl)-1*H*-indole-1-carboxylate (*Z*-**13**) (75 MHz, $CDCl_3$).

Figure S36. ^{1}H NMR spectrum of *tert*-butyl 5,6-dibromo-3-((*E*)-2-((4*R*,7*R*)-7-((tert-butoxycarbonyl)amino)-6-oxo-1,2,5-dithiazocane-4-carboxamido)vinyl)-1*H*-indole-1-carboxylate (*E*-**13**) (300 MHz, $CDCl_3$).

Figure S37. ^{13}C NMR spectrum of *tert*-butyl 5,6-dibromo-3-((*E*)-2-((4*R*,7*R*)-7-((tert-butoxycarbonyl)amino)-6-oxo-1,2,5-dithiazocane-4-carboxamido)vinyl)-1*H*-indole-1-carboxylate (*E*-**13**) (75 MHz, $CDCl_3$).

Figure S38. ^{1}H NMR spectra of synthetic *vs.* natural Tanjungide A.

Figure S39. Marfey ́s reaction. Desthiotanjungide A.

Figure S1. ^{1}H NMR spectrum of Tanjungide A (500 MHz, DMSO-d_6).

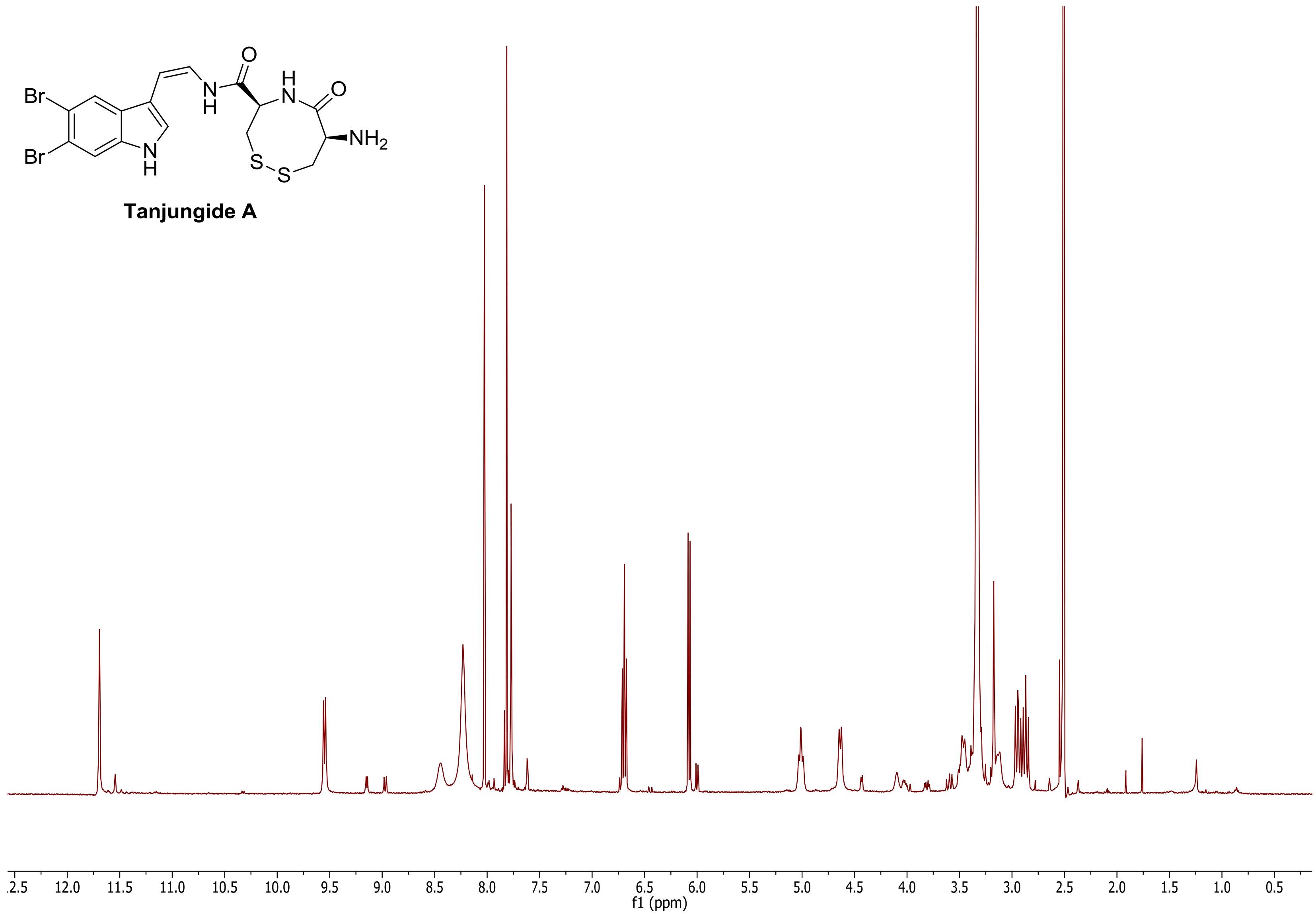

Figure S2. ^{13}C NMR spectrum of Tanjungide A (500 MHz, DMSO-d_6).

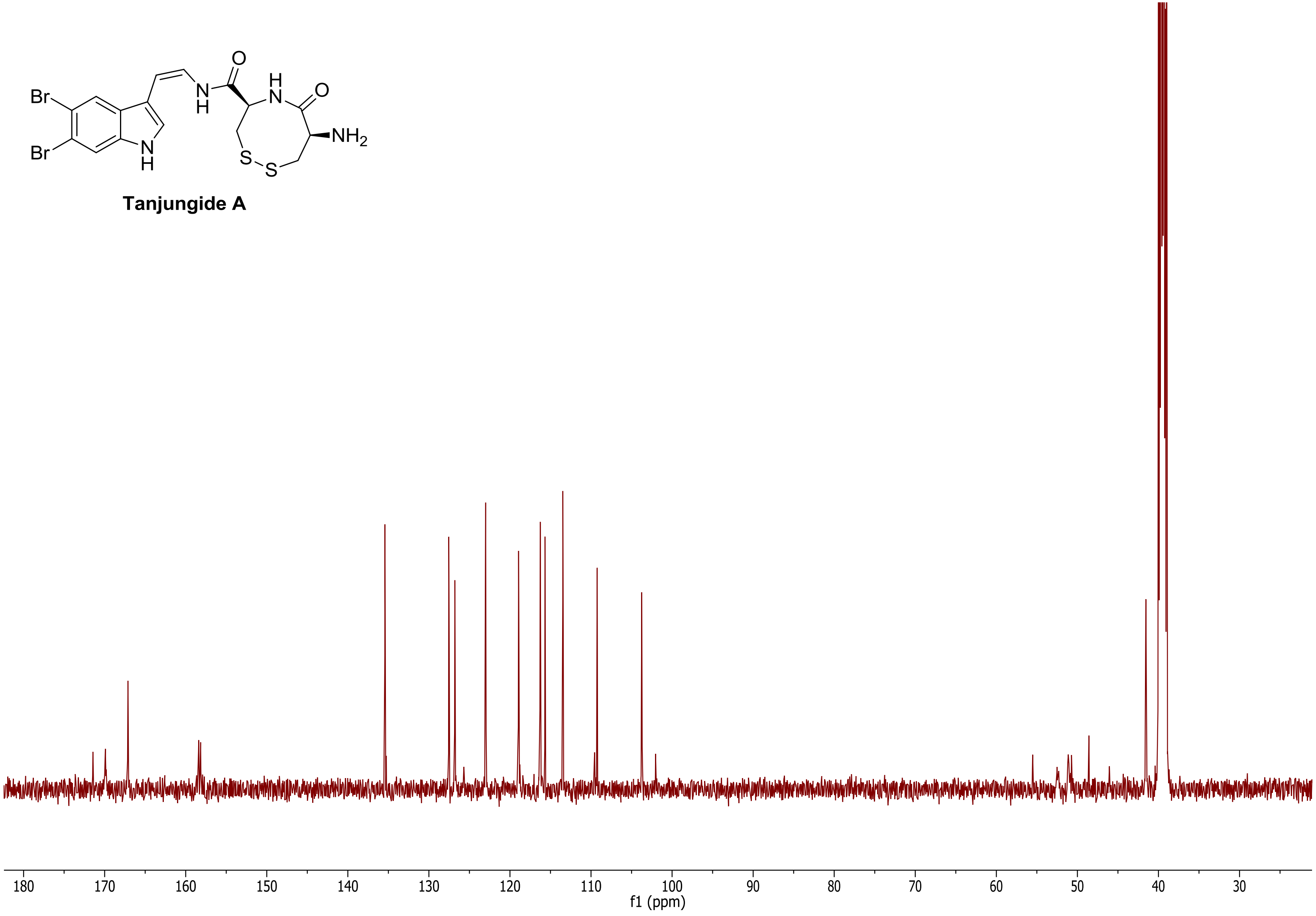

Figure S3. *g*-COSY spectrum of Tanjungide A.

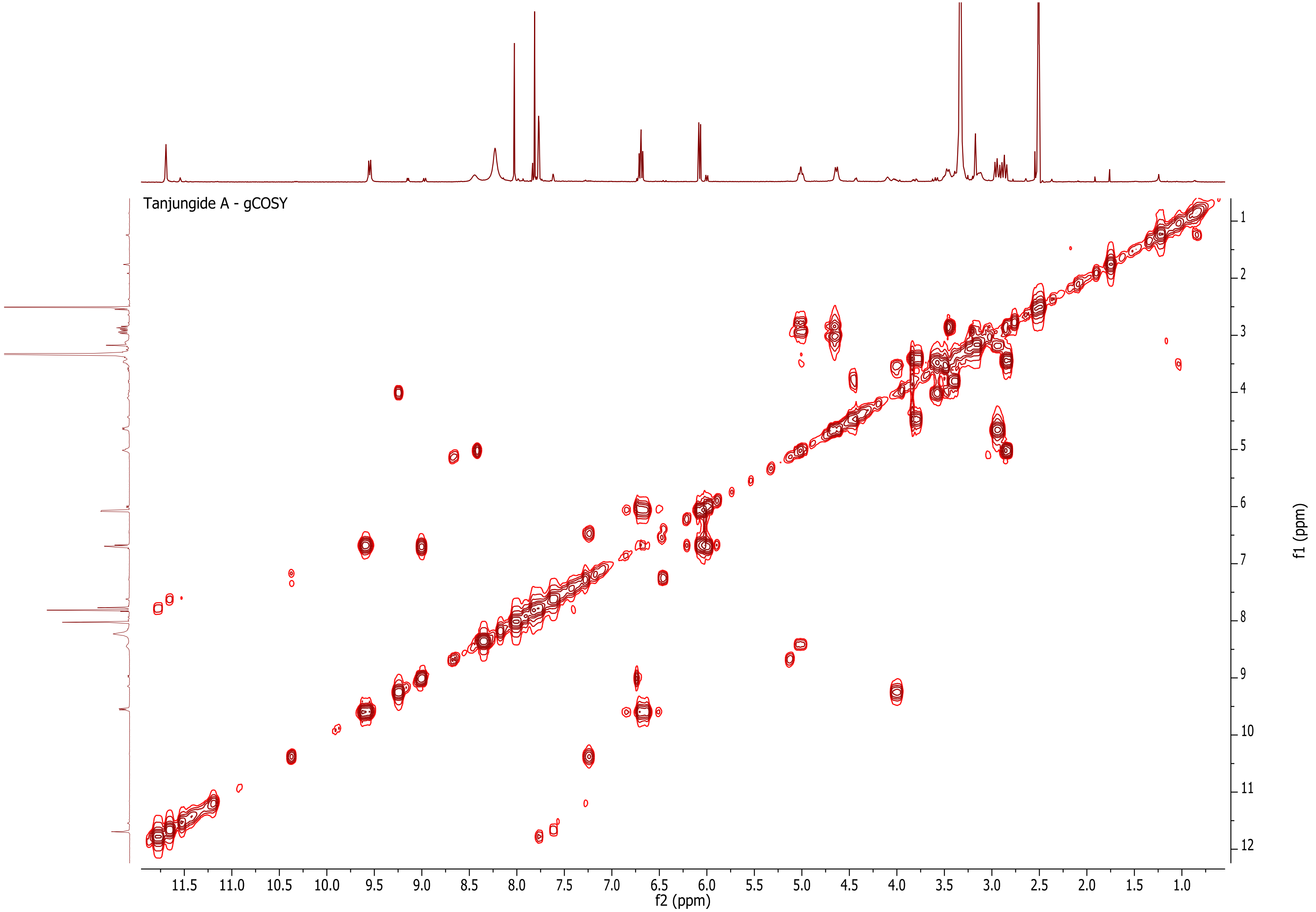

Figure S4. *g*-HSQC spectrum of Tanjungide A.

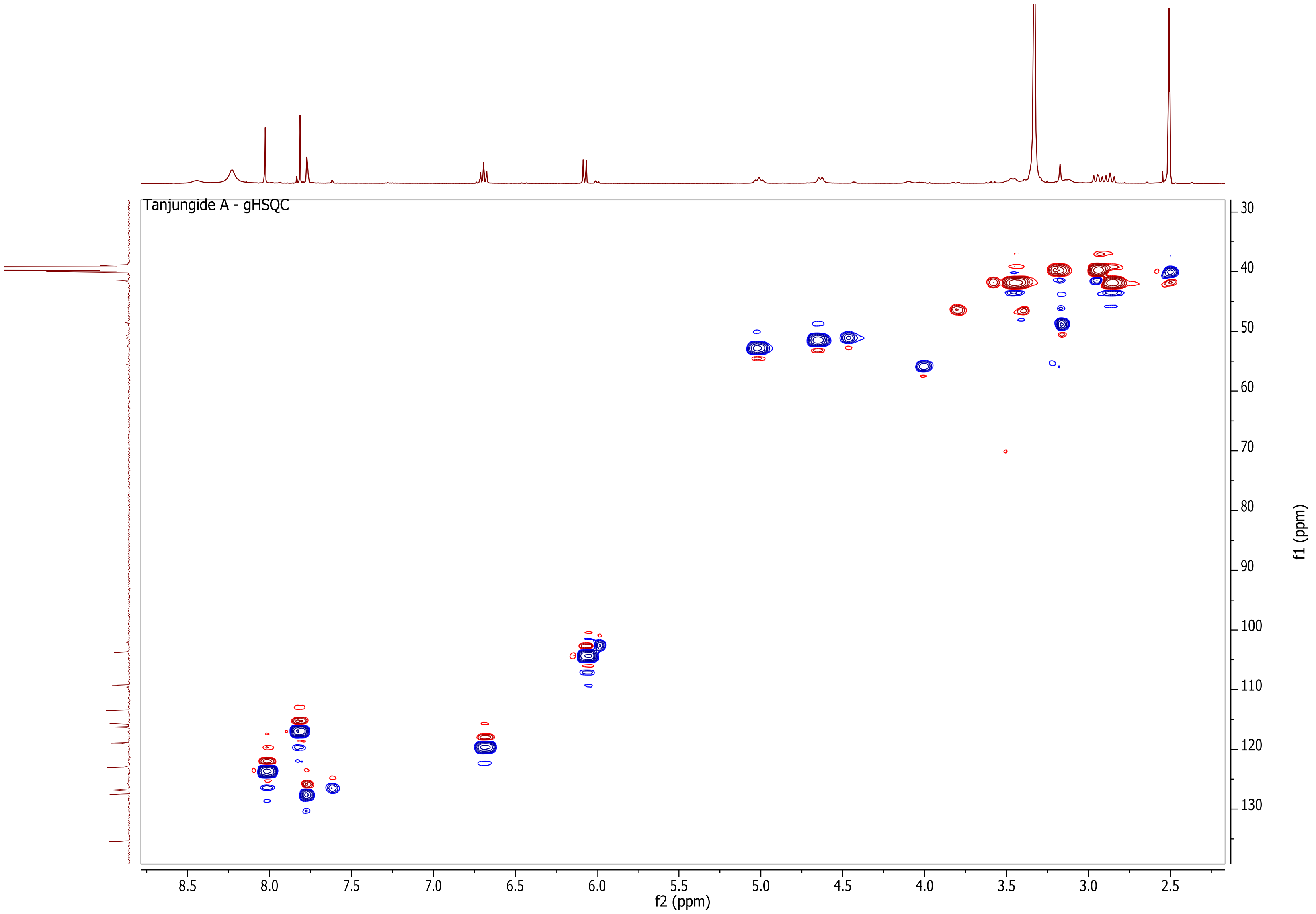

Figure S5. *g*-HMBC spectrum of Tanjungide A.

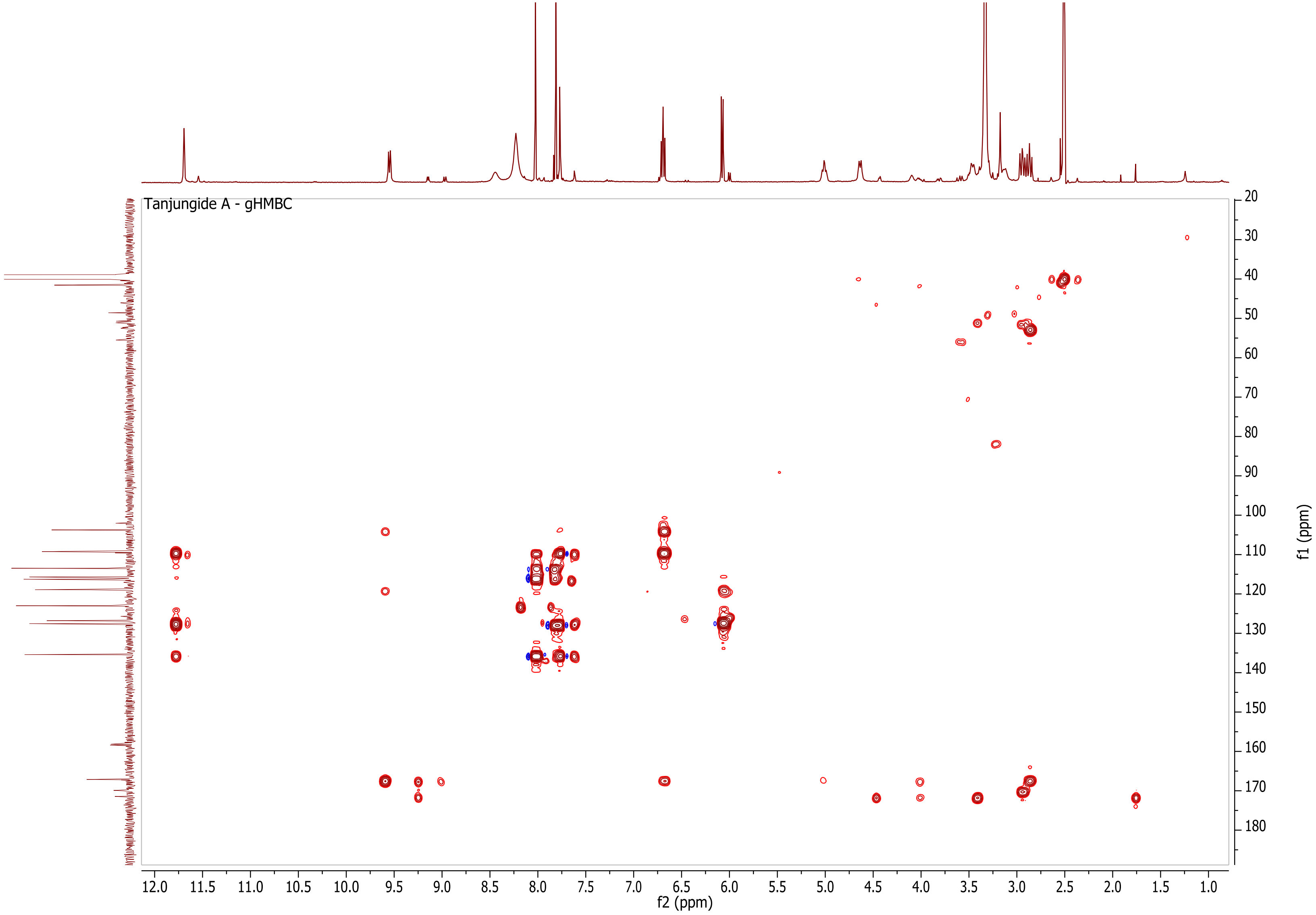

Figure S6. ROESY spectrum of Tanjungide A.

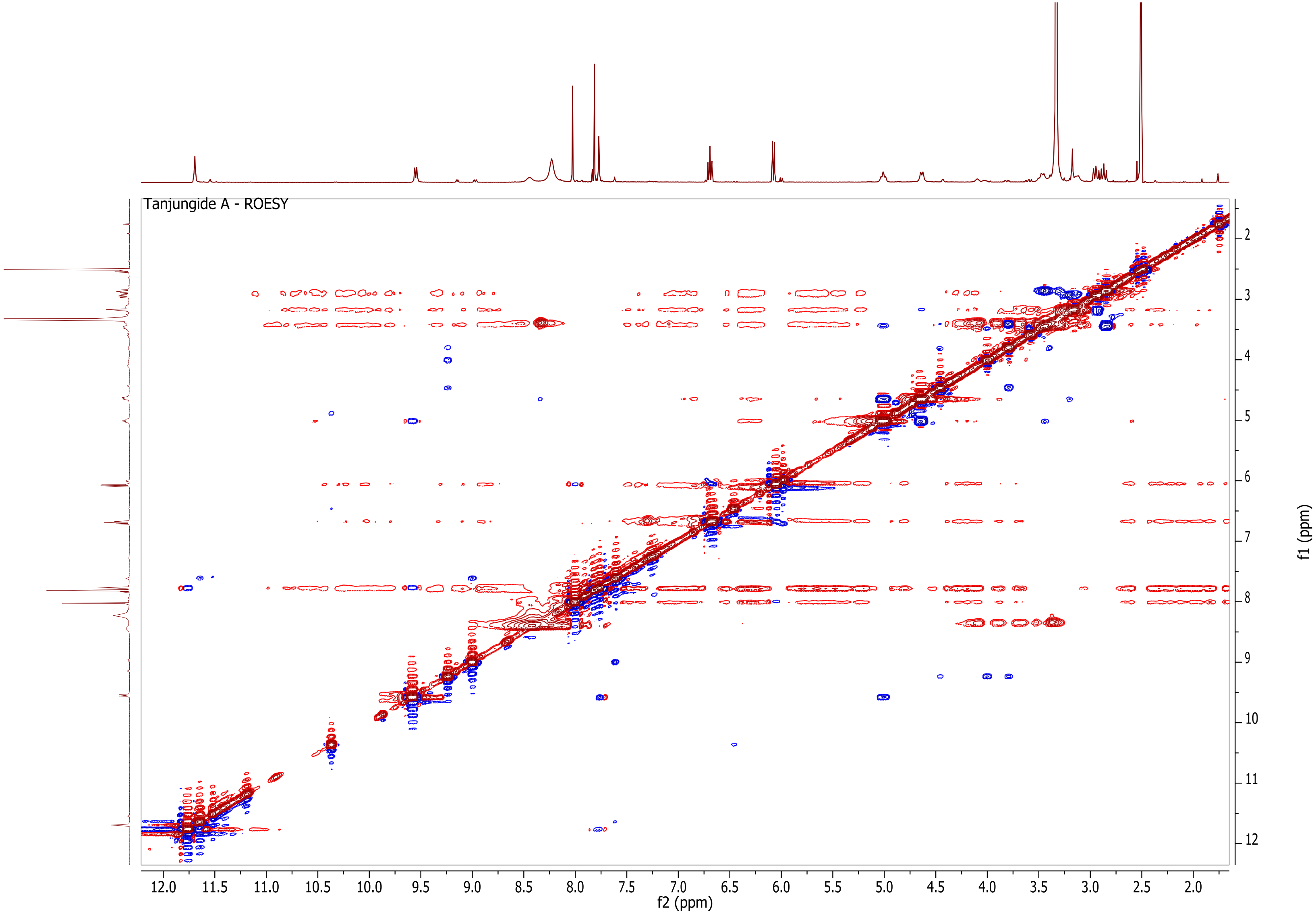

Figure S7. ^{1}H NMR spectrum of Tanjungide B (500 MHz, CD_3OD)

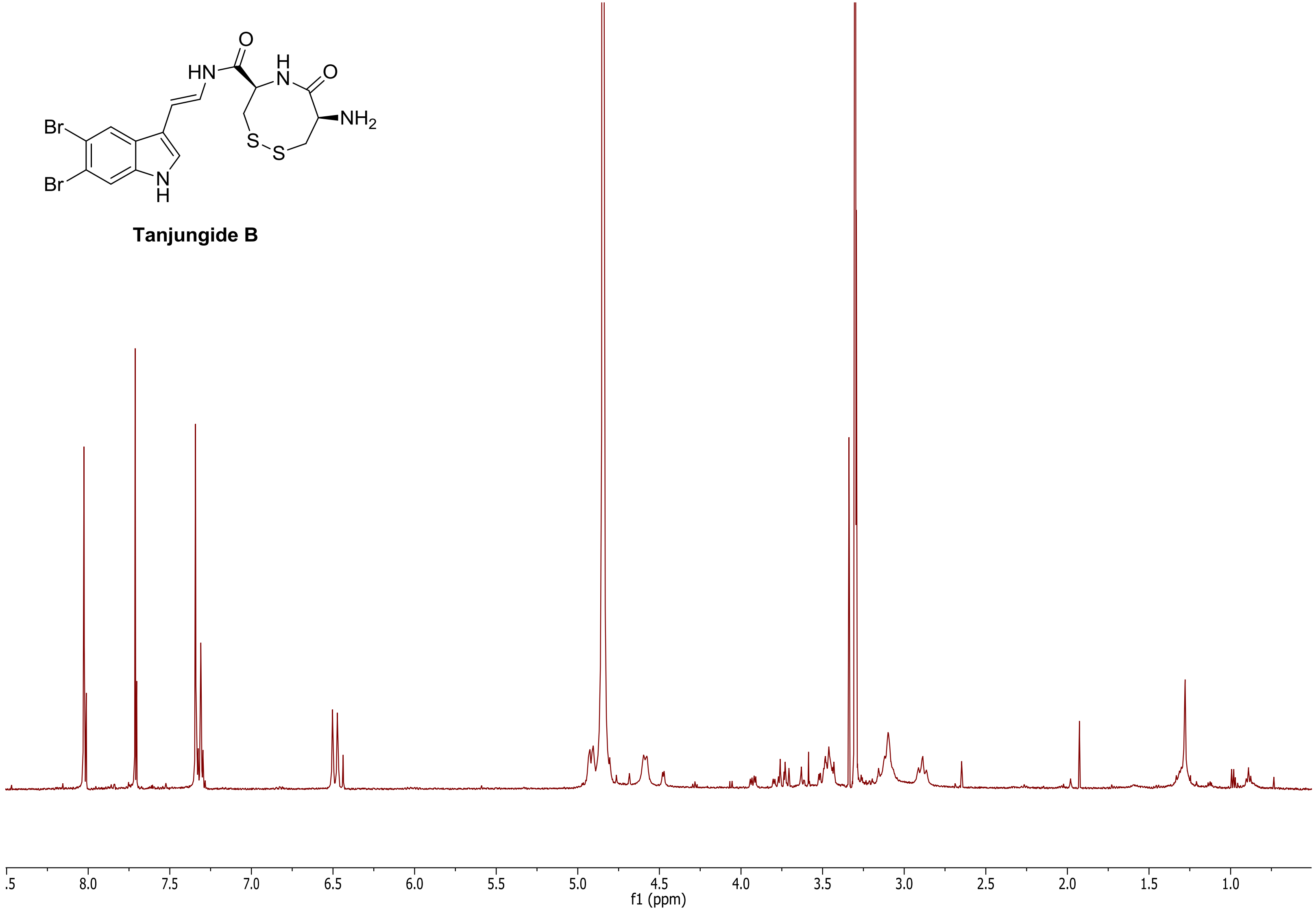

Figure S8. ^{13}C NMR spectrum of Tanjungide B (125 MHz, CD_3OD).

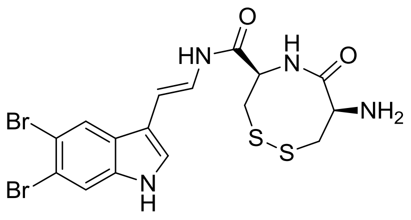

Tanjungide B

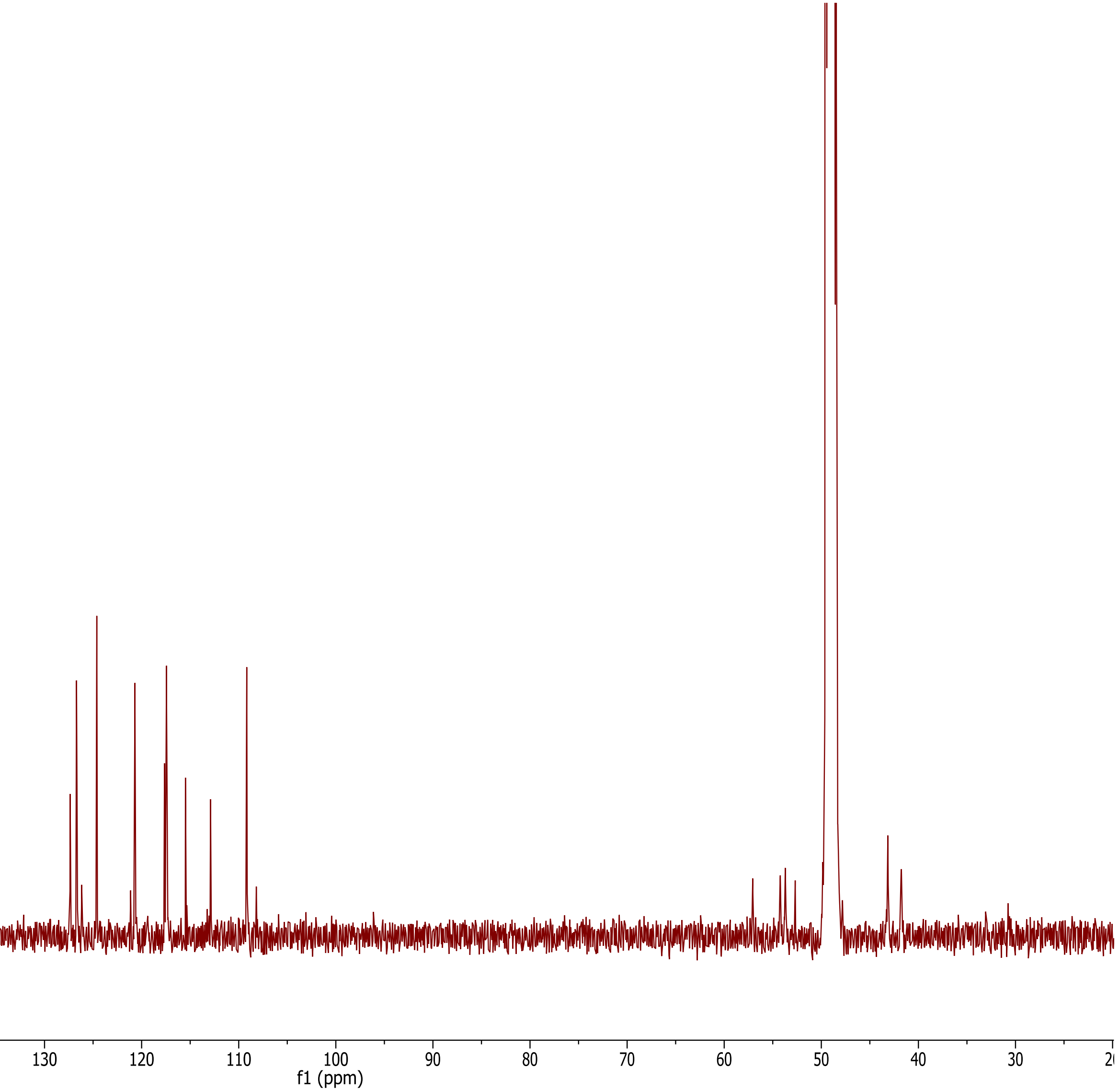

Figure S9. *g*-COSY spectrum Tanjungide B.

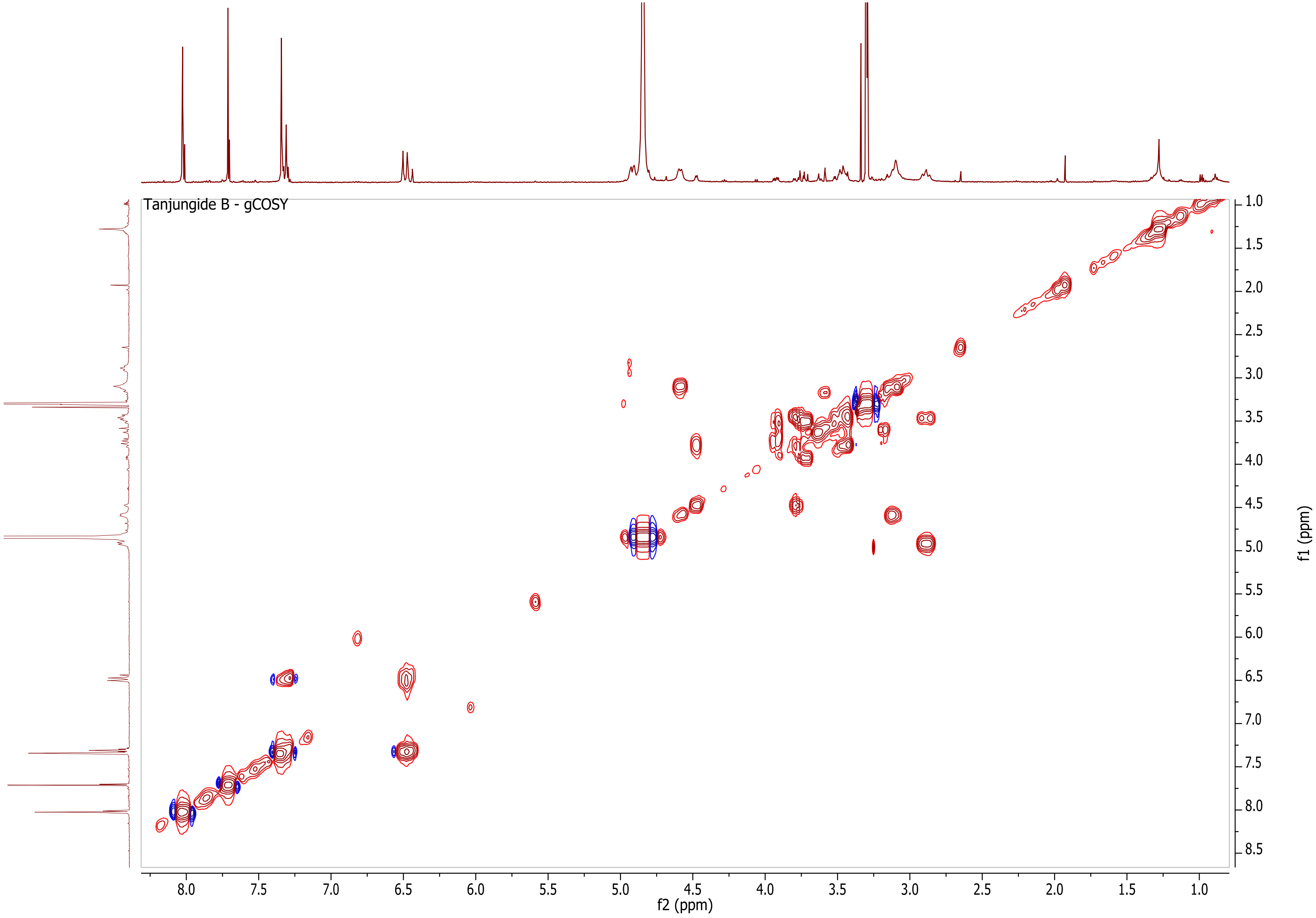

Figure S10. *g*-HSQC spectrum of Tanjungide B.

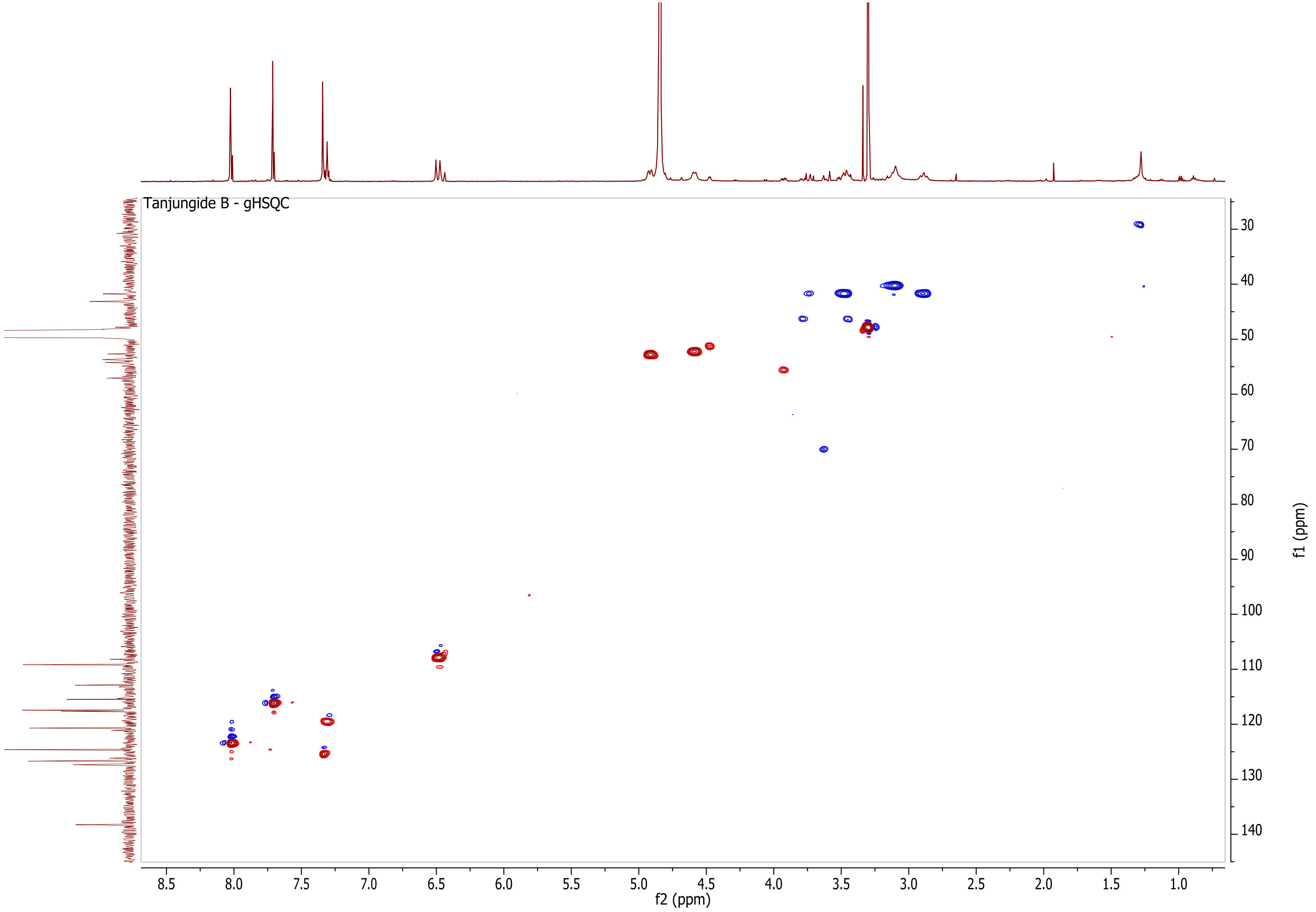

Figure S11. *g*-HMBC spectrum of Tanjungide B.

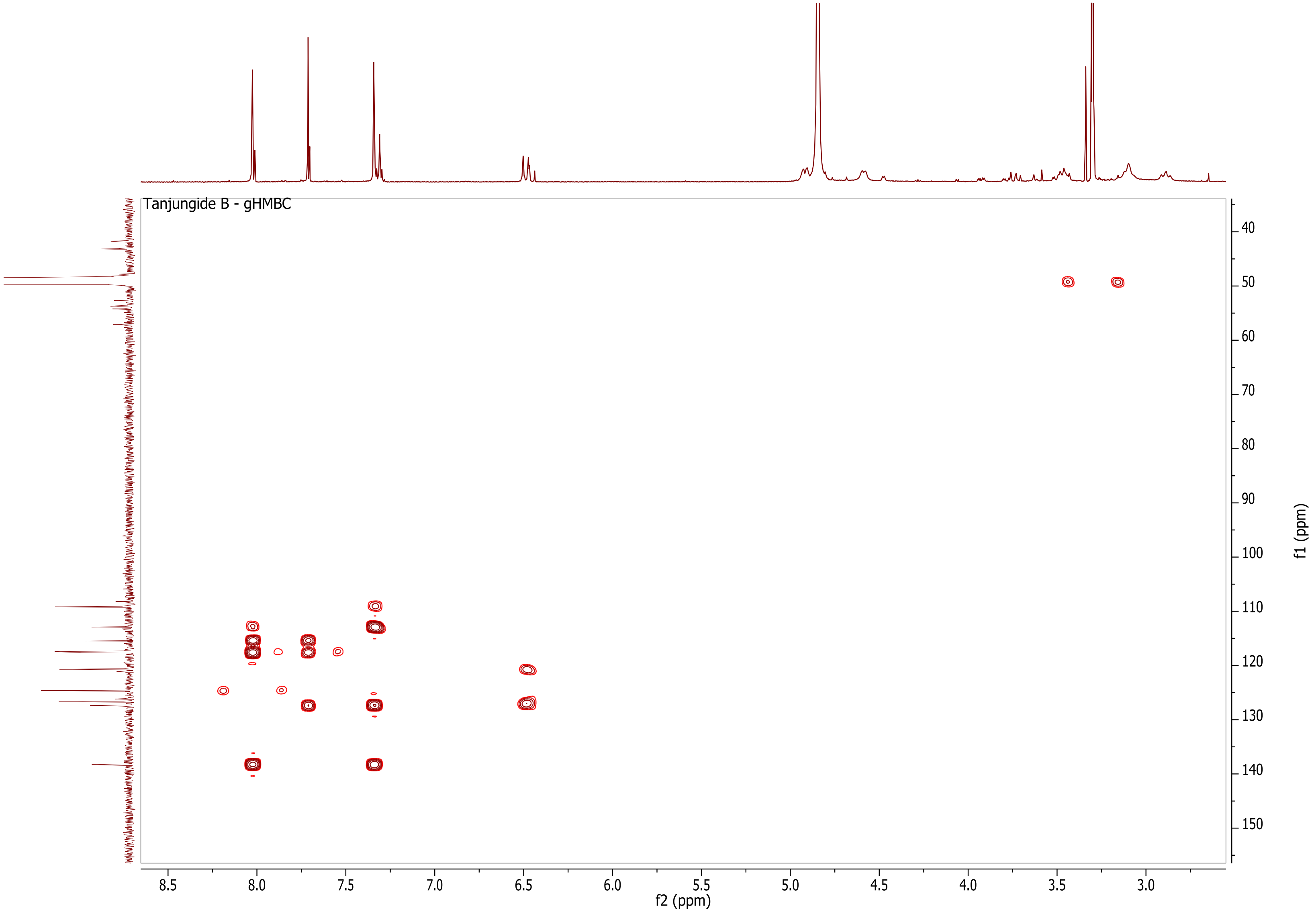

Figure S12. ROESY spectrum of Tanjungide B.

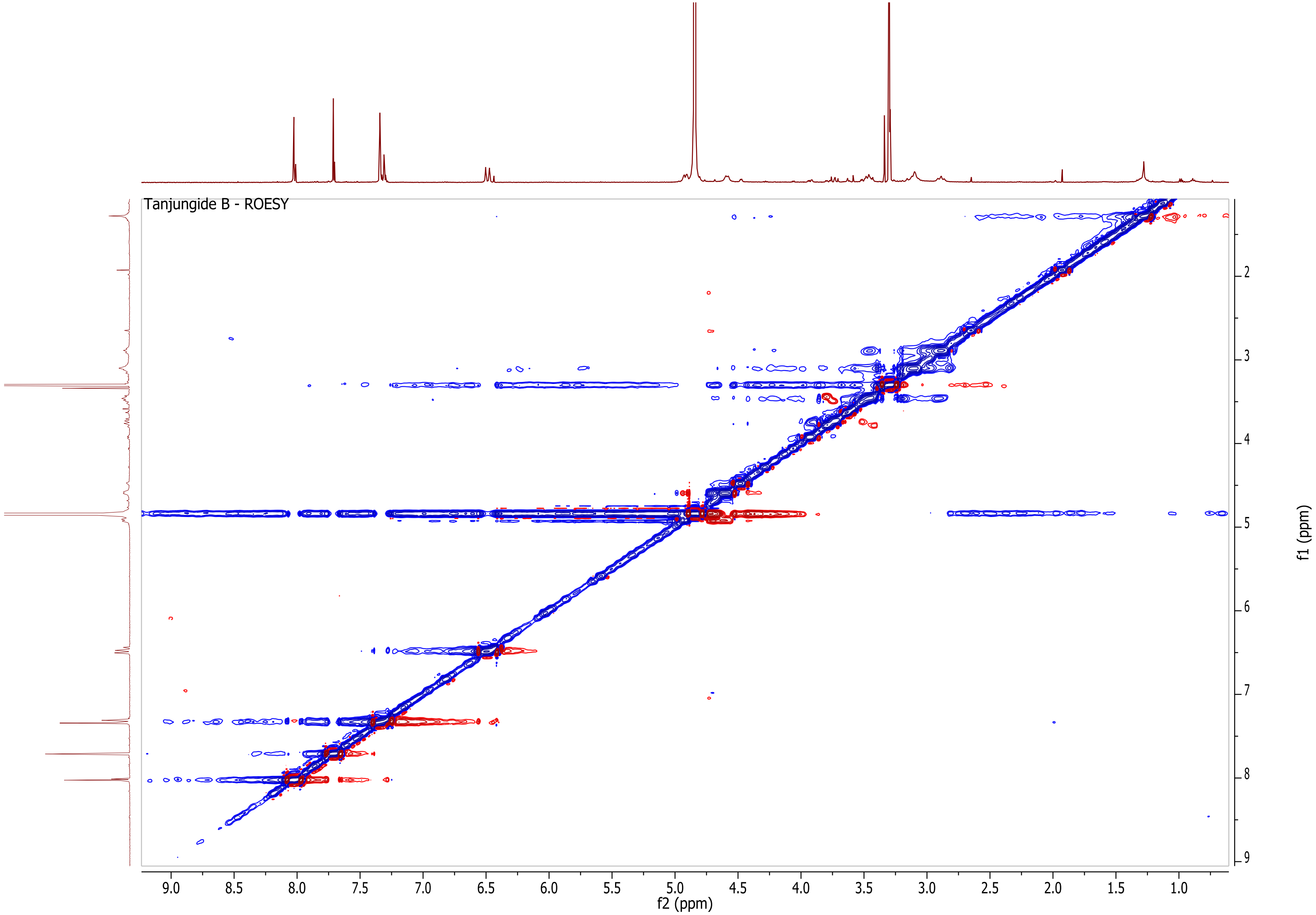

Figure S13. ^{1}H NMR spectrum of 5,6-dibromo-1*H*-indole-3-carboxylic acid (**4**) (300 MHz, CD_3OD).

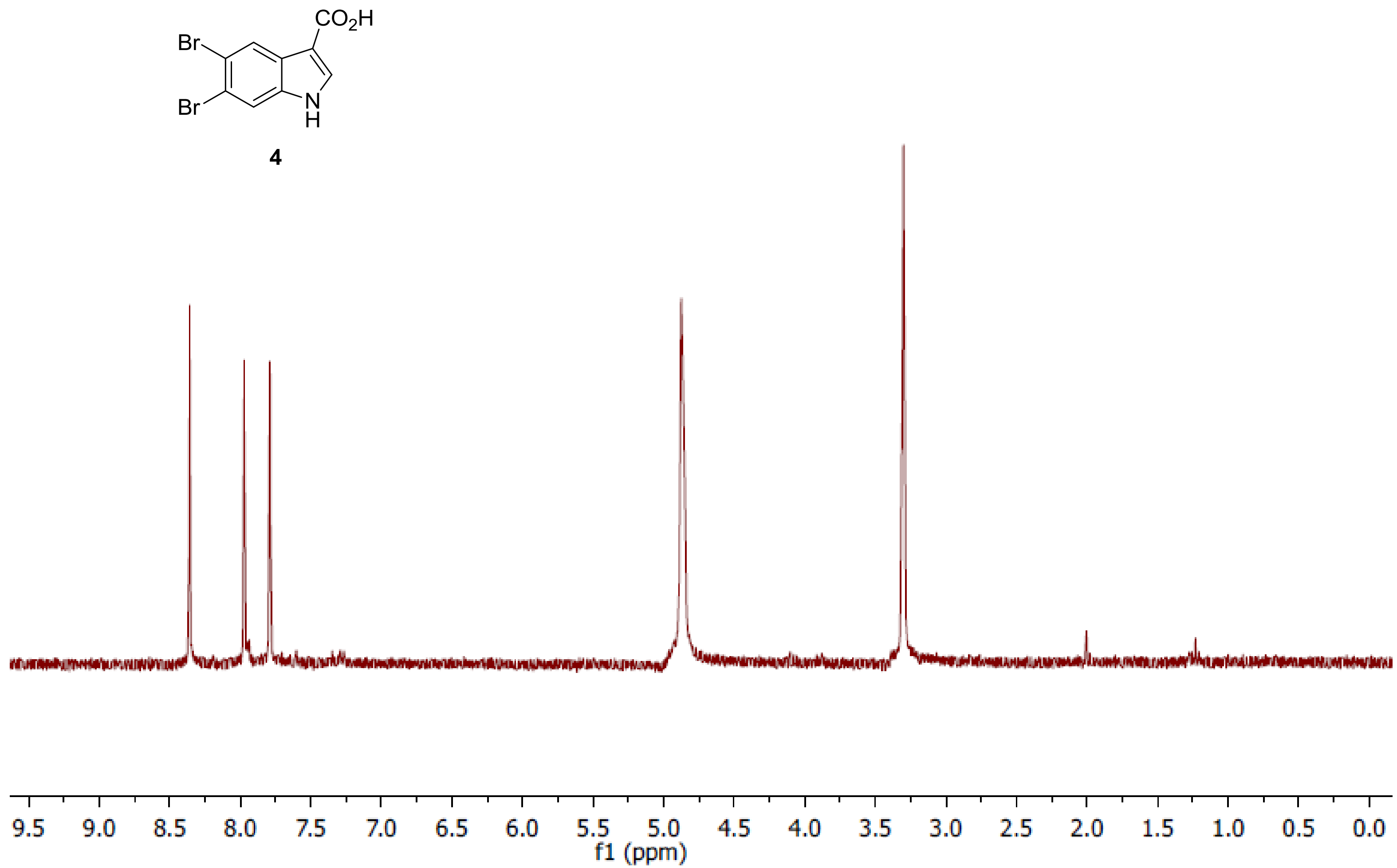

Figure S14. ^{13}C NMR spectrum of 5,6-dibromo-1*H*-indole-3-carboxylic acid (**4**) (75 MHz, CD_3OD).

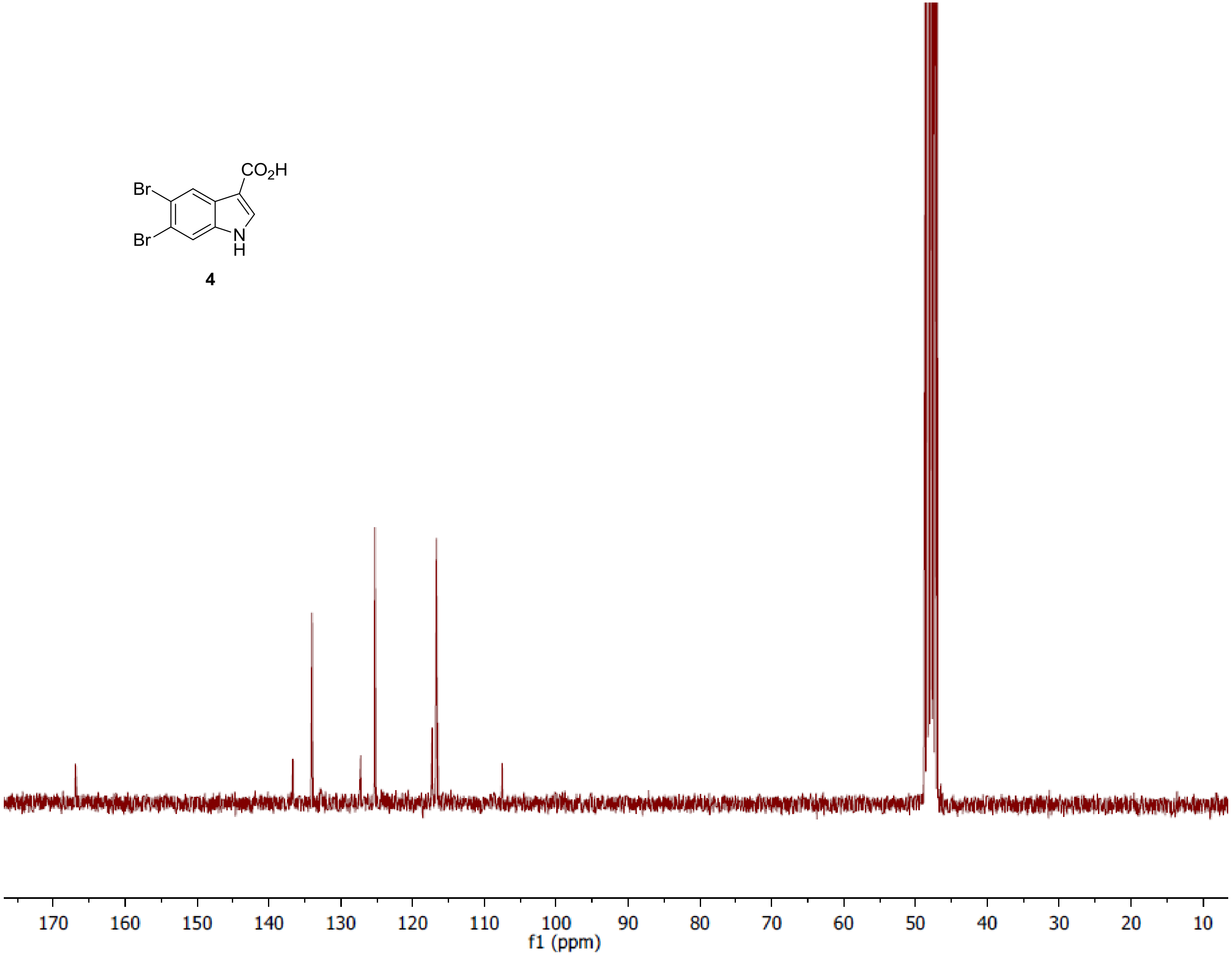

Figure S15. ^{1}H NMR spectrum of 5,6-dibromo-1*H*-indole (**5**) (300 MHz, $CDCl_3$).

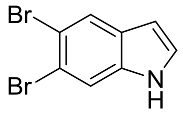

5

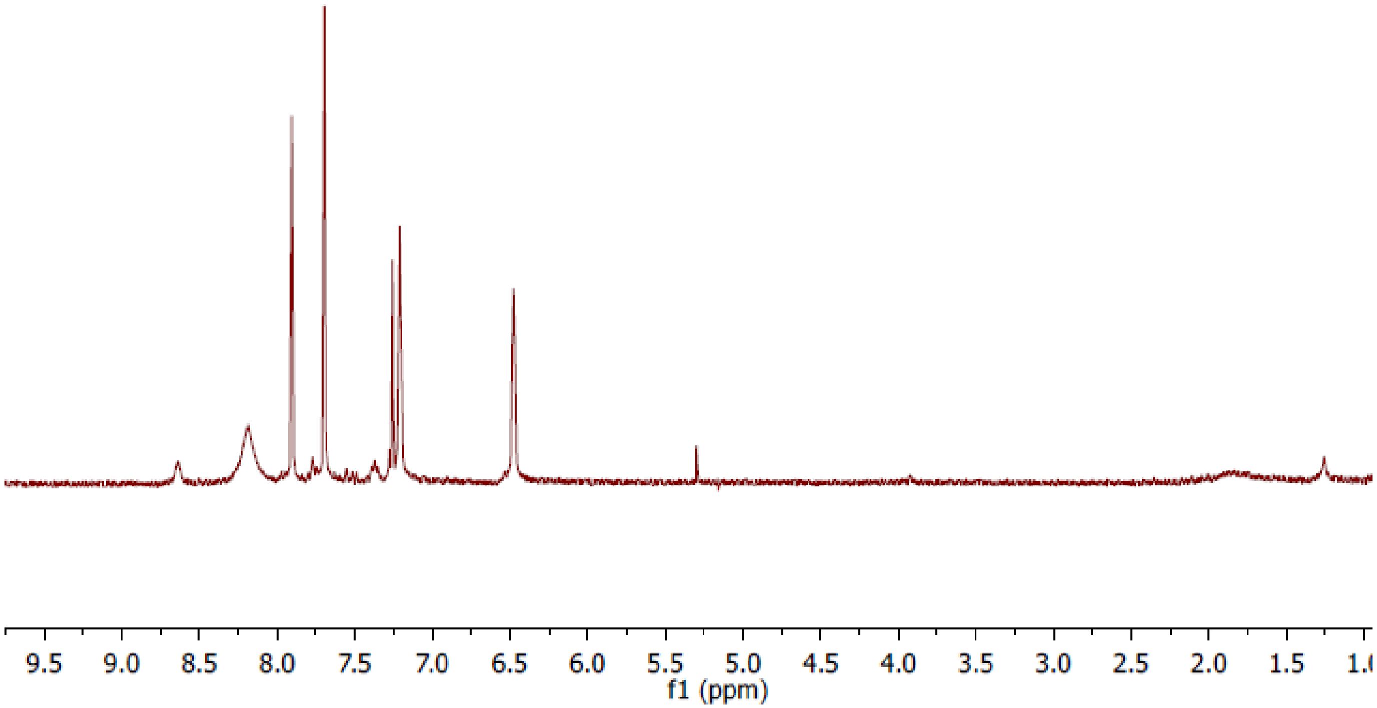

Figure S16. ^{13}C NMR spectrum of 5,6-dibromo-1*H*-indole (**5**) (75 MHz, $CDCl_3$).

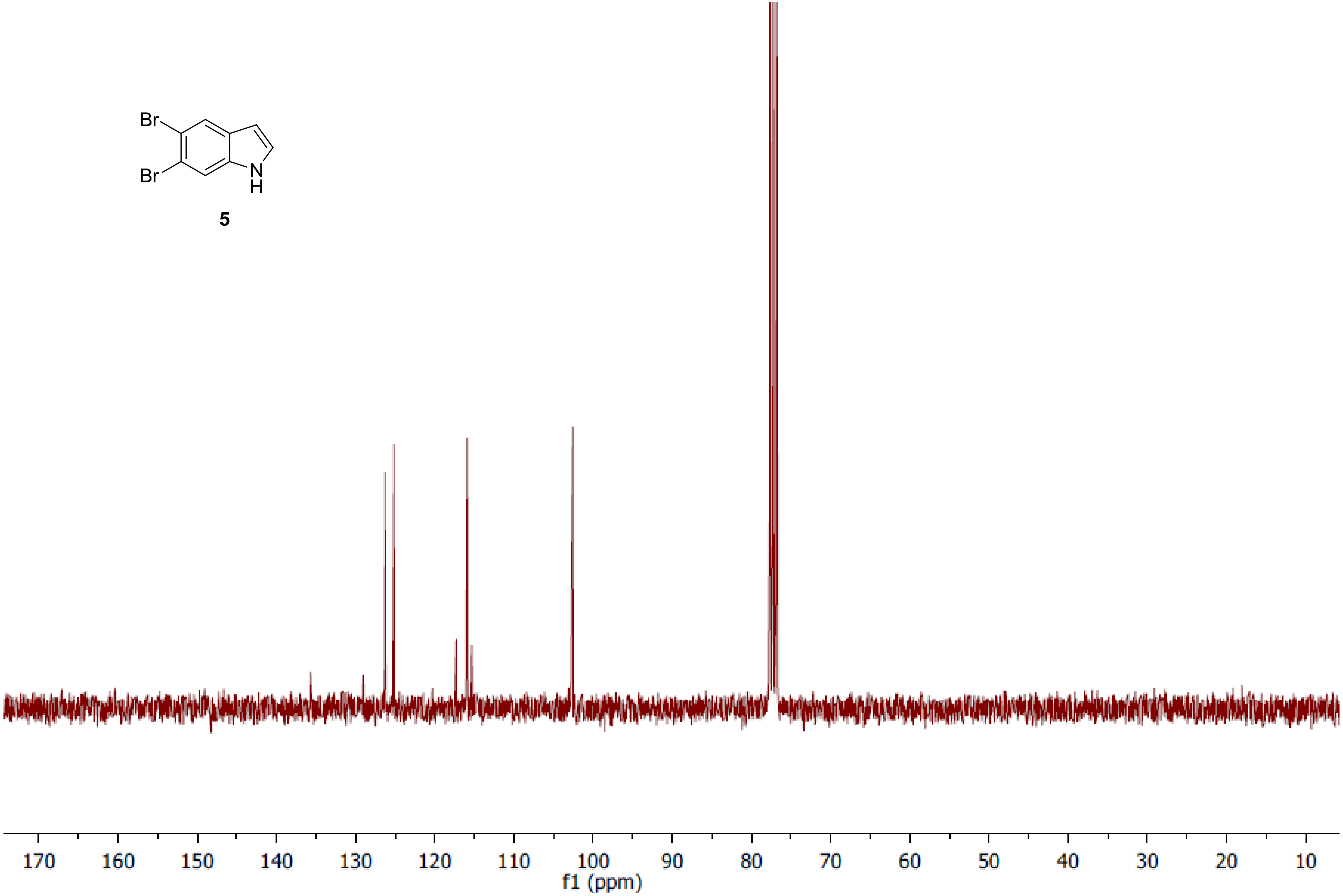

Figure S17. ^{1}H NMR spectrum of 5,6-dibromo-1*H*-indole-3-carbaldehyde (**6**) (300 MHz, DMSO-d_6).

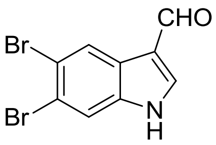

6

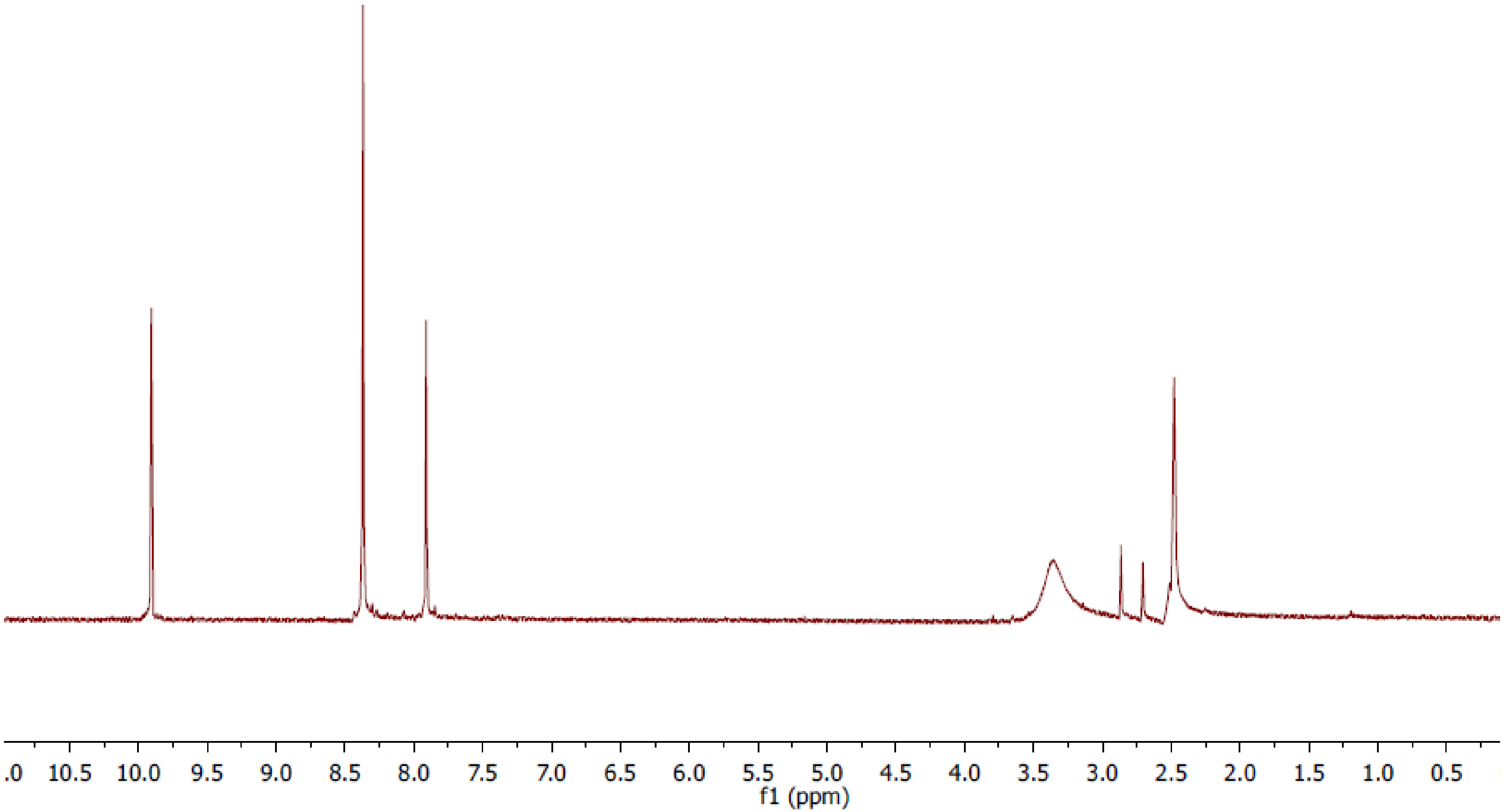

Figure S18. ^{13}C NMR spectrum of 5,6-dibromo-1*H*-indole-3-carbaldehyde (**6**) (75 MHz, DMSO-d_6).

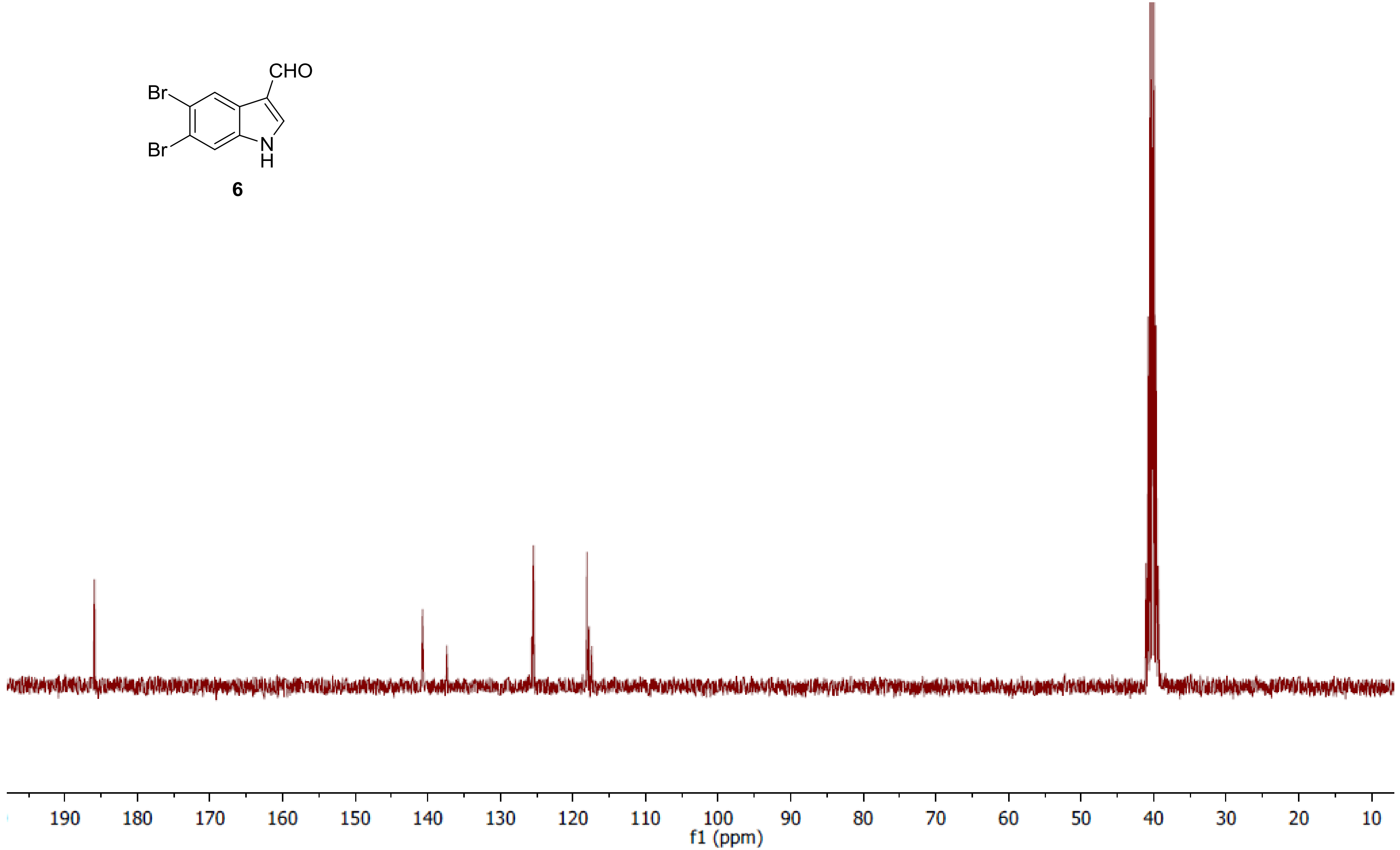

Figure S19. ^{1}H NMR spectrum of *tert*-butyl 5,6-dibromo-3-formyl-1*H*-indole-1-carboxylate (**7**) (300 MHz, $CDCl_3$).

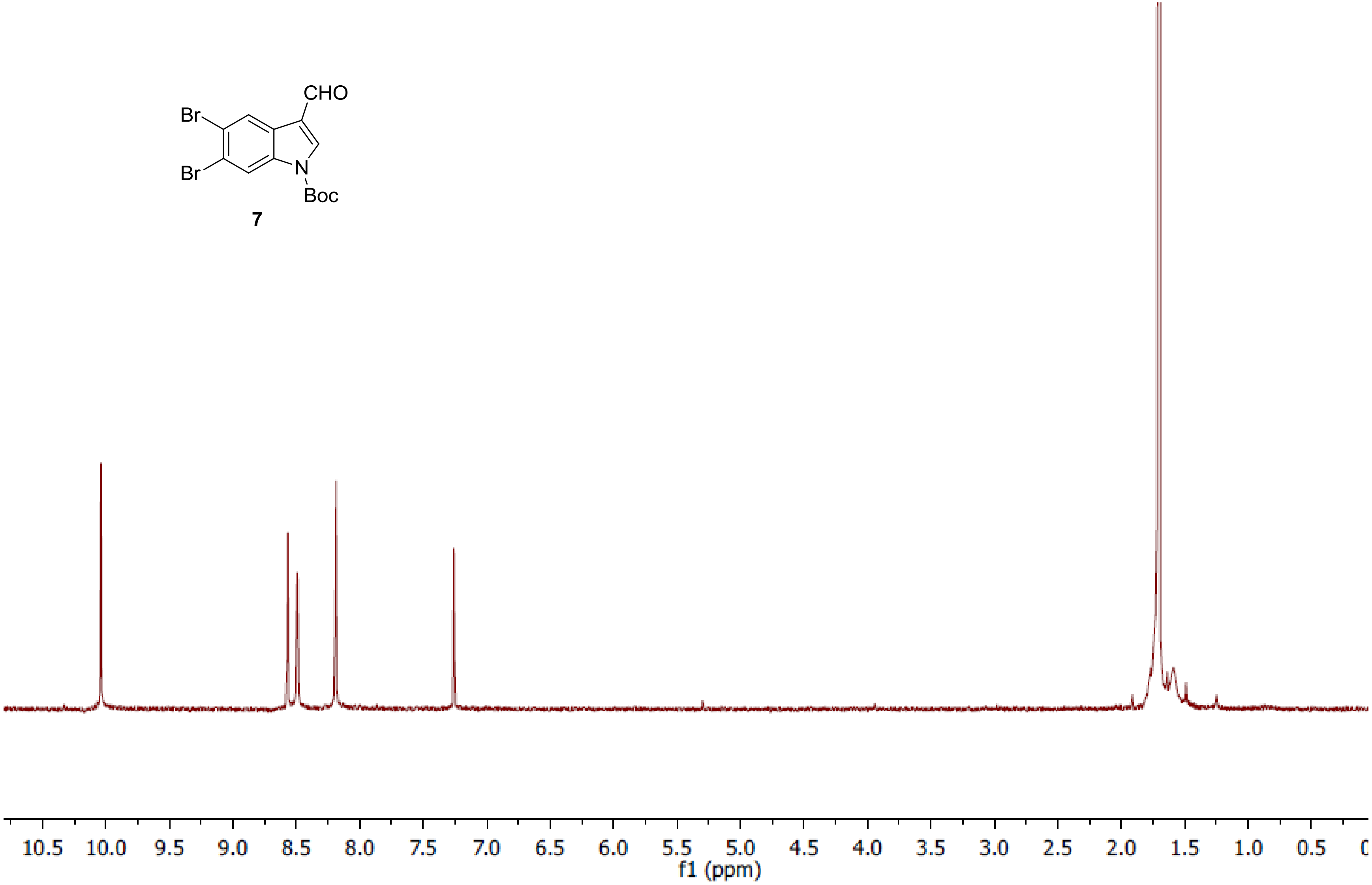

Figure S20. ^{13}C NMR spectrum of *tert*-butyl 5,6-dibromo-3-formyl-1*H*-indole-1-carboxylate (**7**) (75 MHz, $CDCl_3$).

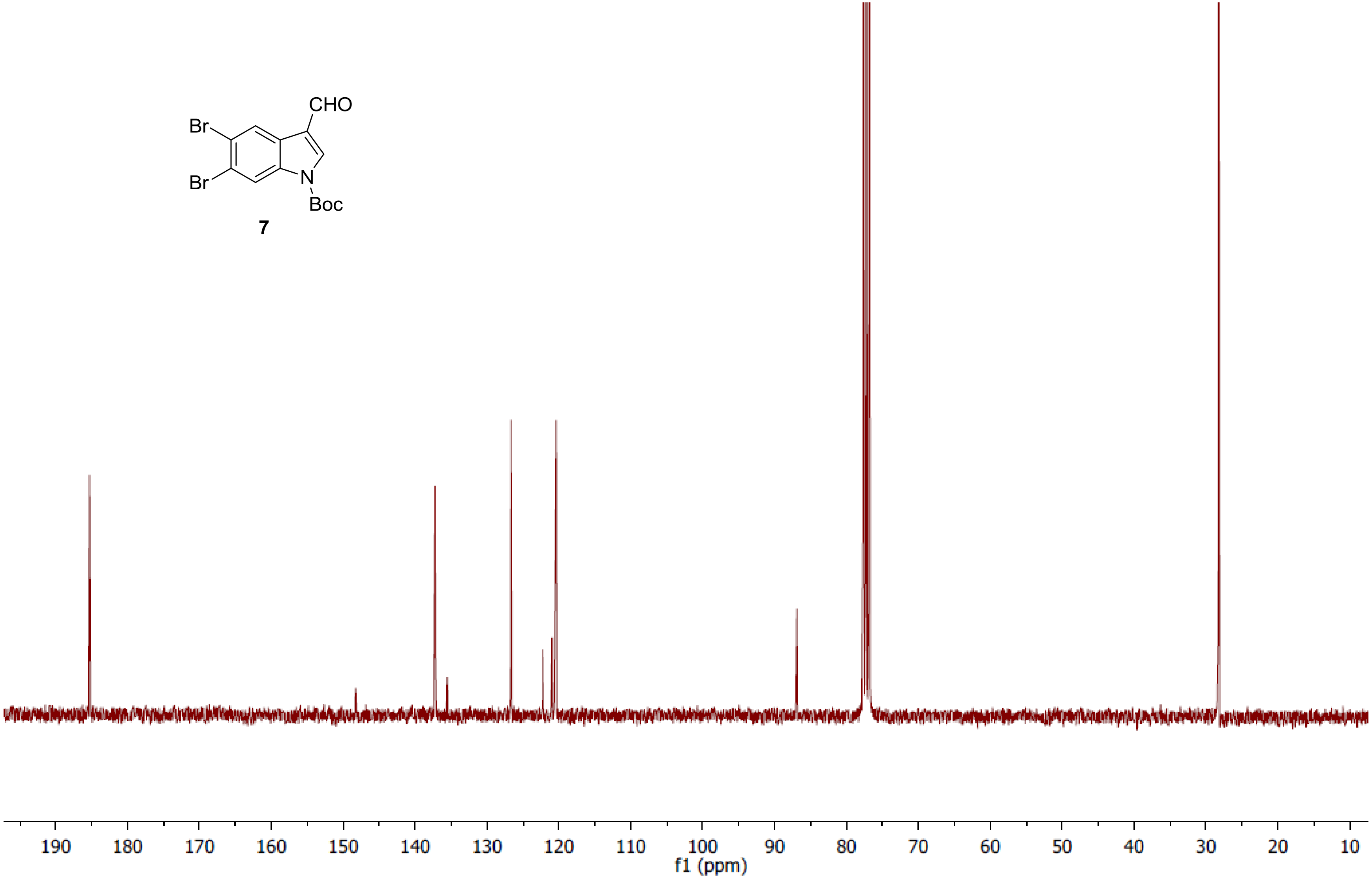

Figure S21. ^{1}H NMR spectrum of (*Z*)-*tert*-butyl 5,6-dibromo-3-(2-iodovinyl)-1*H*-indole-1-carboxylate (**8**) (300 MHz, $CDCl_3$).

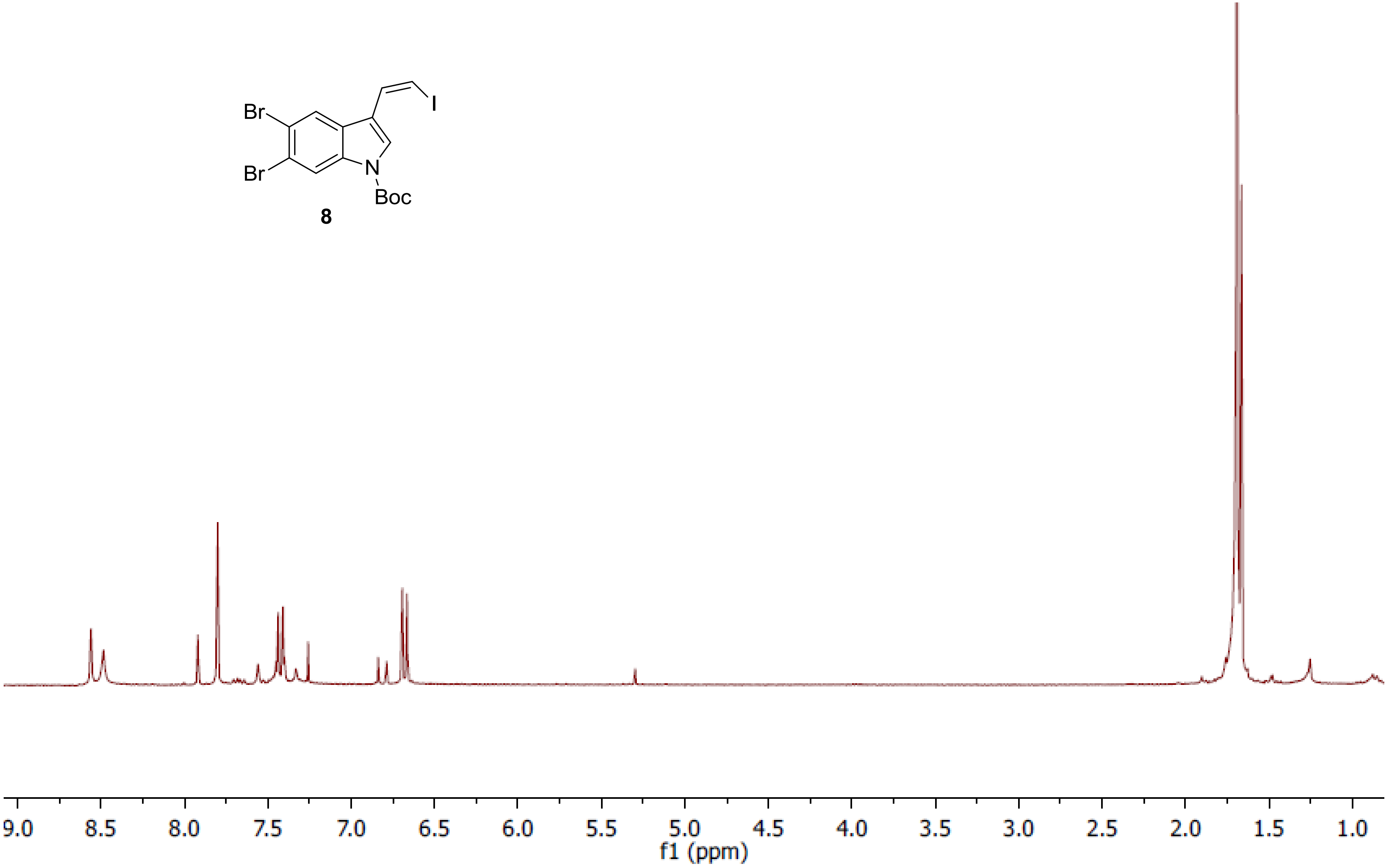

Figure S22. ^{13}C NMR spectrum of (*Z*)-*tert*-butyl 5,6-dibromo-3-(2-iodovinyl)-1*H*-indole-1-carboxylate (**8**) (75 MHz, $CDCl_3$).

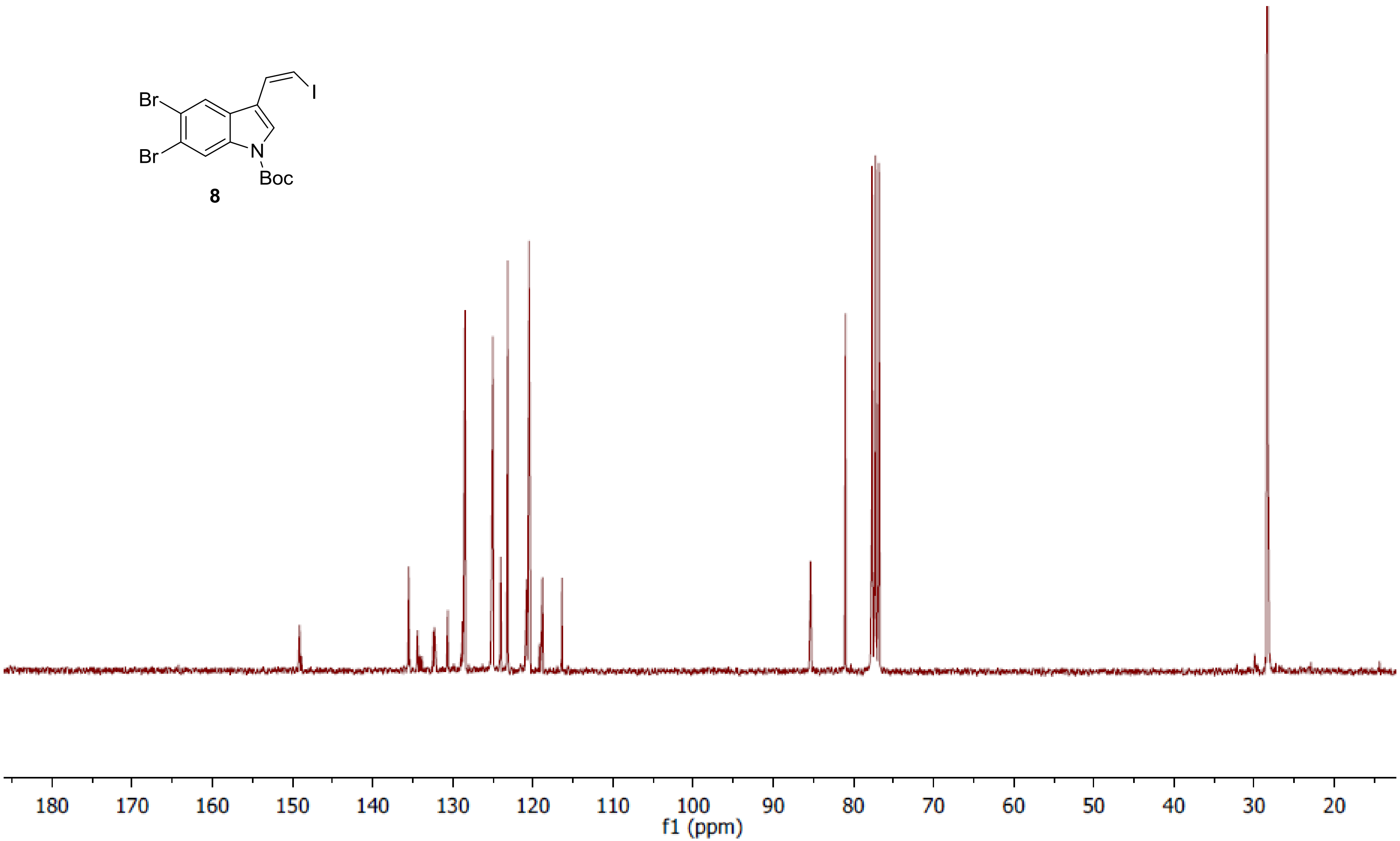

Figure S23. ^{1}H NMR spectrum of (*R*,*Z*)-*tert*-butyl 3-(2-(2-(((allyloxy)carbonyl)amino)-3-(tritylthio)propanamido)vinyl)-5,6-dibromo-1*H*-indole-1-carboxylate (*Z*-**10**) (300 MHz, $CDCl_3$).

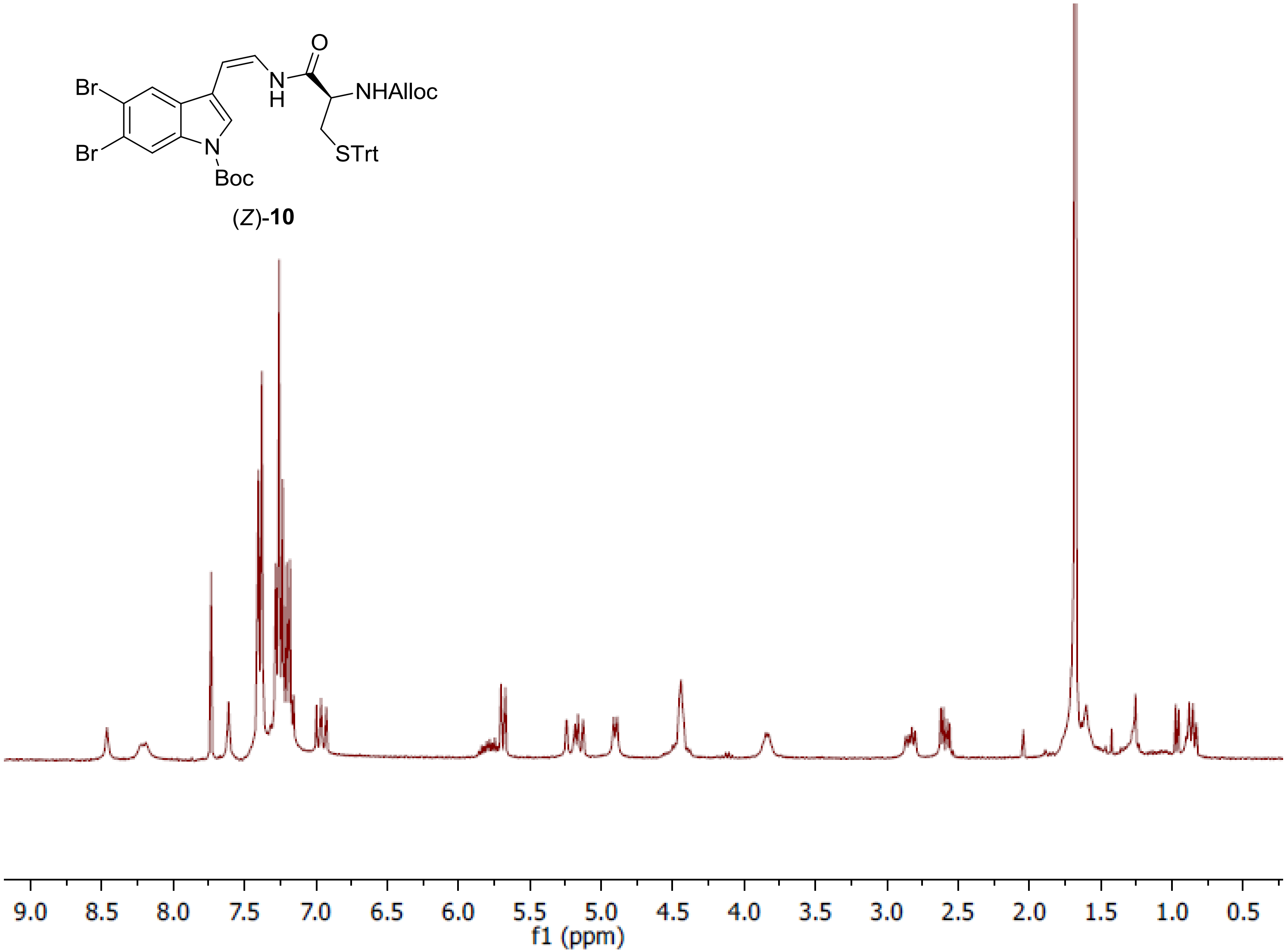

Figure S24. ^{13}C NMR spectrum of (*R*,*Z*)-*tert*-butyl 3-(2-(2-(((allyloxy)carbonyl)amino)-3-(tritylthio)propanamido)vinyl)-5,6-dibromo-1*H*-indole-1-carboxylate (*Z*-**10**) (75 MHz, CDCl$_3$).

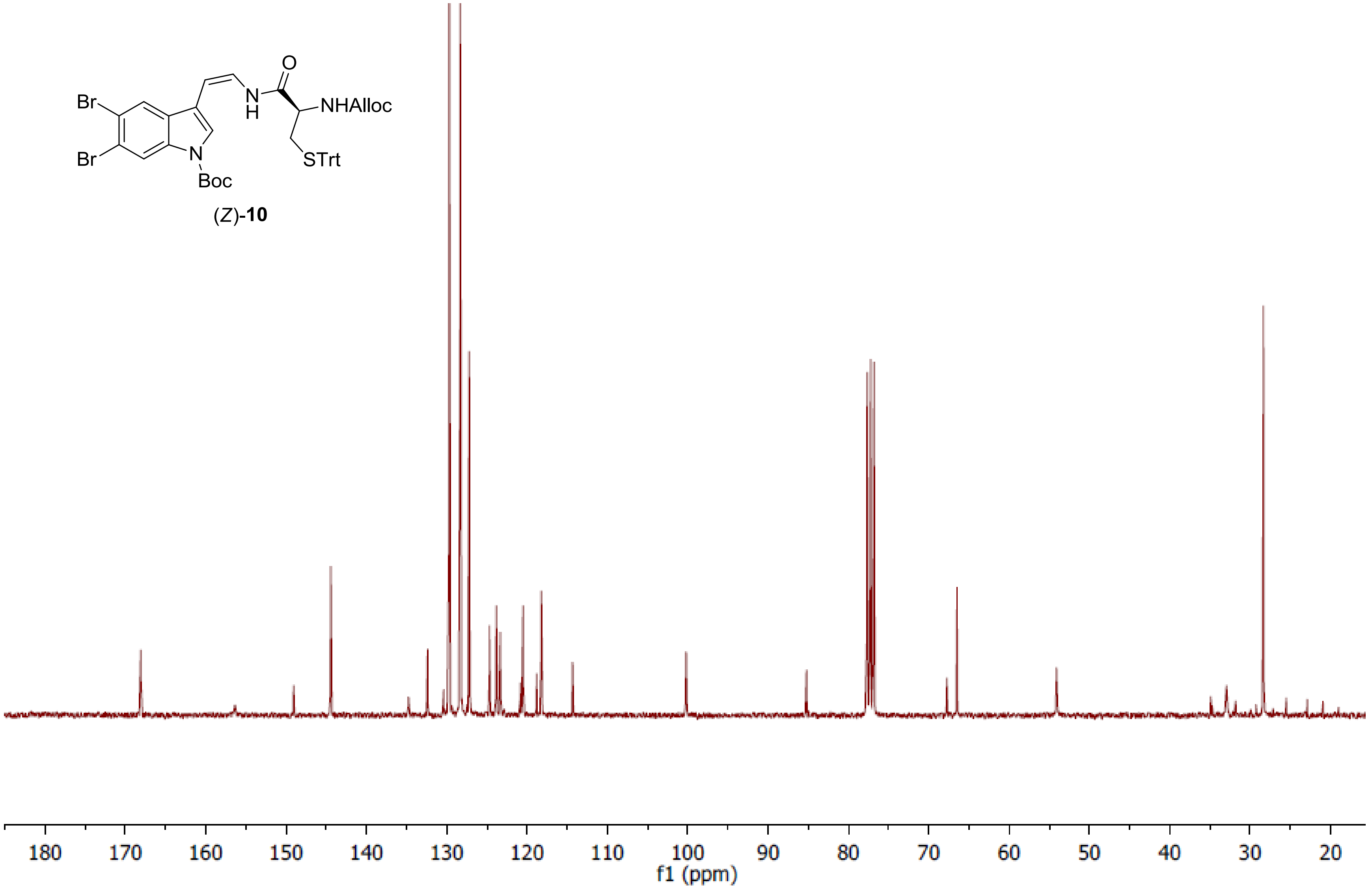

Figure S25. ^{1}H NMR spectrum of (*R*,*E*)-*tert*-butyl 3-(2-(2-(((allyloxy)carbonyl)amino)-3-(tritylthio)propanamido)vinyl)-5,6-dibromo-1*H*-indole-1-carboxylate (*E*-**10**) (300 MHz, $CDCl_3$).

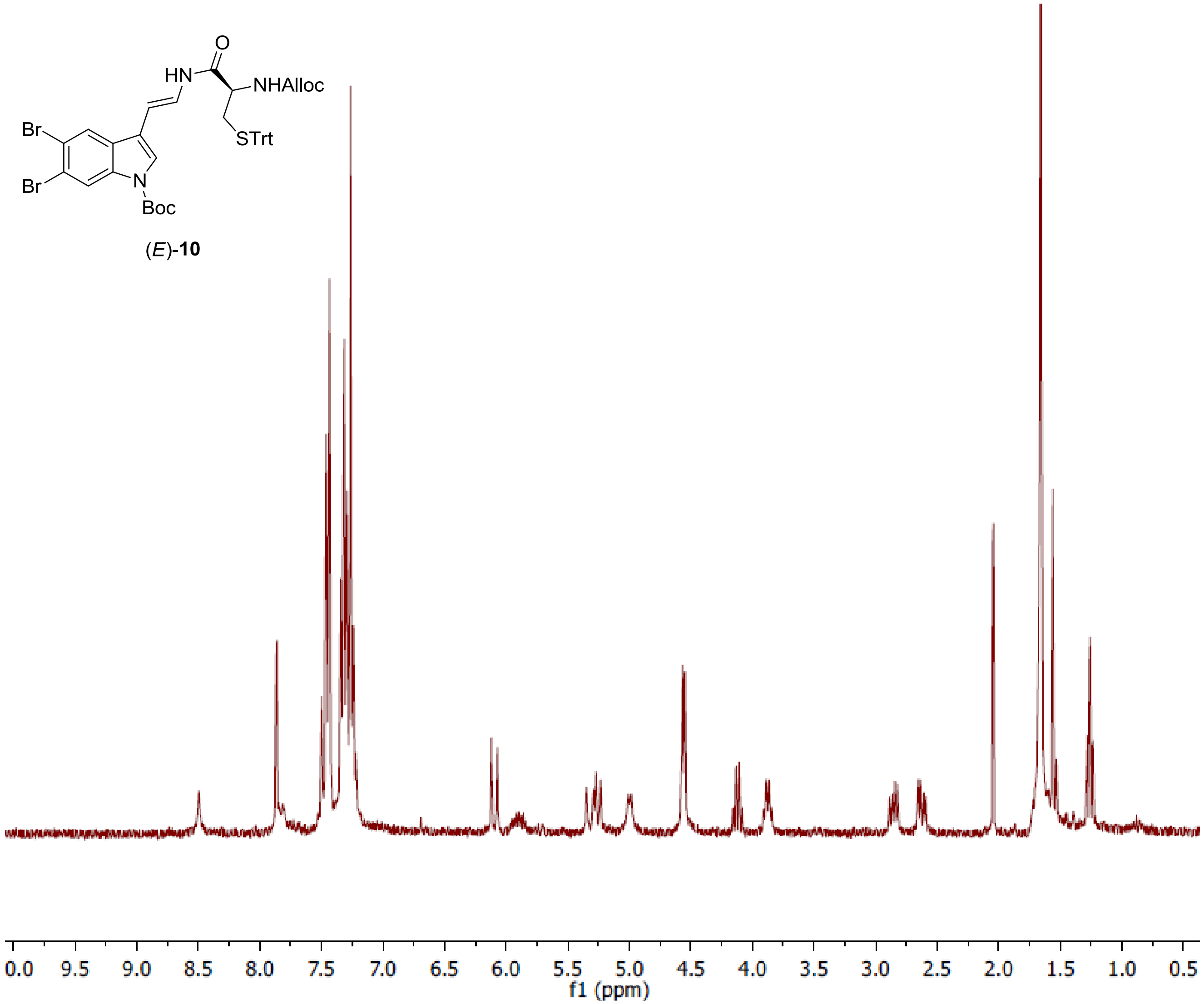

Figure S26. ^{13}C NMR spectrum of (*R*,*E*)-*tert*-butyl 3-(2-(2-(((allyloxy)carbonyl)amino)-3-(tritylthio)propanamido)vinyl)-5,6-dibromo-1*H*-indole-1-carboxylate (*E*-**10**) (75 MHz, CDCl$_3$).

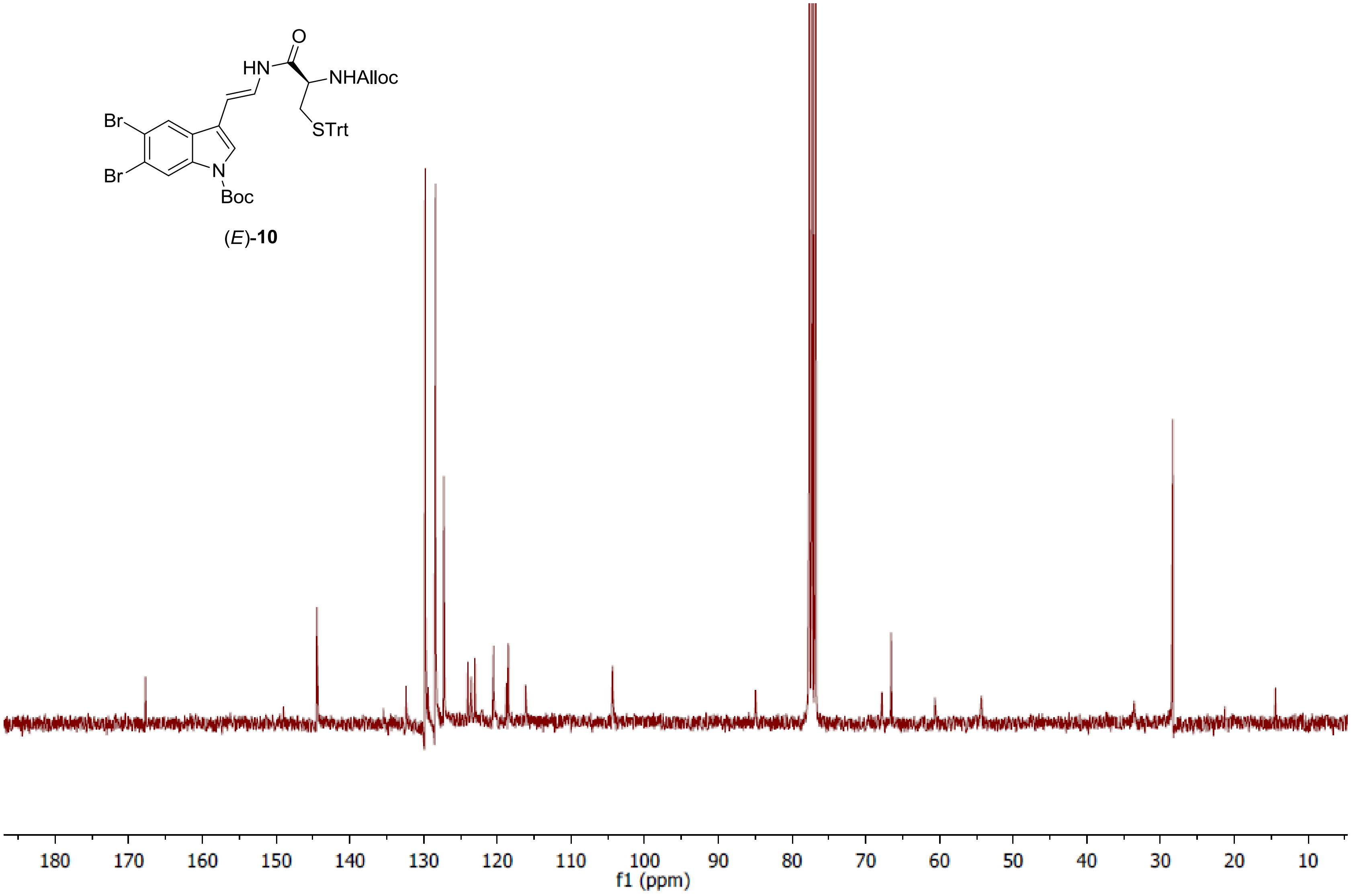

Figure S27. ^{1}H NMR spectrum of (*R*,*Z*)-*tert*-butyl 3-(2-(2-amino-3-(tritylthio)propanamido)vinyl)-5,6-dibromo-1*H*-indole-1-carboxylate (*Z*-**11**) (300 MHz, $CDCl_3$).

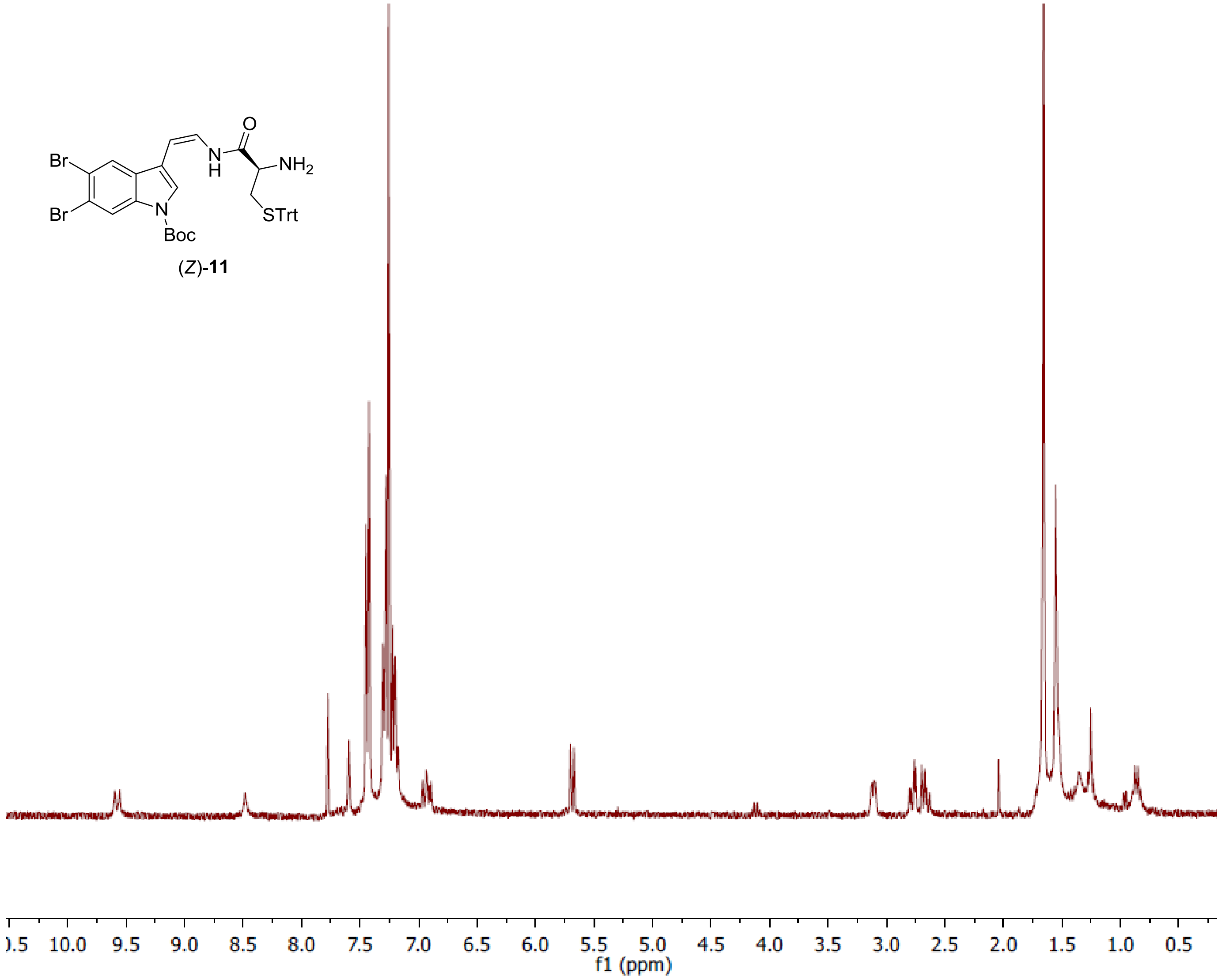

Figure S28. ^{13}C NMR spectrum of (*R*,*Z*)-*tert*-butyl 3-(2-(2-amino-3-(tritylthio)propanamido)vinyl)-5,6-dibromo-1*H*-indole-1-carboxylate (*Z*-**11**) (75 MHz, $CDCl_3$).

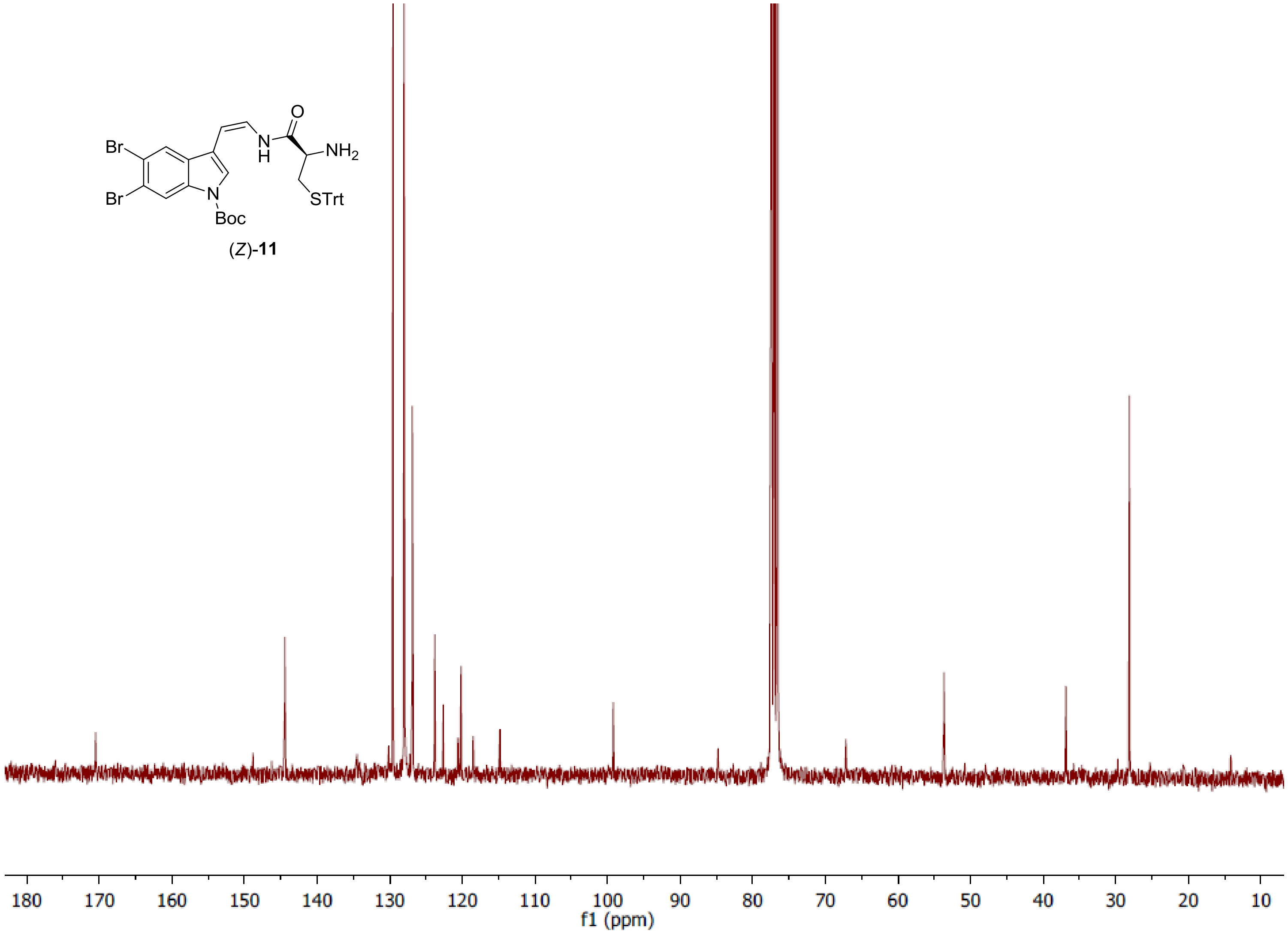

Figure S29. ^{1}H NMR spectrum of (*R*,*E*)-*tert*-butyl 3-(2-(2-amino-3-(tritylthio)propanamido)vinyl)-5,6-dibromo-1*H*-indole-1-carboxylate (*E*-**11**) (300 MHz, $CDCl_3$).

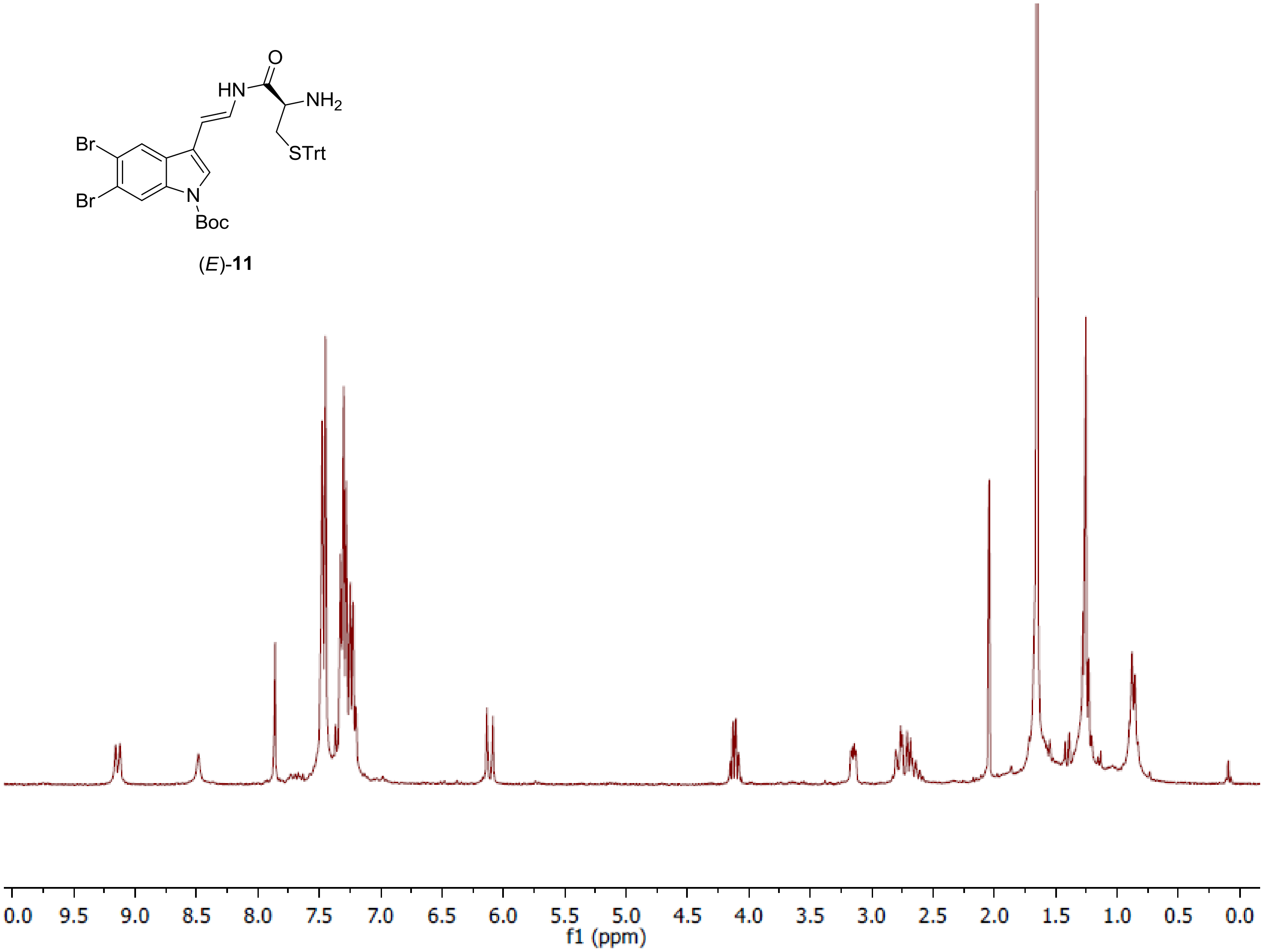

Figure S30. ^{13}C NMR spectrum of (*R*,*E*)-*tert*-butyl 3-(2-(2-amino-3-(tritylthio)propanamido)vinyl)-5,6-dibromo-1*H*-indole-1-carboxylate (*E*-**11**) (75 MHz, $CDCl_3$).

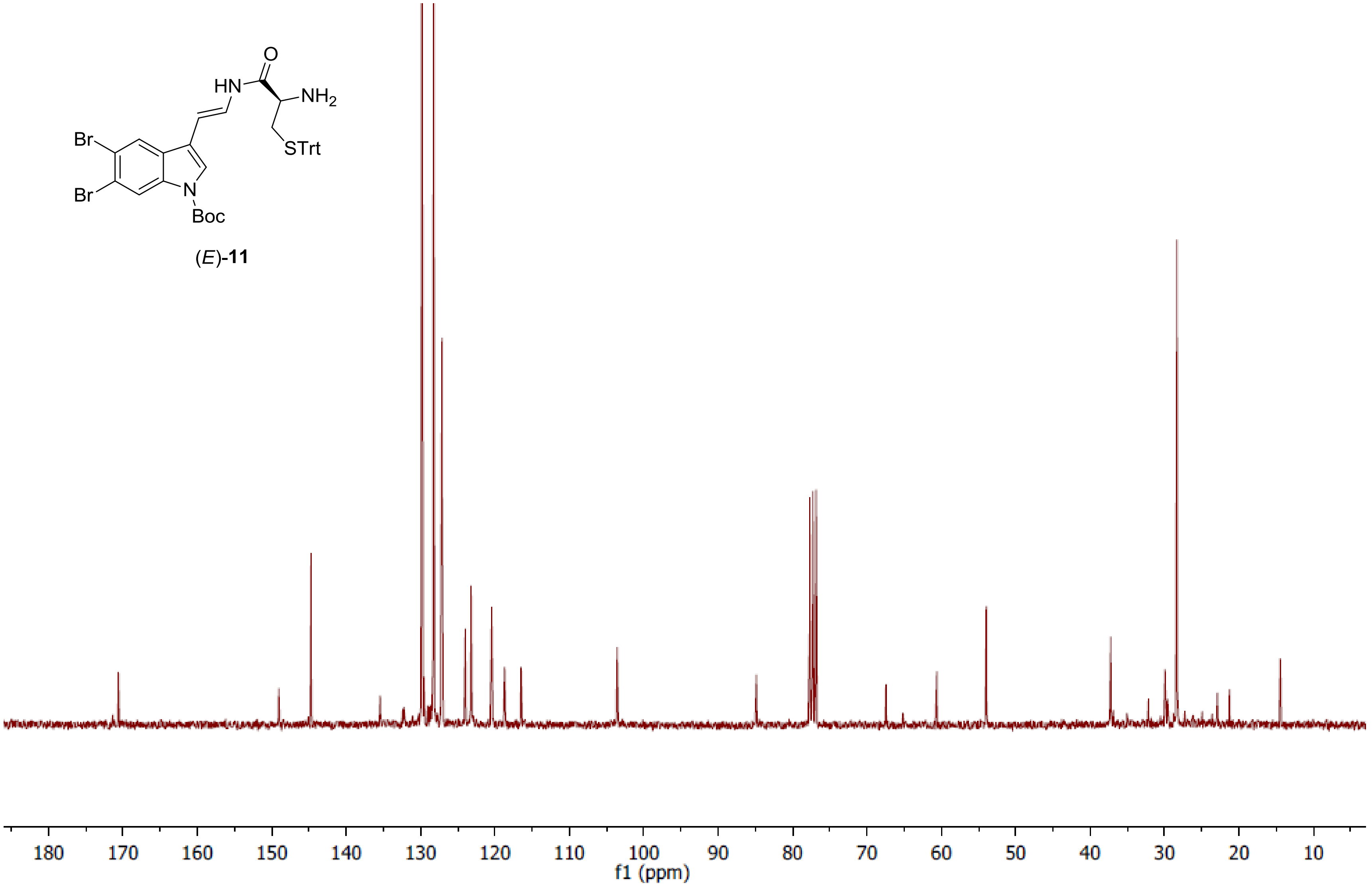

Figure S31. ^{1}H NMR spectrum of *tert*-butyl 5,6-dibromo-3-((6*R*,9*R*,*Z*)-2,2-dimethyl-4,7,10-trioxo-6,9-bis((tritylthio)methyl)-3-oxa-5,8,11-triazatridec-12-en-13-yl)-1*H*-indole-1-carboxylate (*Z*-**12**) (300 MHz, $CDCl_3$).

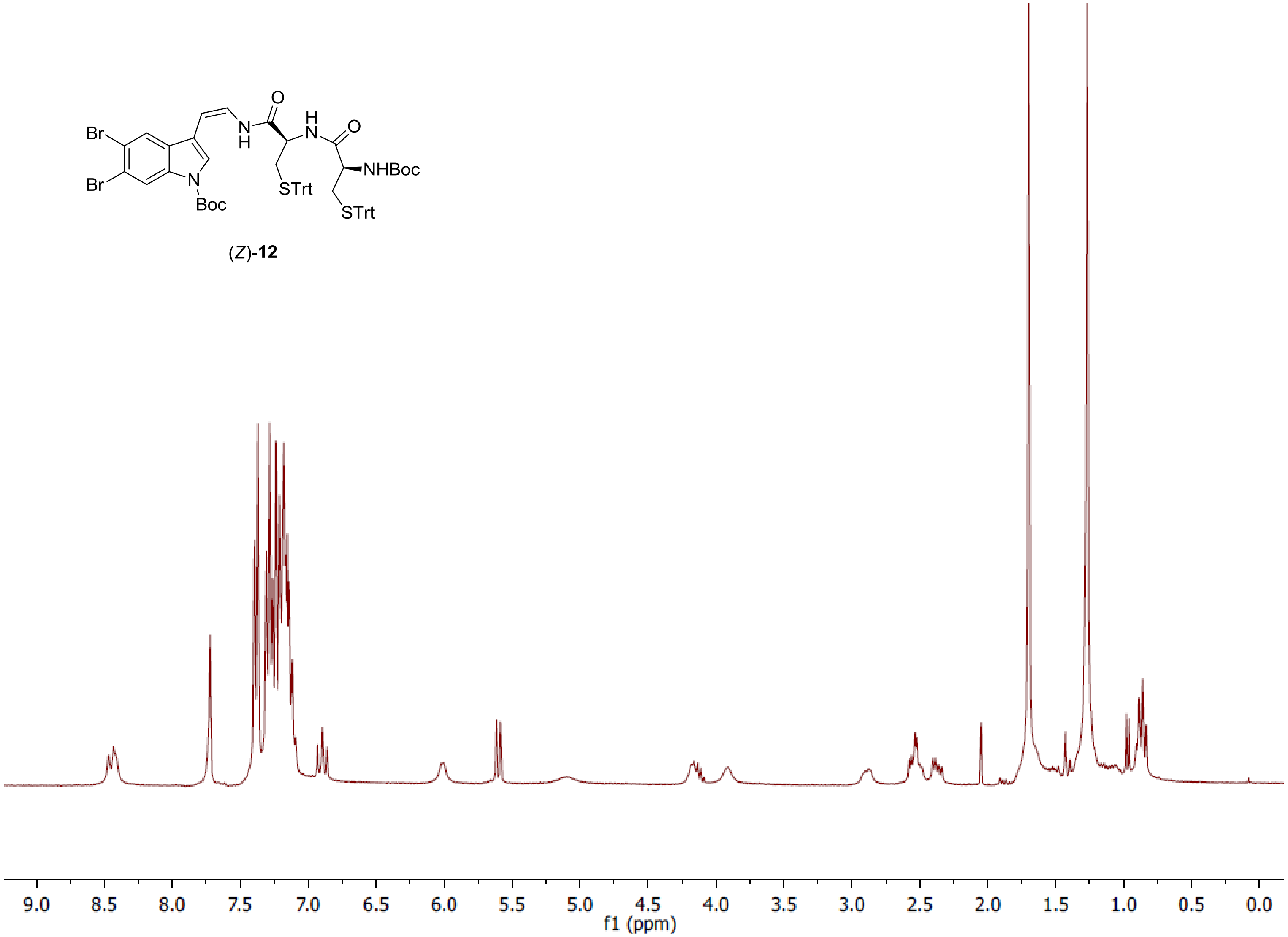

Figure S32. ^{13}C NMR spectrum of *tert*-butyl 5,6-dibromo-3-((6*R*,9*R*,*Z*)-2,2-dimethyl-4,7,10-trioxo-6,9-bis((tritylthio)methyl)-3-oxa-5,8,11-triazatridec-12-en-13-yl)-1*H*-indole-1-carboxylate (*Z*-**12**) (75 MHz, $CDCl_3$).

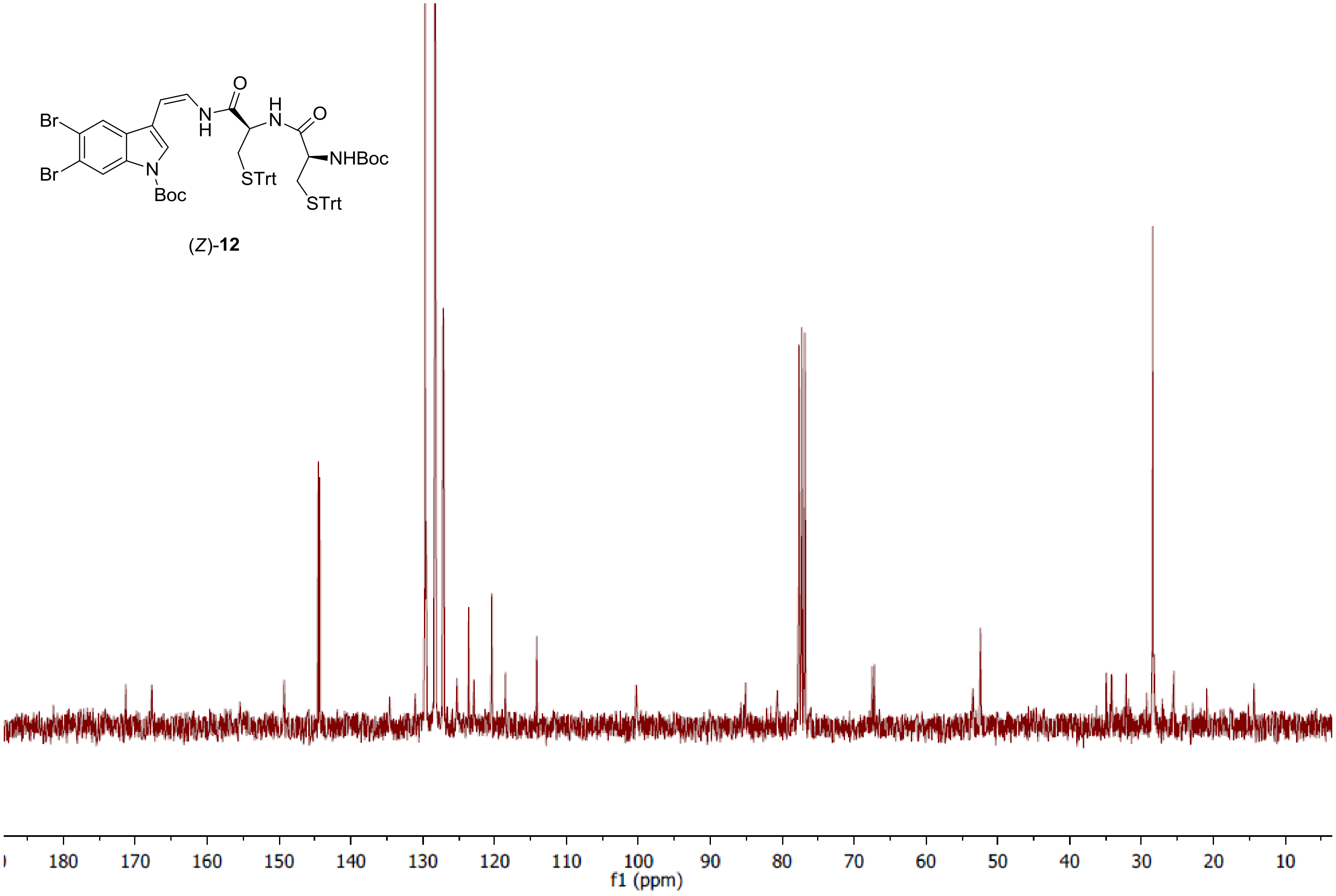

Figure S33. ^{1}H NMR spectrum of *tert*-butyl 5,6-dibromo-3-((6*R*,9*R*,*E*)-2,2-dimethyl-4,7,10-trioxo-6,9-bis((tritylthio)methyl)-3-oxa-5,8,11-triazatridec-12-en-13-yl)-1*H*-indole-1-carboxylate (*E*-**12**) (300 MHz, $CDCl_3$).

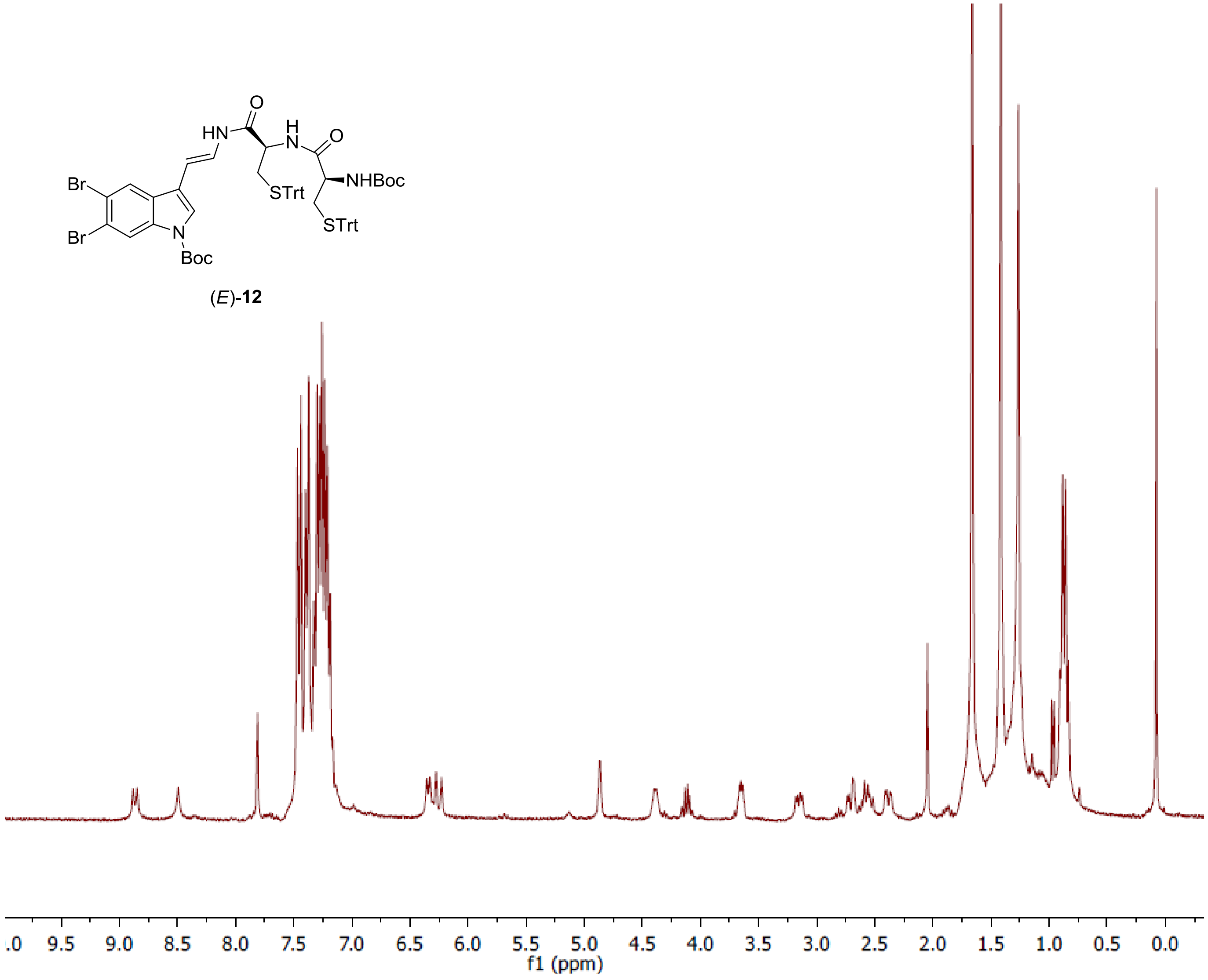

Figure S34. ^{1}H NMR spectrum of *tert*-butyl 5,6-dibromo-3-((*Z*)-2-((4*R*,7*R*)-7-((tert-butoxycarbonyl)amino)-6-oxo-1,2,5-dithiazocane-4-carboxamido)vinyl)-1*H*-indole-1-carboxylate (*Z*-**13**) (300 MHz, $CDCl_3$).

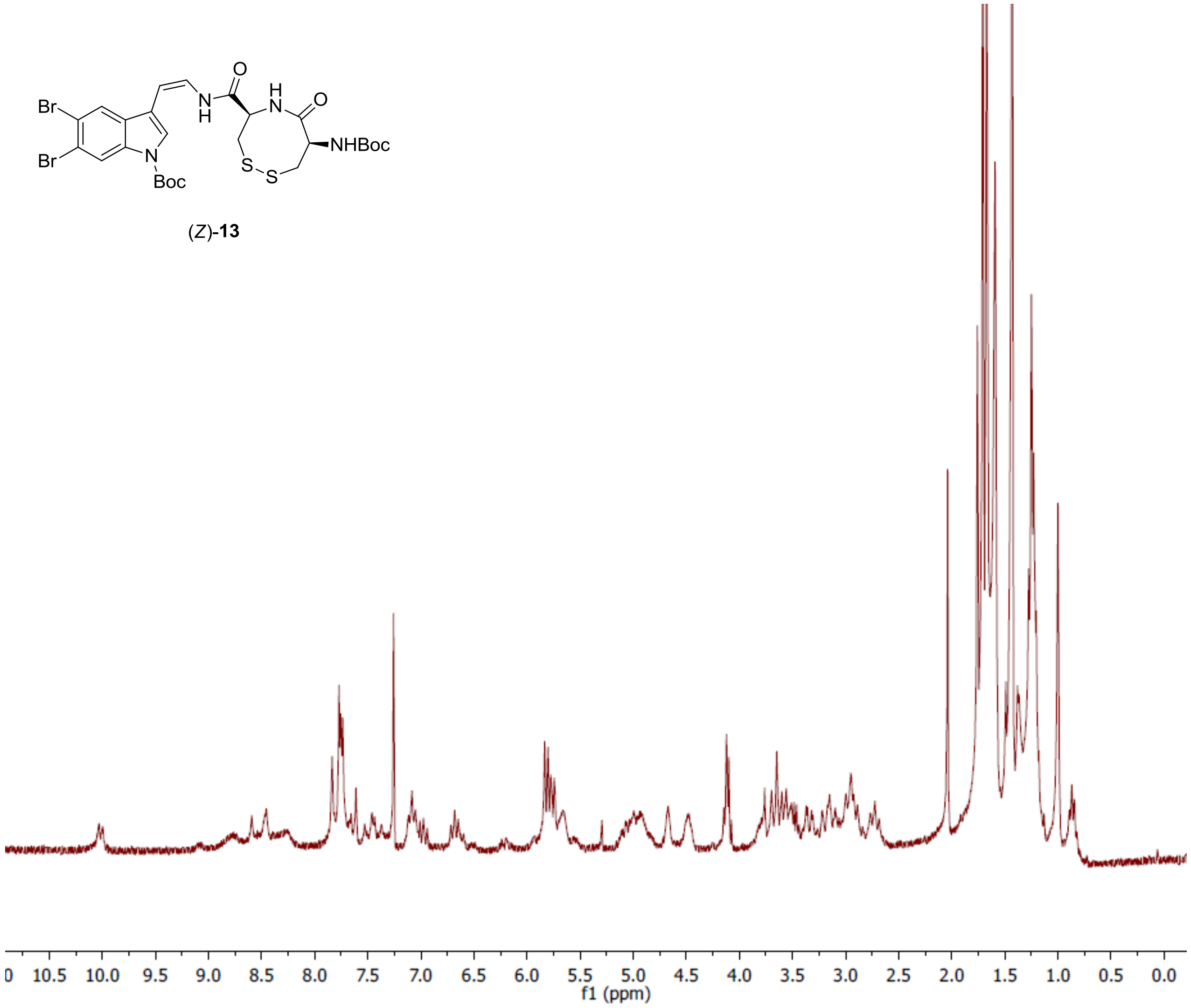

Figure S35. ^{13}C NMR spectrum of *tert*-butyl 5,6-dibromo-3-((*Z*)-2-((4*R*,7*R*)-7-((tert-butoxycarbonyl)amino)-6-oxo-1,2,5-dithiazocane-4-carboxamido)vinyl)-1*H*-indole-1-carboxylate (*Z*-**13**) (75 MHz, $CDCl_3$).

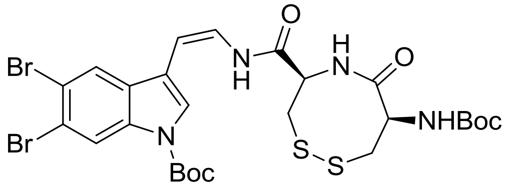

(*Z*)-**13**

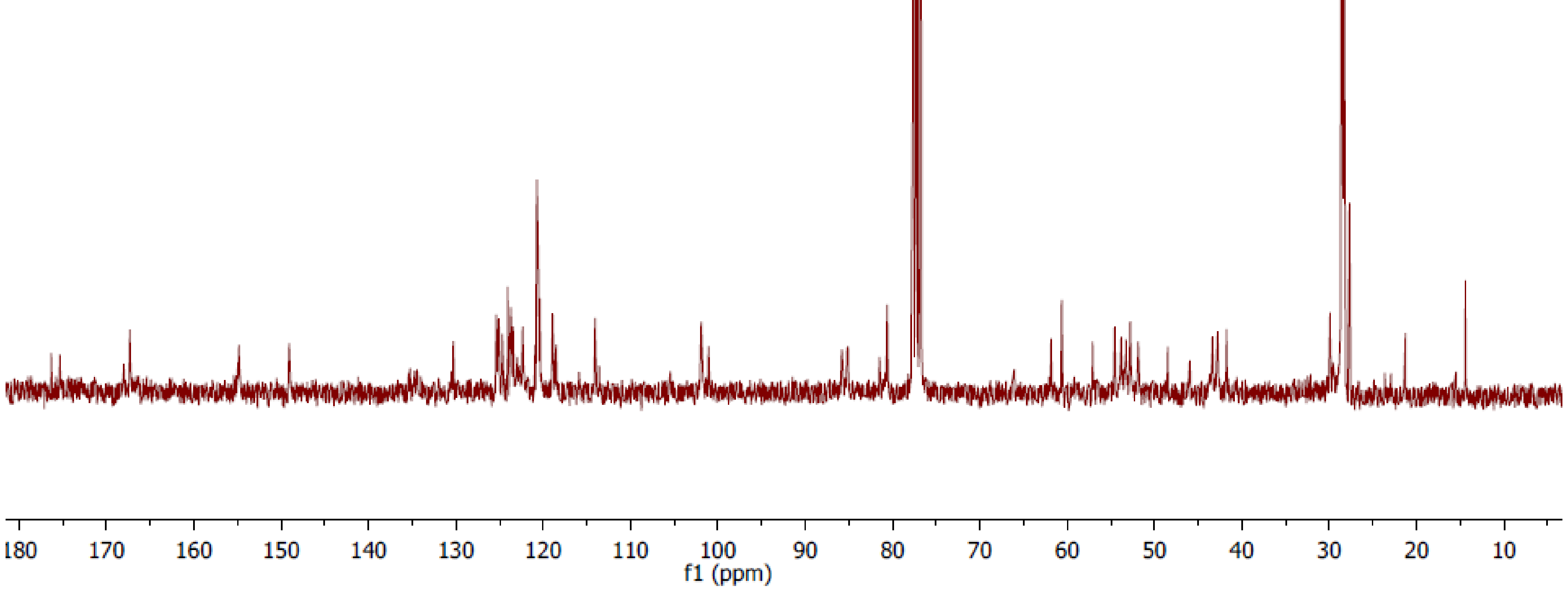

Figure S36. ^{1}H NMR spectrum of *tert*-butyl 5,6-dibromo-3-((*E*)-2-((4*R*,7*R*)-7-((tert-butoxycarbonyl)amino)-6-oxo-1,2,5-dithiazocane-4-carboxamido)vinyl)-1*H*-indole-1-carboxylate (*E*-**13**) (300 MHz, $CDCl_3$).

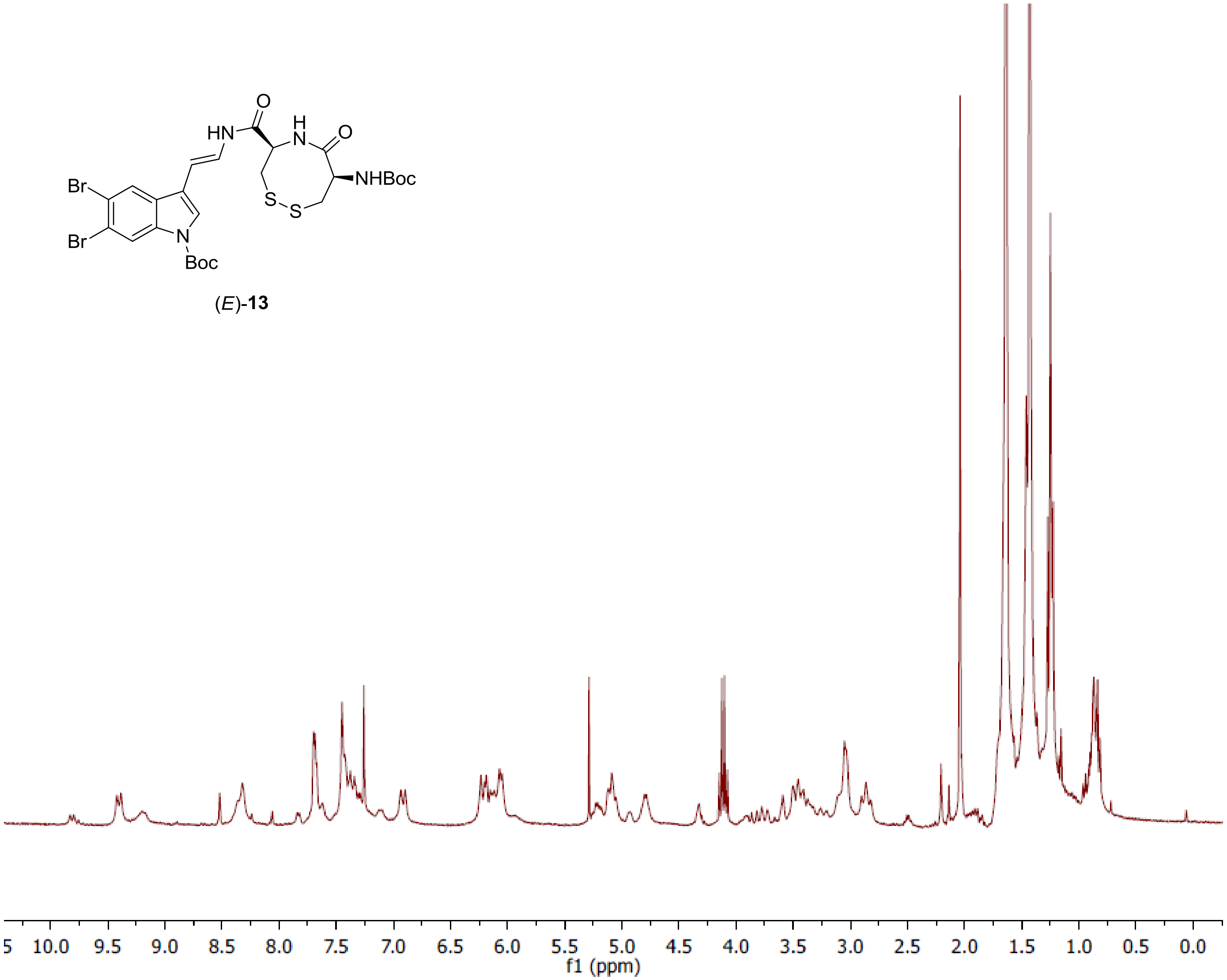

Figure S37. ^{13}C NMR spectrum of *tert*-butyl 5,6-dibromo-3-((*E*)-2-((4*R*,7*R*)-7-((tert-butoxycarbonyl)amino)-6-oxo-1,2,5-dithiazocane-4-carboxamido)vinyl)-1*H*-indole-1-carboxylate (*E*-**13**) (75 MHz, $CDCl_3$).

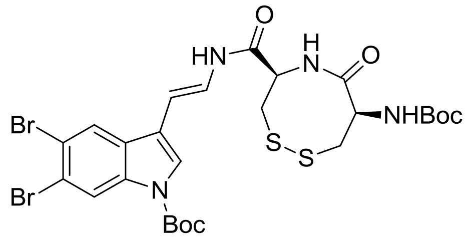

(*E*)-**13**

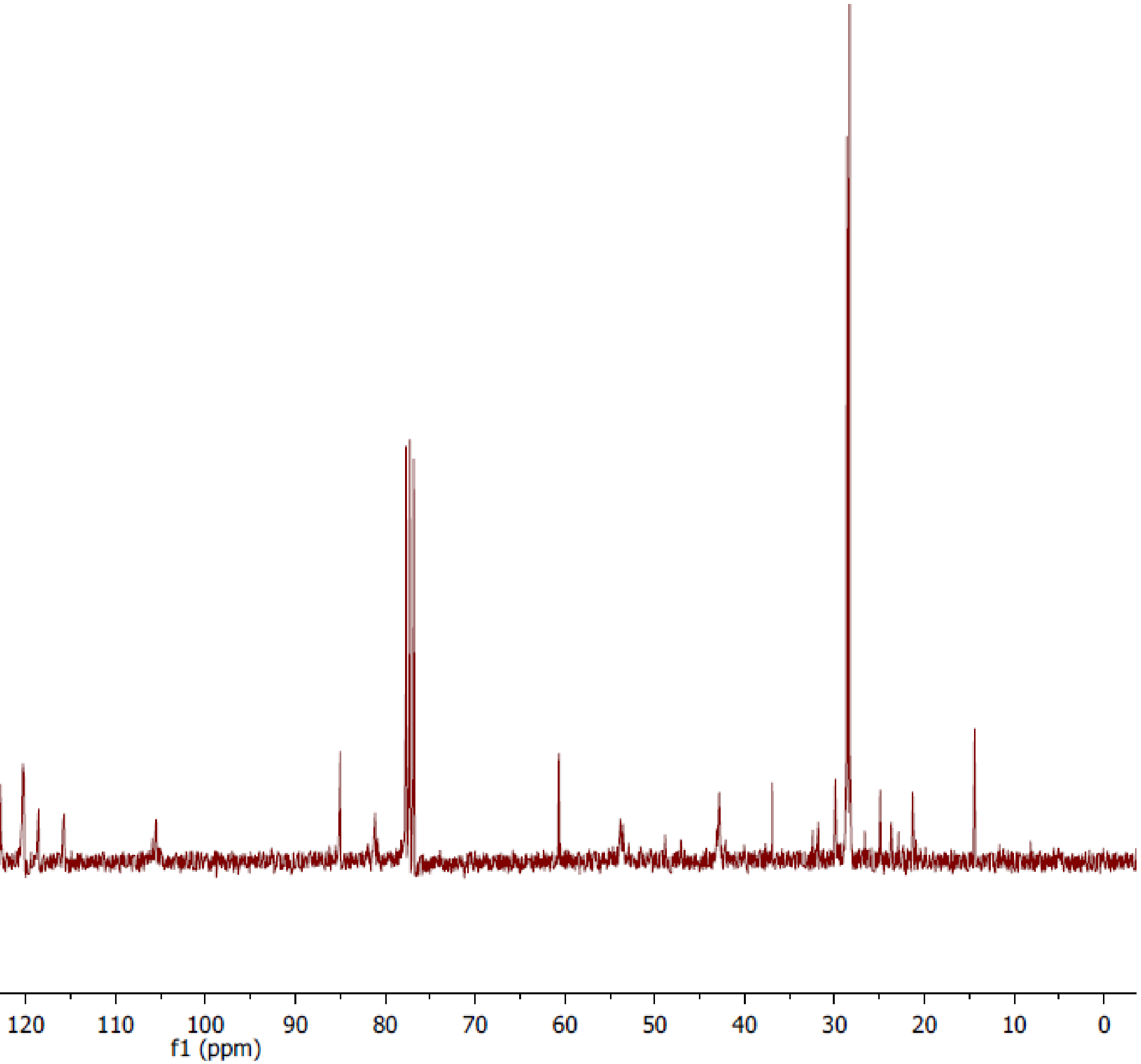

Figure S38. ^{1}H NMR spectra of synthetic *vs.* natural Tanjungide A.

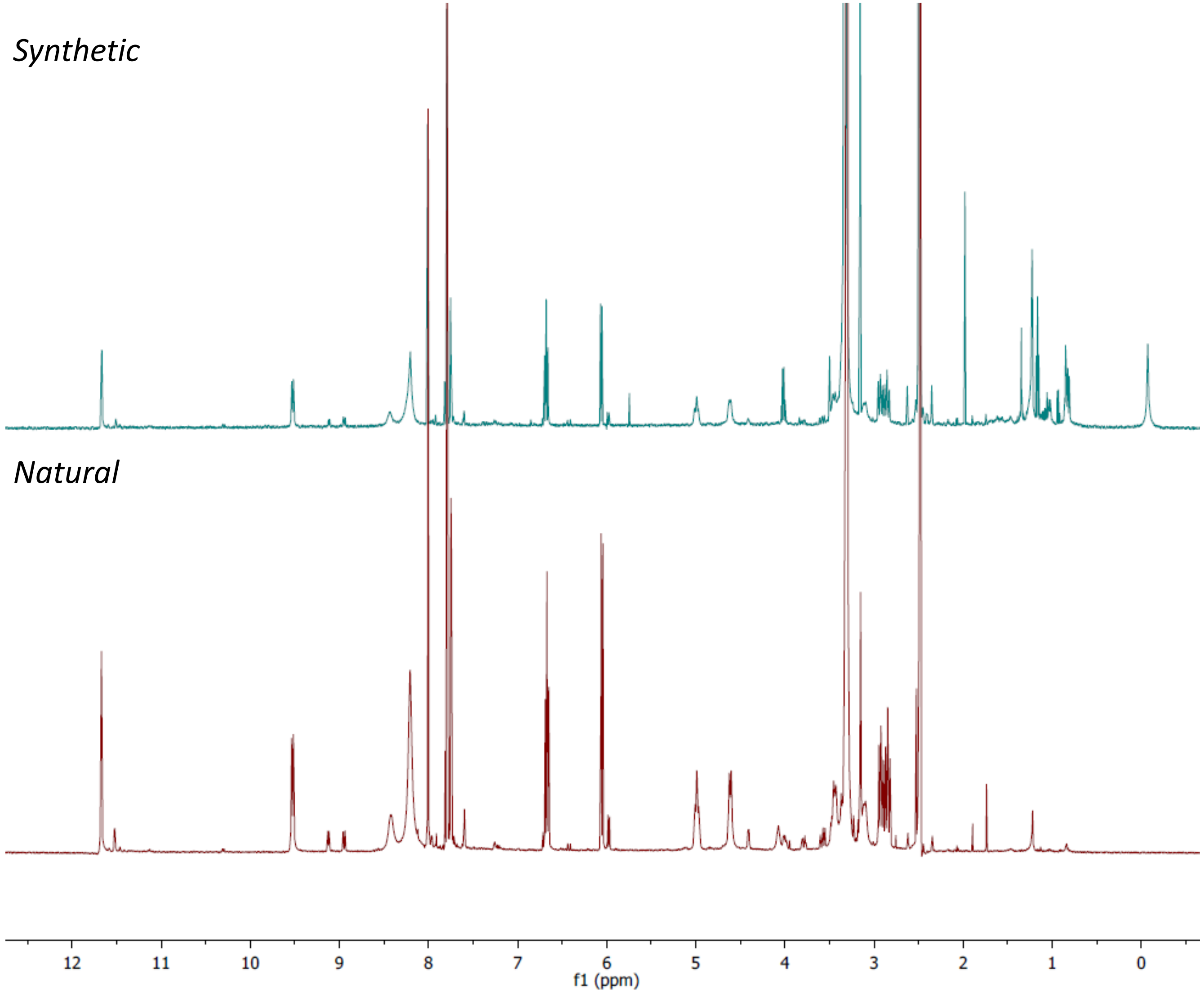

Figure S39. Marfey's reaction. Desthiotanjungide A.

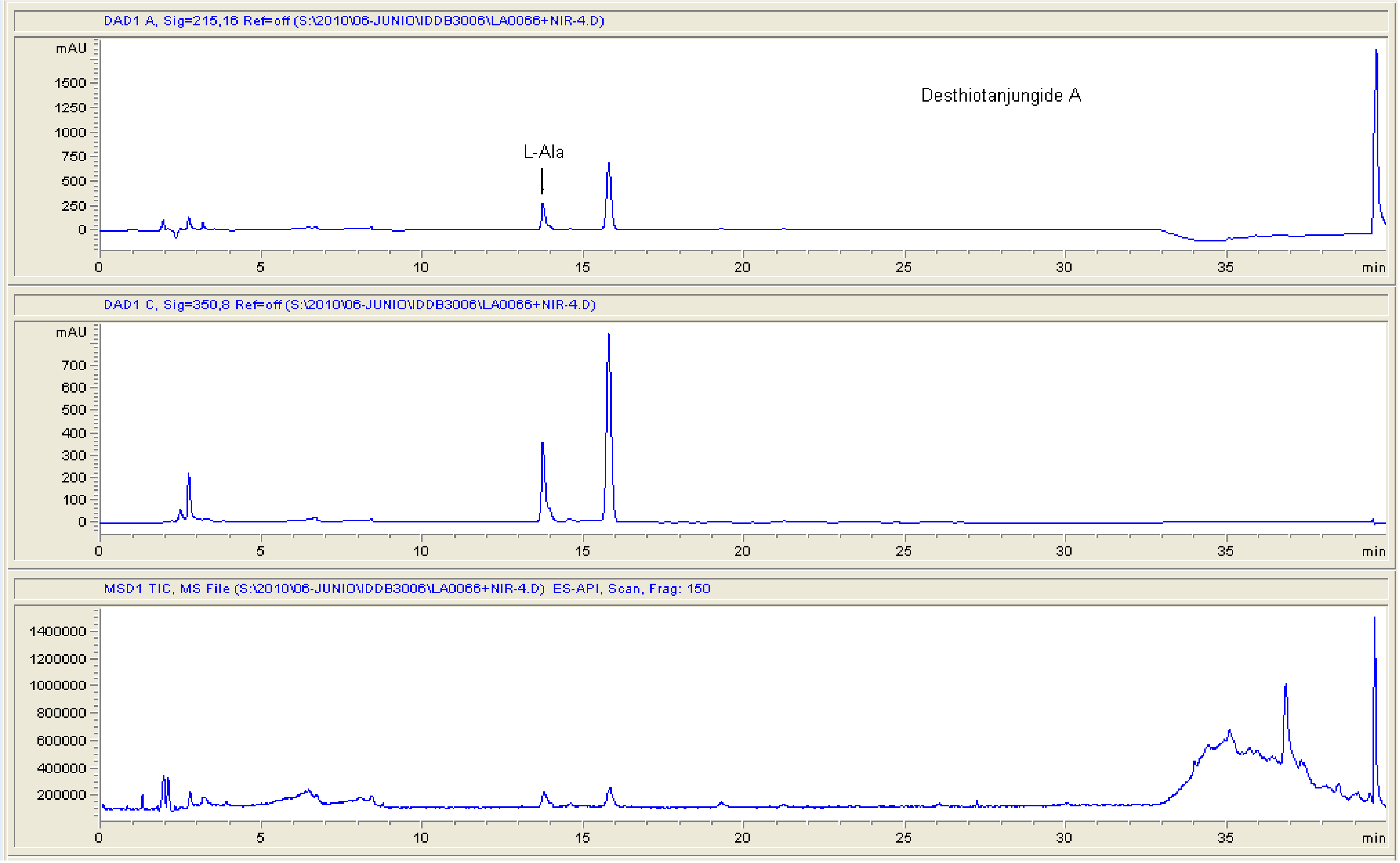

© 2014 by the authors; licensee MDPI, Basel, Switzerland. This article is an open access article distributed under the terms and conditions of the Creative Commons Attribution license (http://creativecommons.org/licenses/by/3.0/).
